# Clinical Performance Feedback Intervention Theory (CP-FIT): a new theory for designing, implementing, and evaluating feedback in health care based on a systematic review and meta-synthesis of qualitative research

**DOI:** 10.1186/s13012-019-0883-5

**Published:** 2019-04-26

**Authors:** Benjamin Brown, Wouter T. Gude, Thomas Blakeman, Sabine N. van der Veer, Noah Ivers, Jill J. Francis, Fabiana Lorencatto, Justin Presseau, Niels Peek, Gavin Daker-White

**Affiliations:** 1 Centre for Health Informatics, University of Manchester, Manchester, UK; 2 Centre for Primary Care, University of Manchester, Manchester, UK; 3 Department of Medical Informatics, Amsterdam University Medical Centres, Amsterdam, The Netherlands; 4 Department of Family and Community Medicine, University of Toronto, Toronto, Canada; 5 Centre for Health Services Research, City University of London, London, UK; 6 Centre for Implementation Research, Ottawa Hospital Research Institute, Ottawa, Canada; 7 Centre for Behaviour Change, University College London, London, UK; 8 School of Epidemiology & Public Health, University of Ottawa, Ottawa, Canada; 9 School of Psychology, University of Ottawa, Ottawa, Canada

**Keywords:** Clinical audit, Feedback, Quality improvement, Performance measurement, Qualitative research, Theory, Qualitative evidence synthesis, Learning health system

## Abstract

**Background:**

Providing health professionals with quantitative summaries of their clinical performance when treating specific groups of patients (“feedback”) is a widely used quality improvement strategy, yet systematic reviews show it has varying success. Theory could help explain what factors influence feedback success, and guide approaches to enhance effectiveness. However, existing theories lack comprehensiveness and specificity to health care. To address this problem, we conducted the first systematic review and synthesis of qualitative evaluations of feedback interventions, using findings to develop a comprehensive new health care-specific feedback theory.

**Methods:**

We searched MEDLINE, EMBASE, CINAHL, Web of Science, and Google Scholar from inception until 2016 inclusive. Data were synthesised by coding individual papers, building on pre-existing theories to formulate hypotheses, iteratively testing and improving hypotheses, assessing confidence in hypotheses using the GRADE-CERQual method, and summarising high-confidence hypotheses into a set of propositions.

**Results:**

We synthesised 65 papers evaluating 73 feedback interventions from countries spanning five continents. From our synthesis we developed Clinical Performance Feedback Intervention Theory (CP-FIT), which builds on 30 pre-existing theories and has 42 high-confidence hypotheses. CP-FIT states that effective feedback works in a cycle of sequential processes; it becomes less effective if any individual process fails, thus halting progress round the cycle. Feedback’s success is influenced by several factors operating via a set of common explanatory mechanisms: the feedback method used, health professional receiving feedback, and context in which feedback takes place. CP-FIT summarises these effects in three propositions: (1) health care professionals and organisations have a finite capacity to engage with feedback, (2) these parties have strong beliefs regarding how patient care should be provided that influence their interactions with feedback, and (3) feedback that directly supports clinical behaviours is most effective.

**Conclusions:**

This is the first qualitative meta-synthesis of feedback interventions, and the first comprehensive theory of feedback designed specifically for health care. Our findings contribute new knowledge about how feedback works and factors that influence its effectiveness. Internationally, practitioners, researchers, and policy-makers can use CP-FIT to design, implement, and evaluate feedback. Doing so could improve care for large numbers of patients, reduce opportunity costs, and improve returns on financial investments.

**Trial registration:**

PROSPERO, CRD42015017541

**Electronic supplementary material:**

The online version of this article (10.1186/s13012-019-0883-5) contains supplementary material, which is available to authorized users.

## Background

Providing health professionals with quantitative summaries of their clinical performance when treating specific groups of patients has been used for decades as a quality improvement strategy (Table 1) [1]. Such approaches may be called “audit and feedback”, “clinical performance feedback”, “performance measurement”, “quality measurement”, “key performance indicators”, “quality indicators”, “quality dashboards”, “scorecards”, “report cards”, or “population health analytics” [2–4]. In this paper, we use the term “feedback” intervention to encompass all these approaches and to refer to the entire process of selecting a clinical topic on which to improve, collecting and analysing population-level data, producing and delivering a quantitative summary of clinical performance, and making subsequent changes to clinical practice.

**Table 1:** Examples of feedback interventions used in health care

Example	
A group of clinicians choose a clinical topic on which to focus (e.g. sepsis, frailty), determine standards of care relating to that topic (e.g. patients with sepsis should receive antibiotics within 1 hour of diagnosis, all patients with frailty should have an annual medication review), then collect data to measure their current performance (e.g. from medical records), and calculate the proportion of patients meeting the standards. They present their findings to colleagues in a team meeting, and as a group they identify and implement changes. They re-measure their performance at a later date.	
Health managers decide goals that are most important to their organisation (e.g. reducing hospital admissions, increasing vaccination uptake, reducing medication safety errors) and collect data to measure their current performance (e.g. from patient registries or administrative data). To account for the influence of patient characteristics, the results are adjusted for age and sex. These data are sent to health professionals as reports (e.g. electronic documents) and may also be made publically available. There may be financial rewards associated with achieving particular levels of performance.	
Population-level data from electronic sources are automatically extracted and analysed across a range of topics (e.g. rates of antibiotic prescription, proportion of hypertensive patients with controlled blood pressure) to identify patients not receiving “optimal” care (e.g. from electronic health records). Results are continuously updated, and communicated via software to health professionals (e.g. as bar charts or line graphs via websites or desktop applications).	

Feedback has been extensively researched in numerous quantitative and qualitative studies [5]. However, despite its popularity, the mechanisms by which it operates are poorly understood [5]. In this paper, we define mechanisms as underlying explanations of how and why an intervention works [6]. Three consecutive Cochrane reviews have found feedback produces “small but potentially important improvements in professional practice” [7] with wide variations in its impact: the most recent demonstrated a median clinical practice improvement of 4.3%, ranging from a 9% decrease to a 70% increase [8]. When feedback interventions target suboptimally performed high-volume and clinically impactful practices, such as hypertension management or antimicrobial stewardship, this variation can translate to thousands of quality-adjusted life years [9, 10].

Policymakers and practitioners only have a tentative set of best practices regarding how feedback could be optimally conducted [5, 11]; thus there is a need to better understand how and why feedback works in order to maximise their impact [5, 7]. One approach is to consider the underlying theory of feedback, which has often been over-looked [5, 12]. In this paper, we define theory as a “coherent description of a process that is arrived at by inference, provides an explanation for observed phenomena, and generates predictions” [13]. In the 140 randomised controlled trials in the most recent Cochrane review, 18 different theories were used in only 20 (14%) of the studies, suggesting a lack of consensus as to which is most appropriate for feedback [12]. More recently, three theories have gained popularity in the feedback literature: [5] Control Theory [14], Goal Setting Theory [15], and Feedback Intervention Theory [16]. However, these theories address only part of the feedback process, and even if used in conjunction may still miss potentially important factors specific to health care (Table 2).

**Table 2:** Popular theories to model clinical performance feedback in the literature [5]

Theory name and description	Covers the entire feedback process				Includes important factors in health care quality improvement		
	Selecting clinical topics	Collecting and analysing data	Producing and delivering feedback	Making changes to clinical practice	Team-based change [5]	Context [123]	Intervention implementation [17]
*Control Theory* [14]							
Proposes that behaviour is regulated by a negative feedback loop, in which a person’s perception of their current state is compared against a goal. People strive to reduce perceived discrepancies between the two by modifying their behaviour.	No	No	No	No	No	No	No
*Goal Setting Theory* [15]							
Explains how goals (defined as the object or aim of an action) affect task performance and how performance can be influenced by factors including commitment, goal importance, self-efficacy, feedback, and task complexity.	Yes	No	No	No	No	No	No
*Feedback Intervention Theory* [16]							
Describes how feedback can influence behaviour and describes factors that determine whether feedback has a positive or negative influence on performance. Factors include feedback intervention cues; task characteristics; and situational variables (including personality). Feedback Intervention Theory draws upon ideas in both Control Theory and Goal Setting Theory.	Somewhat	No	Yes	No	No	No	No

Qualitative evaluations of quality improvement interventions can generate hypotheses regarding their effect modifiers (i.e. variables that influence success) and mechanisms of action [17]. For example, by helping explain why a particular intervention was ineffective (e.g. [18]), or developing a logic model for success (e.g. [19]). Synthesising findings from different qualitative studies can help build theories of how interventions may be optimally designed and implemented [20]. Such approaches have been used to improve interventions in tuberculosis therapy [21], smoking cessation [22], skin cancer prevention [23], and telephone counselling [24]. A similar approach may therefore be useful for feedback and, to the best of our knowledge, has not yet been attempted.

### Aims and objectives

We aimed to synthesise findings from qualitative research on feedback interventions to inform the development of a comprehensive new health care-specific feedback theory. Informed by our definition of theory [13], our objectives were to (1) describe the processes by which feedback interventions effect change in clinical practice, (2) identify variables that may predict the success of these processes, (3) formulate explanatory mechanisms of how these variables may operate, and (4) distil these findings into parsimonious propositions.

## Methods

We published our protocol on the International Prospective Register of Systematic Reviews (PROSPERO; registration number CRD42015017541 [25]).

### Search strategy

We replicated the latest Cochrane review’s search strategy [8], adding qualitative research filters [26–28] (Additional file 1). MEDLINE (Ovid), EMBASE (Ovid), and CINAHL (Ebsco) were searched without time limits on 25 March 2015. Citation, related article, and reference list searches were undertaken up to 31 December 2016 for all included studies, relevant reviews, and essays (e.g. [5, 11, 12, 29–37]) [38]. Further studies were found through international experts and Google Scholar alerts.

### Study selection and data extraction

Table 3 describes our inclusion criteria. Two reviewers independently screened titles and abstracts. Full manuscripts of potentially relevant citations were obtained and the criteria re-applied. Data from included articles were extracted independently by BB and WG regarding the study [39] and feedback intervention details [40, 41] (Additional file 2; e.g. study setting, who provided the feedback, and what information the feedback contained). Critical appraisal was conducted concurrently using 12 technical and theoretical criteria including the appropriateness of data collection and analysis methods, adequacy of context description, and transferability of findings [42]. Any disagreements were resolved through discussion, with the wider team consulted as necessary.

**Table 3:** PICOS inclusion criteria and example exclusions

Inclusion criteria	Typical exclusion examples
*Population*	
The intervention primarily targeted health professionals (including clinicians and non-clinicians e.g. managers) [8].	Interventions intended to help patients choose health care provider or treatment (e.g. [124]).
*Intervention*	
The intervention provided feedback to participants [8].	Audit reports (e.g. [125]); pay-for-performance programmes where feedback was not explicitly provided (e.g. [126]).
Feedback primarily concerned health professionals’ performance in clinical settings, defined as compliance with pre-defined clinical standards (e.g. clinical guidelines) and/or achievement of clinical patient outcomes [8]. This may have referred to the performance of an individual, their team, or organisation [8].	Interventions that provided only fictitious feedback (e.g. [127]), feedback used in training or simulated settings (e.g. [128]), feedback on non-clinical aspects of performance, or data not directly related to clinical performance, such as costs of care (e.g. [129]), patient experience (e.g. [130]), or epidemiological surveillance (e.g. [131]).
Clinical performance data were primarily measured from medical records, computerised databases, or observations from patients [2, 132].	Feedback based only on peer or supervisor observation (e.g. [133]).
Feedback related to care provided to defined populations of patients [2, 8, 134].	Feedback solely on the care of individual patients, such as reminder or alert systems (e.g. [135]), patient-level summaries (e.g. [136]), significant event analyses, or case reviews (e.g. [137]).
Feedback could inform quality improvement actions for teams or organisations, not solely individual patients [2, 134].	Dashboards that summarised patients’ current clinical status to primarily inform point-of care decisions (e.g. [138]).
Feedback was a core and essential component of the intervention i.e. in multifaceted interventions was unlikely other components would have been offered in the absence of feedback [8, 132].	Improvement collaboratives that primarily consisted of mentoring visits, improvement advisors, and educational sessions, with “benchmarking” as an additional component (e.g. [139]).
*Comparator*	
Not applicable	Not applicable
*Outcome*	
The intervention primarily aimed to improve clinical performance (as defined) [8].	Interventions that primarily intended to reduce costs (e.g. [129]).
*Study*	
Studies of specific interventions described in enough detail to determine whether they met the above criteria.	Studies of groups or collections of interventions, the characteristics of which are not clearly described. For example, studies of “feedback interventions” in general (e.g. [140]).
Evaluations of feedback interventions that reported both qualitative data collection (e.g. semi-structured interviews, focus groups, unstructured observations) and analysis methods (e.g. grounded theory, thematic analysis, framework analysis) [141]. They must have provided either a full methodological description or reference to a specific relevant approach [141]. Studies could seek to answer *any* research question about the feedback intervention.	Studies reporting interviews or focus groups but no description of analytic methods (e.g. [142]), intervention descriptions or protocol papers (e.g. [143]), editorials or opinion papers (e.g. [144]), quantitative surveys with or without open ended questions (e.g. [145]), or manuscripts with insufficient detail to judge adequacy, such as abstracts or letters (e.g. [146]).
Peer-reviewed publications in scholarly journals written in English.	Books, grey literature, theses (e.g. [147]).

### Data synthesis

**Fig. 1:**
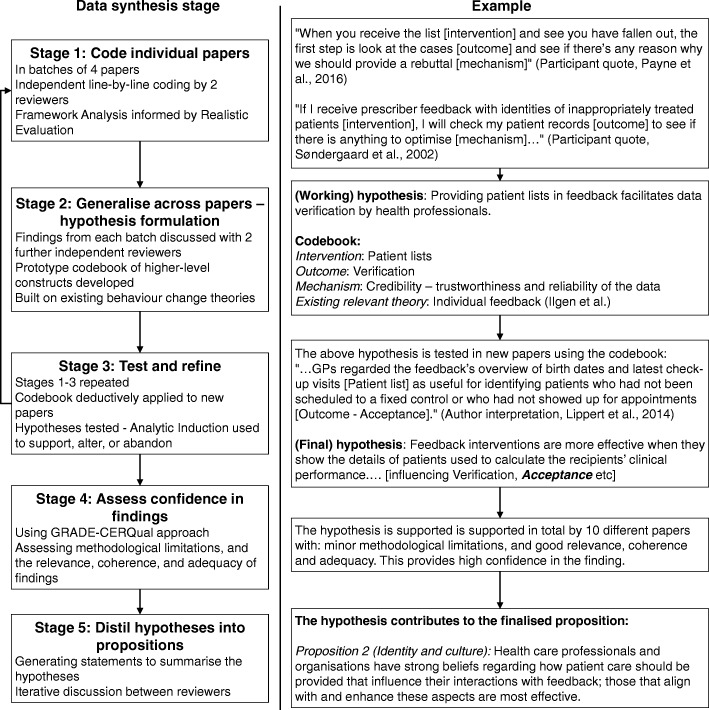
Data synthesis process

Study findings were extracted as direct quotations from participants and author interpretations [143, 43] found in the abstract, results, and discussion sections. Data were synthesised in five stages (Fig. 1; please see Additional file 3 for details): coding excerpts from individual papers in batches using framework analysis [44] and realistic evaluation [6], generalising findings across papers [45] and building on pre-existing theories to formulate hypotheses [36], iteratively testing and improving these hypotheses on new batches of papers using Analytic Induction [46], assessing confidence in our hypotheses using the GRADE-CERQual method [47], and summarising high-confidence hypotheses into a core set of propositions.

## Results

### Study characteristics

**Fig. 2:**
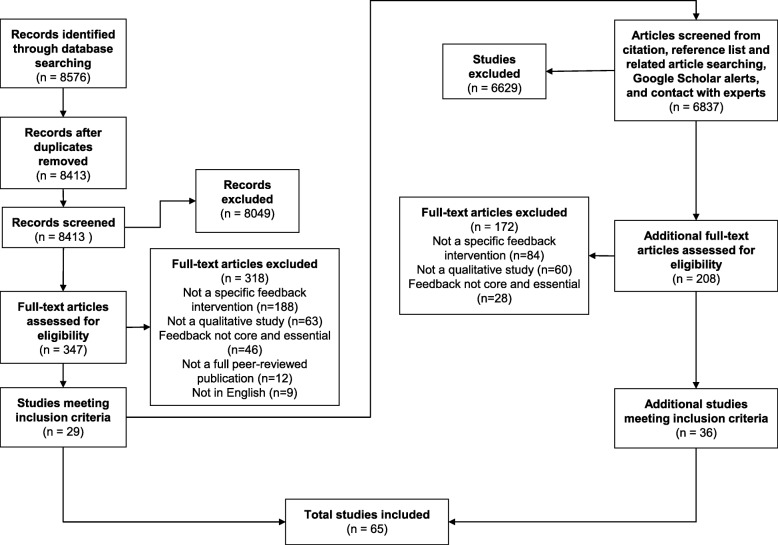
Flowchart of study screening process

We screened 15,413 papers (Fig. 2). Sixty-five were ultimately included, reporting 61 studies of 73 feedback interventions involving 1791 participants, from which we synthesised 1369 excerpts. Table 4 summarises their main characteristics, full details of which are in Additional file 4.

**Table 4:** Frequency of main paper characteristics

	Count (%)*
Publication date	
2012–2016	42 (65)
2007–2011	13 (20)
2002–2006	4 (6)
1996–2001	6 (9)
Quality appraisal	
No limitations	0 (0)
Minor limitations	9 (14)
Moderate limitations	47 (72)
Major limitations	9 (14)
Continent	
Europe	37 (57)
North America	22 (34)
Africa	2 (3)
Australia	2 (3)
South America	2 (3)
Setting	
Hospital inpatient	30 (46)
Primary care	28 (43)
Hospital outpatient	3 (5)
Nursing home	3 (5)
Mental health	1 (2)
Feedback topic	
Chronic care (general)	15 (23)
Patient experience	14 (22)
Prescribing	11 (17)
Health care structures	10 (15)
General nursing	8 (12)
Surgery	7 (11)
Cancer	5 (8)
Diabetes	5 (8)
Stroke	5 (8)
Obstetrics	5 (8)
Preventive care	4 (6)
Infectious disease	3 (5)
Patient demographics	2 (3)
Staff experience	2 (3)
Intensive care	2 (3)
Mental health	1 (2)
General surgery	1 (2)
Heart failure	1 (2)
Orthopaedics	1 (2)
Paediatrics	1 (2)
Physiotherapy	1 (2)
Rheumatology	1 (2)
Care costs	1 (2)
Feedback recipient	
Physicians	45 (69)
Nurses	40 (62)
Non-clinicians	24 (37)
Surgeons	6 (9)
Allied clinicians	6 (9)
Junior physicians	3 (5)
Midwives	2 (3)
Pharmacists	2 (3)
Pathologists	1 (2)
Radiologists	1 (2)
Feedback format	
Paper report	28 (43)
Face-to-face	25 (38)
Software application	12 (18)
Electronic report	10 (15)
Co-intervention	
Peer discussion	28 (43)
Problem solving	22 (34)
External change agent	17 (26)
Action planning	15 (23)
Reward (financial)	13 (20)
Clinical education	7 (11)
Reward (non-financial)	5 (8)
Reminders	3 (5)

*Counts may add to more than 100% where papers are in multiple categories

### Meta-synthesis: Clinical Performance Feedback Intervention Theory (CP-FIT)

**Fig. 3:**
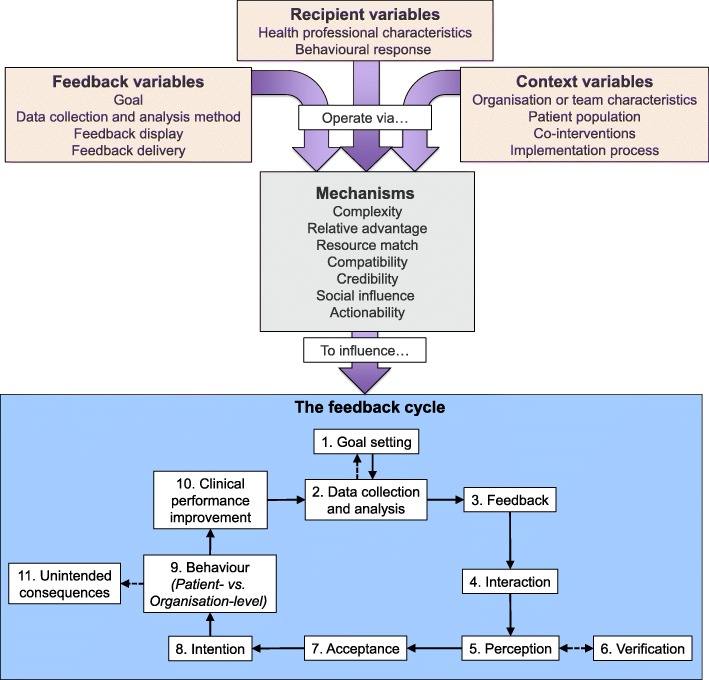
Clinical Performance Feedback Intervention Theory’s variables and explanatory mechanisms, and their influence on the feedback cycle. Solid arrows are necessary pathways for successful feedback. Dotted arrows represent potential pathways

From our synthesis, we developed Clinical Performance Feedback Intervention Theory (CP-FIT). CP-FIT argues that effective feedback works in a cycle, the success of progressing round which is influenced by variables operating through a set of common explanatory mechanisms related to the feedback itself, the recipient, and wider context (Fig. 3). How these variables and mechanisms influence the feedback cycle is illustrated by 42 high-confidence hypotheses (Table 5), which are in turn summarised by three propositions (Table 6). CP-FIT draws on concepts from 30 pre-existing behaviour change theories (Table 7) and has over 200 lower confidence hypotheses (Additional file 5).

**Table 5:** Forty-two high-confidence hypotheses from Clinical Performance Feedback Intervention Theory

Hypothesis: Feedback interventions are more effective when …	Relevant feedback cycle process(es)	Key explanatory mechanism(s)	Illustrative paper reference
*Feedback variables*			
*Goal*			
1. *Importance*: … They focus on goals recipients believe to be meaningful and often do not happen in practice.	*Acceptance*, *Intention*	*Compatibility*, *Credibility*	
2. *Controllability*: … They focus on goals perceived to be within the control of the recipients.	*Acceptance*, *Intention*	*Actionability*	[62]
3. *Relevance*: … They focus on goals perceived as relevant to recipients’ jobs.	*Acceptance*, *Intention*	*Actionability*, *Compatibility*, *Relative advantage*	[64]
*Data collection and analysis method*			
4. *Conducted by recipients*: … They do not require the recipient to collect or analyse the clinical performance data. (Can also decrease 41. *Cost*)	*Data collection and analysis*	*Complexity*, *Resource match*	[67]
5. *Automation*: … They collect and analyse data automatically rather than manually.	*Data collection and analysis*	*Complexity*, *Resource match*	[68]
6. *Accuracy*: … They use data believed by recipients to be a true representation of their clinical performance.	*Acceptance*	*Credibility*, *Relative advantage*	[50]
7. *Exclusions*: … They allow recipients to exception report patients they feel are inappropriate to include in their performance measurement.	*Acceptance*	*Actionability*, *Credibility*	[70]
*Feedback display*			
8. *Performance level*: … They communicate recipients’ current performance has room for improvement.	*Intention*, *Behaviour*	*Actionability*, *Compatibility*	[64]
9. *Patient lists*: … They show the details of patients used to calculate the recipients’ clinical performance.	*Verification*, *Acceptance*, *Perception*, *Intention*, *Behaviour*	*Actionability* *Complexity* *Credibility*	[50]
10. *Specificity*: … They report the performance of individual health professionals rather than their wider team or organisation.	*Acceptance*, *Intention*, *Behaviour*	*Actionability*	[72]
11. *Timeliness*: … They use recent data to calculate recipients’ current performance.	*Acceptance*, *Intention*, *Behaviour*	*Actionability*, *Credibility*	[50]
12. *Trend*: … They show recipients’ current performance in relation to their past performance. (Can also increase 40. *Observability*)	*Perception*	*Complexity*, *Relative advantage*	[73]
13. *Benchmarking*: … They compare recipients’ current performance to that of other health professionals, organisations or regions.	*Perception*, *Intention*, *Behaviour*	*Complexity*, *Social influence*	[74]
14. *Prioritisation*: … They communicate the relative importance of feedback contents.	*Perception*	*Complexity*, *Relative advantage*	[55]
15. *Usability*: … They employ user-friendly designs. (Can also increase 40. *Observability*)	*Perception*	*Complexity*	[82]
*Feedback delivery*			
16. *Function*: … They are perceived to support positive change rather than punish suboptimal performance.	*Acceptance*	*Compatibility*	[85]
17. *Source knowledge and skill*: … They are delivered by a person or organisation perceived to have an appropriate level of knowledge or skill.	*Acceptance*	*Credibility*, *Social influence*	[86]
18. *Active delivery*: … They “push” feedback messages to recipients rather than requiring them to “pull”. (Except if solely delivered face-to-face, which increases 41. *Cost*)	*Interaction*	*Compatibility*, *Complexity*	
19. *Delivery to a group*: … They deliver feedback to groups of recipients.	*Perception*, *Intention*, *Behaviour* (by increasing 28. *Teamwork*)	*Social influence*	[98]
*Recipient variables*			
*Health professional characteristics*			
20. *Feedback attitude*: … They target health professionals with positive beliefs about feedback.	All	*Compatibility*, *Relative advantage*	[64]
21. *Knowledge and skills in quality improvement*: … They target health professionals with greater capability in quality improvement.	*Perception*, *Intention*, *Behaviour*	*Actionability*, *Complexity*, *Resource match*	[91]
22. *Knowledge and skills in clinical topic*: … They target health professionals with greater capability in the clinical topic under focus.	*Perception*, *Intention*, *Behaviour*	*Actionability*, *Resource match*	[92]
*Behavioural response*			
23. *Organisation-level* and *Patient-level behaviour*: … Health professionals undertake changes involving the wider health care system rather than just individual patients in response to feedback. (Can also increase 24. *Resource*)	*Clinical performance improvement*	*Actionability*	[95]
*Context variables*			
*Organisation or team characteristics*			
24. *Resource*: … Organisations and teams have greater capacity to engage with them. (Can also increase 23. *Organisation-level behaviour*)	All	*Resource match*	[98]
25. *Competing priorities*: … Organisations and teams have minimal additional responsibilities.	All	*Resource match*, *Compatibility*	[90]
26. *Leadership support*: … They are supported by senior managers. (Can also increase 23. *Organisation-level behaviour*)	All	*Credibility*, *Resource match*, *Social influence*	[87]
27. *Champions*: … They are supported by individuals in the organisation dedicated to making it a success.	All	*Credibility*, *Resource match*, *Social influence*	[68]
28. *Teamwork*: … They are implemented into organisations or teams whose members work together towards a common goal.	*Perception*, *Intention*, *Behaviour*	*Actionability*, *Resource match*, *Social influence*	[72]
29. *Intra-organisational networks*: … They are implemented into organisations or teams with strong internal communication channels.	*Interaction*, *Perception*, *Intention*, *Behaviour*	*Actionability*, *Compatibility*, *Resource match*, *Social influence*	[51]
30. *Extra-organisational networks*: … They are implemented into organisations or teams that actively communicate with external bodies.	*Perception*, *Intention*, *Behaviour*	*Actionability* *Resource match*	[86]
31. *Workflow fit*: … They fit alongside existing ways of working.	All	*Compatibility*, *Complexity*	[64]
*Patient population*			
32. *Choice alignment*: … They do not include patients who refuse aspects of care measured in the feedback in their calculations.	*Acceptance*, *Intention*	*Actionability*, *Compatibility*, *Complexity*	[105]
33. *Clinical appropriateness*: … They do not include patients whose care cannot be safely optimised further.	*Acceptance*, *Intention*	*Actionability*, *Compatibility*, *Complexity*	[148]
*Co-interventions*			
34. *Peer discussion*: … They encourage recipients discuss their feedback with peers. (Can also increase 28. *Teamwork*)	*Perception*, *Intention*	*Complexity*, *Resource match*, *Social influence*	[89]
35. *Problem solving*: … They help recipients identify and develop solutions to reasons for suboptimal performance (or support recipients to do so).	*Perception*	*Actionability*, *Compatibility*, *Complexity*, *Resource match*	[90]
36. *Action planning*: … They provide solutions to suboptimal performance (or support recipients to do so).	*Intention*, *Behaviour*	*Actionability*, *Complexity*, *Resource match*	[62]
37. *External change agents*: … They provide additional staff to explicitly support its implementation.	*All*	*Resource match*	[94]
*Implementation process*			
38. *Adaptability*: … They are tailored to the specific needs of the health care organisation and its staff. (Can also increase 31. *Workflow fit*)	All	*Compatibility*, *Complexity*	[69]
39. *Training and support*: *…* They provide training and support regarding feedback (not the clinical topic under scrutiny).	*Perception*, *Intention*, *Behaviour* (by increasing 21. *Knowledge and skills in quality improvement*)	*Actionability*, *Resource match*	[91]
40. *Observability*: … They demonstrate their potential benefits to recipients.	All	*Relative advantage*	[88]
41. *Cost*: … They are considered inexpensive to deploy in terms of time, human or financial resources.	All	*Resource match*	[67]
42. *Ownership*: … Recipients feel they “own” it, rather than it has been imposed on them.	All	*Compatibility*	[149]

**Table 6:** Clinical Performance Feedback Intervention Theory’s three propositions

Proposition	Relevant explanatory mechanism(s)	Key example hypotheses*
1. *Capacity limitations* Health care professionals and organisations have a finite capacity to engage with and respond to feedback; interventions that require less work, supply additional resource, or are considered worthwhile enough to justify investment, are most effective.	Complexity Relative advantage Resource match	5. *Automation* 15. *Usability* 18. *Active delivery*
2. *Identity and culture* Health care professionals and organisations have strong beliefs regarding how patient care should be provided that influence their interactions with feedback; those that align with and enhance these aspects are most effective.	Compatibility Credibility Social influence	1. *Importance* 6. *Accuracy* 13. *Benchmarking*
3. *Behavioural induction* Feedback interventions that successfully and directly support clinical behaviours for individual patients are most effective.	Actionability	2. *Controllability* 11. *Timeliness* 34. *Problem solving*

*Numbers refer to Table 4. For brevity, only key example hypotheses from Table 4 are provided. Each hypothesis from Table 4 can be mapped to a specific proposition by cross-referencing its relevant mechanisms

**Table 7:** Thirty pre-existing behaviour change theories that contribute to Clinical Performance Feedback Intervention Theory

Theory	Contributes to the following constructs … *
Context and implementation theories	
Diffusion of innovations [108]	Variables: *Observability* Mechanisms: *Compatibility*, *Complexity*, *Relative advantage*
Diffusion of innovations in health service delivery and organisation [65]	Variables: *Champion*, *Extra-organisational networks*, *Intra-organisational networks*, *Leadership support*, *Resource*, *Workflow fit*, *Relevance*, *Function, Adaptability*, *Observability*, *External change agent, Peer discussion* Mechanisms: *Compatibility*, *Complexity, Relative advantage, Resource match*
Consolidated framework for implementation research [99]	Variables: *Champion Competing priorities, Extra-organisational networks*, *Intra-organisational networks Leadership support*, *Resource*, *Cost*, *Workflow fit*, *Relevance*, *Function*, *Adaptability*, *Observability, External change agent*, *Peer discussion* Mechanisms: *Complexity*, *Relative advantage*, *Resource match*, *Compatibility*
Multilevel approach to change [96]	Feedback cycle processes: *Behaviour* Variables: *Patient-level* vs *organisation-level*
Feedback theories	
Individual Feedback Theory [48]	Feedback cycle processes: *Feedback*, *Perception*, *Acceptance*, *Intention, Behaviour*, *Clinical performance improvement* Variables: *Controllability*, *Accuracy*, *Patient lists*, *Performance level*, *Specificity*, *Timeliness*, *Function* Mechanisms: *Credibility*
Feedback Intervention Theory [16]	Feedback cycle processes: *Goal setting*, *Feedback, Acceptance*, *Behaviour* Variables: *Benchmarking*, *Performance level*, *Specificity, Trend*, *Active delivery*, *Problem solving*, *Action planning*
Control theory [14]	Feedback cycle processes: *Feedback*, *Perception*, *Acceptance*, *Behaviour*, *Clinical performance improvement* Variables: *Performance level*
General behaviour change theories	
COM-B System [61]	Variables: *Feedback attitude*, *Knowledge and skills in clinical topic*, *Knowledge and skills in quality improvement*, *Importance*, *Performance level*, *Function*, *Ownership*
Motivation-Opportunities-Abilities Model [93]	Variables: *Feedback attitude*, *Knowledge and skills in clinical topic*, *Knowledge and skills in quality improvement*, *Importance*, *Performance level*, *Function*, *Ownership*
Theory of Planned Behaviour [150]	Feedback cycle processes: *Intention*, *Behaviour* Variables: *Feedback attitude*, *Importance*, *Controllability*
Goal setting and action planning theories	
Goal setting theory [15]	Feedback cycle processes: *Goal setting*, *Feedback*, *Behaviour* Variables: *Importance*, *Controllability*, *Performance level*
Guideline adherence theories	
Cabana guideline model [103]	Variables: *Choice alignment*, *Clinical appropriateness*
Guidelines interdependence model [104]	Variables: *Choice alignment*, *Clinical appropriateness*
Motivation theories	
Self-determination theory [60]	Variables: *Intra-organisational networks*, *Teamwork*, *Importance*, *Delivery to a group*, *Function*, *Ownership*, *Peer discussion*
Psychological theories	
Cognitive dissonance [56]	Variables: *Performance level*, *Exclusions*
Cognitive Load Theory [83]	Variables: *Prioritisation*, *Usability*
Self-Affirmation Theory [57]	Variables: *Performance level*
Persuasion theory [78]	Variables: *Champion*, *Intra-organisational networks*, *Leadership support*, *Teamwork*, *Benchmarking*, *Delivery to a group*, *Source knowledge and skill*, *Peer discussion* Mechanisms: *Social influence*
Cognitive fit theory [76]	Variables: *Trend*
Locus of Control [63]	Variables: *Controllability*
Self-Efficacy Theory [109]	Variables: *Controllability*, *Observability*
Obedience to authority [151]	Variables: *Leadership support*, *Source knowledge and skill*
Sociological theories	
Social comparison theory [77]	Variables: *Benchmarking* Mechanisms: *Social influence*
Reference group theory [79]	Variables: *Intra-organisational networks*, *Teamwork*, *Benchmarking*, *Delivery to a group*, *Peer discussion* Mechanisms: *Social influence*
Normative Social Influence [100]	Variables: *Champion*, *Intra-organisational networks*, *Teamwork*, *Delivery to a group*, *Peer discussion* Mechanisms: *Social influence*
Social Learning Theory [106]	Variables: *Extra-organisational networks*, *Training and support*, *External change agent*, *Peer discussion* Mechanisms: *Social influence*
Social Norms Theory [152]	Variables: *Benchmarking* Mechanisms: *Social influence*
Technology theories	
Value chain of information [49]	Feedback cycle processes: *Interaction*
Fit between Individuals, Task, and Technology framework [80]	Variables: *Workflow fit*, *Usability*, *Cost*, *Training and support* Mechanisms: *Compatibility*, *Complexity*
Task-Technology-Fit Model [119]	Variables: *Workflow fit*, *Training and support* Mechanisms: *Compatibility*, *Complexity*
Technology Acceptance Model [66]	Variables: *Feedback attitude*, *Relevance*, *Usability* Mechanisms: *Relative advantage*
Model of Information Systems Success [81]	Variables: *Usability* Mechanisms: *Relative advantage*

*See Additional file 5 for more information

We describe CP-FIT in detail below. To maintain readability, we focus on its high-confidence hypotheses and provide only key example references to supporting studies and theories. CP-FIT’s constructs are in *italics*. Table 8 provides example illustrative quotes and Additional file 5 contains the full descriptions of constructs, with references to supporting papers and theories. Additional file 6 provides case studies demonstrating how CP-FIT can explain the success of different feedback interventions included in the synthesis.

**Table 8:** Example quotes from included papers

Number	Quote	CP-FIT constructs illustrated
1	Physicians’ disagreement with the assessment process results in no action. When they feel performance is based on a small sample of patients that is not representative of the care they provide they ignore the feedback and do not take any action … “The N is incredibly tiny. These patients may not be representative of our typical patient, yet these numbers are taken very seriously.” (Author interpretation and participant quote of a feedback intervention in US primary care [50])	Feedback cycle processes: *Acceptance*, *Behaviour* Variables: *Data collection and analysis method* Mechanisms: *Credibility*
2	Many participants argued that much quality assurance work is being done within the field of diabetes care. As a counterweight, many felt that conditions like hypertension and chronic obstructive pulmonary disease (COPD) were in more need of attention. (Author interpretation of a feedback intervention focusing on diabetes care in Denmark [82])	Feedback cycle processes: *Tunnel vision* Variables: *Importance* Mechanisms: *Credibility*, *Compatibility*
3	All GPs interviewed highly valued the process of reviewing patients identified as receiving high-risk NSAID [non-steroidal anti-inflammatory drug] or antiplatelet prescriptions. “The topic is, I would go so far as to say, essential. I do not even think you can say it’s urgent. It’s essential that practices are doing this. They could be killing patients totally unnecessarily” (Author interpretation and participant quote regarding feedback on potential medication safety errors in Scotland[94])	Feedback cycle processes: *Acceptance*, *Intention* Variables: *Importance* Mechanisms: *Compatibility*
4	The California physicians … [complained] strongly about the accuracy of the data on which their performance was judged... “I have 91 diabetics,” one explained, of whom 32 were reported as “missing either a haemoglobin A1C or an LDL or [to] have elevated levels from September to August ‘07.” But, when he went through the labs and charts, “just on the first two pages I found that six of them were incorrect” (Author interpretation and participant quote regarding feedback in primary care in the US [153])	Feedback cycle processes: *Verification*, *Acceptance* Variables: *Accuracy* Mechanisms: *Credibility*
5	The informants suggested that the identities of the inappropriately treated patients should be revealed in prescriber feedback … “It was frustrating that I had a quality problem without being able to do something about it... (but)... I am not sure whether I actually have a quality problem” (Author interpretation and participant quote regarding feedback on medication prescribing in Denmark [154])	Feedback cycle processes: *Verification*, *Acceptance*, *Perception*, *Intention*, *Behaviour* Variables: *Patient lists* Mechanisms: *Credibility*, *Actionability*
6	Interviewees expressed even greater scepticism about public reporting of performance data … “Sharing [performance data] with [patients] without the opportunity first to improve things might be viewed as punitive.” (Author interpretation and participant quote regarding hospital-based feedback on stroke in the US [155])	Feedback cycle processes: *Acceptance* Variables: *Function* Mechanisms: *Compatibility*
7	No participants reported using the feedback to set specific goals for improvement or action plans for reaching these goals. Even when prompted, most participants could not envision ways for the practice to facilitate pro-active chronic disease management … (Author interpretation of feedback focusing on chronic diseases in Canada [90])	Feedback cycle processes: *Intention*, *Behaviour* Variables: *Knowledge and skills in quality improvement* Mechanisms: *Actionability*, *Resource match*
8	Increased awareness of suboptimal performance usually resulted in the intention to “try harder” to do more during each patient visit, rather than “work smarter” by implementing point-of-care reminders or initiating systems to identify and contact patients for reassessment … Such findings help to explain the small to moderate effects generally observed in randomised trials of audit and feedback. (Author interpretation of feedback intervention focusing on chronic diseases in Canada [90])	Feedback cycle processes: *Intention*, *Behaviour*, *Clinical performance improvement* Variables: *Organisation-level behaviour*, *Patient-level behaviour* Mechanisms: *Actionability*
9	In both interviews and observed meetings, the executive team expressed a deep commitment to ensuring the safety and quality of the services provided by the hospital. Members of the team identified the [feedback system] as a major strategic component of this commitment and made an accordingly heavy investment (approximately UK£25 million or US$38 million over ten years). (Author interpretation of a hospital-based feedback intervention in England [87])	Feedback cycle processes: Nil Variables: *Leadership support*, *Resource* Mechanisms: *Resource match*, *Social influence*
10	That effective surgical site infection [SSI] prevention requires a team effort was a preponderant view … Interprofessional collaboration between clinicians, especially between surgeons and anesthesiologists, was invariably viewed as an integral part of the consistent application of best practices and, ultimately, the successful prevention of SSIs. (Author interpretation of a feedback intervention focusing on the reduction of surgical site infections in Canada [72])	Feedback cycle processes: *Behaviour* (*patient-level*) Variables: *Teamwork*, *Intra-organisational networks* Mechanisms: *Actionability*, *Compatibility*, *Social influence*
11	Most providers (as well as some managers) expressed helplessness in their ability to respond [to feedback], especially when large proportions of the list consisted of challenging patients that, despite best efforts, could not achieve treatment goals …. the link between results and evaluation can be undermined when criteria … do not align with treatment guidelines, the latest evidence, and especially principles of patient-centered care. (Author interpretation of feedback focusing on diabetes treatment in US primary care [58])	Feedback cycle processes: *Intention*, *Behaviour* (*patient-level*), *Clinical performance improvement* Variables: *Choice alignment*, *Clinical appropriateness* Mechanisms: *Actionability*, *Compatibility*, *Complexity*
12	An active and interactive approach was observed in teams A and B, reflected in the planning of regular team meetings for discussions of scores, possible problems and solutions, and appointing a responsible person to take action. This approach was lacking in teams C and D, as confirmed by the surgeon from team D: “We should have looked at the data more often and also discussed the results to discover weaknesses.” (Author interpretation and participant quote regarding feedback on breast cancer surgery in The Netherlands [62])	Feedback cycle processes: *Interaction*, *Perception*, *Intention*, *Behaviour* Variables: *Peer discussion*, *Problem solving*, *Action planning* Mechanisms: *Actionability*, *Compatibility*, *Complexity*, *Social influence*, *Resource match*
13	In Cuba and Bolivia, clinicians saw improvements as a direct result of the audit. Clinicians therefore considered audit a worthwhile activity and found it to be a key motivational factor and facilitator in improving clinical practice. (Author interpretation of feedback targeting tuberculosis diagnosis in South America [97])	Feedback cycle processes: *Interaction*, *Intention* Variables: *Observability* Mechanisms: *Relative advantage*

#### The feedback cycle (research objective 1)

Similar to existing feedback [14, 16, 48], goal setting [15], and information value [49] theories, we found that successful feedback exerts its effects through a series of sequential processes, each of which required a non-trivial work commitment from health professionals (Fig. 3). This started with choosing standards of clinical performance against which care would be measured (*Goal setting*), followed by collection and analysis of clinical performance data (*Data collection and analysis*); communication of the measured clinical performance to health professionals (*Feedback*); reception, comprehension, and acceptance of this by the recipient (*Interaction*, *Perception*, and *Acceptance* respectively); a planned behavioural response based on the feedback (*Intention* and *Behaviour*); and ultimately positive changes to patient care (*Clinical performance improvement*). A further step of *Verification* could occur between *Perception* and *Acceptance* where recipients interrogated the data underlying their feedback (e.g. [50]). The cycle then repeated, usually starting with further *Data collection and analysis*. Feedback interventions became less effective if any of the above processes failed, halting progress round the cycle. For example, if *Data collection* was not conducted (e.g. [51]), or a recipient did not *Accept* the feedback they were given (e.g. [52]; Table 8, quote 1).

In addition to potentially improving clinical performance, we found both positive and negative unintended outcomes associated with feedback. Health care organisations often noted improved record-keeping (e.g. [53]), and recipient knowledge and awareness of the feedback topic (e.g. [54]). However, it could also result in: *Gaming*, where health professionals manipulated clinical data or changed the patient population being measured scrutiny to artificially improve their performance (e.g. [55]), or *Tunnel vision*, where health professionals excessively focused on the feedback topic at the detriment of other clinical areas [56, 57] (Table 8, quote 2).

#### Feedback variables (research objective 2)

We found four feedback variables that influenced progress round the feedback cycle: (1) the goal, (2) data collection and analysis methods, (3) feedback display, and (4) feedback delivery.

##### Goal

This variable refers to the clinical topic and the associated clinical behaviours or patient outcomes measured by the feedback intervention. For example, the proportion of diabetic patients with controlled cholesterol in primary care [58], or whether nutritional advice is provided to nursing home residents [59]. Similar to feedback-specific [15] and general behaviour change theories [60, 61], we found *Acceptance* and *Intention* more likely when feedback measured aspects of care recipients thought were clinically meaningful (*Importance*; Table 8, quote 3). *Acceptance* and *Intention* were also more likely when feedback targeted goals within the control of recipients (*Controllability* e.g. [62]) [48, 63] and that were relevant to their job (*Relevance* e.g. [64]) [65, 66].

##### Data collection and analysis method

When undertaken by feedback recipients themselves (*Conducted by recipients* e.g. [67]) or performed manually (*Automation* e.g. [68]), we found the *Data collection and analysis* process was inhibited, often due to a lack of time or skills. In extreme cases, the *Goal setting* process was re-visited in order to find more suitable methods (e.g. [69]).

We found *Acceptance* was more likely when recipients believed the data collection and analysis process produced a true representation of their clinical performance (*Accuracy*) [48], which often related to the positive predictive value of the feedback (i.e. its ability to correctly highlight areas of clinical performance requiring improvement). If perceived *Accuracy* was low, recipients were also more likely to undertake *Verification* (Table 8, quote 4).

Likewise, *Acceptance* was facilitated when feedback recipients could exception report patients they felt were inappropriate to include in feedback (*Exclusions* e.g. [70]) [56]. Potential reasons for exception reporting are discussed in the “Patient population” section.

##### Feedback display

We found *Intention* and *Behaviour* were more likely when feedback communicated recipients’ performance level had room for improvement (*Performance level*). This violated their perception they delivered high-quality care, thus providing motivation and opportunity to change (e.g. [64]) [16, 56, 61]. It also encouraged *Verification* as recipients often wanted to clarify this alternative view of their achievements themselves (e.g. [50]). We found some support for theories that suggested the feedback process could be inhibited if performance was so extreme that improvement was unlikely: [16, 56] for example, non-*Acceptance* if current performance was too low (e.g. [71]), or *Goal setting* re-visited if performance too high (e.g. [53]); though these findings were inconsistent**.**

Feedback that detailed the patients included in the clinical performance calculation (*Patient lists*) facilitated *Verification*, *Perception*, *Intention*, and *Behaviour* by enabling recipients to understand how suboptimal care may have occurred, helping them take corrective action (where possible) for those patients and learn lessons for the future (e.g. [50]). It also facilitated *Acceptance* by increasing transparency and trustworthiness of the feedback methodology [48] (Table 8, quote 5).

Feedback focusing on the performance of individual health professionals rather than their wider team or organisation increased *Acceptance*, *Intention*, and *Behaviour* because, similar to *Controllability* and *Relevance* (see “Goal” section), it was more likely to highlight situations for which they had responsibility (*Specificity* e.g. [72]) [48]. Using recent data to calculate recipients’ current performance (*Timeliness*) had a similar effect because it was based on what recipients could change currently, rather than events that had long passed (e.g. [50]).

Feedback often compared recipients’ current performance to other scores, such as their past performance (*Trend* e.g. [73]), others’ performance (*Benchmarking* e.g. [74]), or an expected standard (usually determined by experts; *Target* e.g. [75]). We found that *Trend* facilitated *Perception* by helping recipients interpret their current performance in a historical context [16, 76]. *Benchmarking* worked in a similar fashion by helping recipients understand how they performed relative to other health professionals or organisations, stimulating *Intention* and *Behaviour* because they wanted to do better than their colleagues and neighbours [77]. *Benchmarking* also worked by motivating recipients to maintain their social status when they saw others in their peer group behaving differently [78, 79]. These findings contradicted Feedback Intervention Theory, which predicts that drawing attention to other’s performance reduces the impact of feedback [16]. It was unclear whether *Benchmarking* was more effective when the identities of the health professionals were visible to each other, or to which health professionals’ performance should be compared. We found only minimal evidence that *Targets* influenced feedback effectiveness despite their prominence in existing feedback theories [14–16].

Feedback was more effective when it communicated the relative importance of its contents (*Prioritisation*) and employed user-friendly designs (*Usability*) [80, 81], because it reduced cognitive load by helping recipients decide what aspects of their performance required attention (e.g. [55, 82]) [83]. Studies provided little detail on how this could be practically achieved, though strategies may include limiting the number of clinical topics in the feedback (*Number of metrics* e.g. [55]) or using charts (*Graphical elements* e.g. [84]) [76]. We found insufficient evidence that feedback’s effectiveness was influenced by whether it was presented positively or negatively (*Framing*) [16, 48].

##### Feedback delivery

Recipients often rejected feedback whose purpose they believed was to punish rather than support positive change because it did not align with their inherent motivation to improve care (*Function* e.g. [85]) [60, 61]. Similarly, when feedback was reported to external organisations or the public, it often drew negative reactions with little evidence of impact on clinical performance (*External reporting* e.g. anxiety and anger [75]; Table 8, quote 6).

*Acceptance* was also less likely when delivered by a person or organisation perceived to have an inappropriate level of knowledge or skill (*Source knowledge and skill*). This could relate to the clinical topic on which feedback was provided (e.g. [86]) or quality improvement methodology (e.g. [85]) [48]. We found inconsistent evidence that the location of feedback delivery, for example whether internal or external to the recipients’ organisation, influenced effectiveness (*Source location*).

Feedback that was “pushed” to recipients facilitated *Interaction* more than those requiring them to “pull” it (*Active delivery*). For example, feedback sent by email (e.g. [87]) was received more frequently than when published in a document that was not distributed (e.g.[75]). An exception was feedback solely delivered in face-to-face meetings, as the significant time commitments often meant health professionals could not attend (e.g. [88]).

Feedback delivered to groups of health professionals improved *Teamwork* (see “Organisation or team characteristics” section) by promoting engagement and facilitating discussion (*Delivery to a group* e.g. [89]) [60, 78]. There was inconsistent evidence on the effects of how often feedback was delivered (*Frequency*) [5], and little insight into whether it was best delivered electronically or on paper (*Medium*) [5, 16].

#### Recipient variables (research objective 2)

We found two recipient variables that influenced progress round the feedback cycle: (1) health professional characteristics and (2) their behavioural response.

##### Health professional characteristics

Often health professionals did not possess the knowledge and skills to effectively engage with and respond to feedback. This included technical quality improvement skills such as interpreting data or formulating and implementing action plans, rather than the clinical topic in question (e.g. [90]). We found interventions targeting those with greater capability (both technical and clinical) were more effective because recipients were more likely to successfully proceed through the *Perception*, *Intention*, and *Behaviour* feedback processes (*Knowledge and skills in quality improvement* and the *clinical topic*, respectively; e.g. [91, 92]) [61, 93]. This seemed to undermine the rationale of interventions predicated on addressing health professionals’ presumed lack of clinical knowledge (e.g. [94]; Table 8, quote 7).

Understandably, health professionals with positive views on the potential benefits of feedback were more likely to engage with it (*Feedback attitude* e.g. [64]) [66, 93]. And although health professionals often had profound emotional reactions to feedback, both positive and negative (e.g. [85]), we found no reliable evidence that these directly influenced the feedback cycle.

##### Behavioural response

We found two main types of action taken by recipients (if any) in response to feedback: those relating to the care of individual patients one-at-a-time (*Patient-level*) or those aimed at the wider health care system (*Organisation-level*). *Patient-level* behaviours included retrospectively “correcting” suboptimal care given in the past, or prospectively providing “better” care to patients in the future. For example, resolving medication safety errors by withdrawing previously prescribed medications [86] versus optimising treatment when a patient with uncontrolled diabetes is next encountered [90]. In contrast, *Organisation-level* behaviours focused on changing care delivery systems. For example, changing how medications are stored in hospital [87], or introducing computerised decision support software to support clinician-patient interactions [95]. We found *Organisation-level* behaviours often led to greater *Clinical performance improvement* because they enabled multiple *Patient-level* behaviours by augmenting the clinical environment in which they occurred [96]. For example, changing how medications were stored reduced the likelihood of delayed administration to all patients [87], and decision support software could remind clinicians how to optimally treat diabetic patients [95]. Conversely, by definition, *Patient-level* behaviours only ever affected one patient (Table 8, quote 8). We found no clear evidence that feedback success was affected if it required an increase, decrease, change, or maintenance of recipients’ current clinical behaviours to improve their performance (*Direction*) [5, 7].

#### Context variables (research objective 2)

We found three context variables that influenced progress round the feedback cycle: (1) organisation or team characteristics, (2) patient population, (3) co-interventions, and (4) implementation process.

##### Organisation or team characteristics

We found all organisations and teams had a finite supply of staff, time, finances, and equipment (e.g. [90]), stretched by the complexity of modern health care, such as serving increasing numbers of elderly multimorbid patients and dealing with wider organisational activities such as existing quality improvement initiatives and re-structures (e.g. [97]). Consequently, if an organisation had less capacity (*Resource*) or significant other responsibilities (*Competing priorities*), they were less able to interact with and respond to feedback (e.g. [90, 98]) [65, 99]. However, if senior managers advocated for the feedback intervention or individuals were present who were dedicated to ensuring it was a success, they often influenced others and provided additional resource to enable more meaningful engagement with feedback (*Leadership support* e.g. [87] and *Champions* e.g. [68], respectively; Table 8, quote 9) [78, 100].

Increased *Resource* and *Leadership support* also increased the likelihood that *Organisation-level* behaviours were undertaken (see “Behavioural response” section), because they often required process redesign and change management (e.g. [82]). In turn, *Organisation-level* behaviours also had the potential to further increase *Resource*, for example by recruiting new staff (e.g. [101]) or purchasing new equipment (e.g.[75]), which in turn further increased their capacity to engage with and respond to feedback.

Feedback was more successful when members of organisations and teams worked effectively towards a common goal (*Teamwork*; e.g. [72]), had strong internal communication channels (*Intra-organisational networks*; e.g. [51]), and actively communicated with other organisations and teams (*Extra-organisational networks*; e.g. [86]) [65, 99]. These characteristics often co-existed and provided practical support for feedback recipients during *Interaction*, *Perception*, *Intention*, and *Behaviour* (Table 8, quote 10).

Organisations and teams also commonly had long-established systems and processes that were often difficult to change, such as methods of care delivery and technical infrastructure (e.g. [102]). Therefore, if the feedback intervention fitted alongside their existing ways of working (*Workflow fit*) [65, 80], it required less effort to implement (e.g. [64]).

##### Patient population

Health professionals felt it was inappropriate to include certain patients in the their clinical performance calculation [103, 104]. For example, patients that refused the aspects of care measured by the feedback (*Choice alignment*; e.g. [105]), or those who already received maximal therapy or had relevant clinical contraindications (e.g. medication allergies; *Clinical appropriateness*; Table 8, quote 11). Including such patients in their clinical performance calculation inhibited *Acceptance* and *Intention*, with some evidence it may have also led to *Gaming* ( “The feedback cycle (research objective 1)” section e.g. [101]).

##### Co-interventions

Synthesised papers used eight different quality improvement interventions alongside feedback (Table 4). However, only four appeared to impact feedback success because they addressed specific barriers. The provision of support for health professionals to discuss their feedback with peers (*Peer discussion*) and to identify reasons for and develop solutions to suboptimal performance (*Problem solving* and *Action planning*) facilitated *Perception*, *Intention*, and *Behaviour*. These co-interventions addressed shortcomings in health professionals’ quality improvement skills (see “Health professional characteristics” section). *Peer discussion* had the added benefit of improving *Teamwork* (see “Organisation or team characteristics” section) [60]. Such approaches often co-existed, and could be delivered in different ways, for example as didactic workshops (e.g. [89]) or led by recipients themselves (e.g. [90]), though it was unclear which was most effective (Table 8, quote 12).

Co-interventions that provided additional staff to explicitly support the implementation of feedback helped overcome time and staffing issues (see “Organisation or team characteristics” section; *External change agents*) [65, 99]. These personnel could either be directly involved in feedback processes (e.g. carrying out improvement actions [86]), or indirectly support recipients (e.g. facilitating *Perception* and *Intention* [94]) [106].

We found little support for education (*Clinical education*) or financial incentives (*Financial rewards*). There was some evidence that *Financial rewards* could negatively impact feedback success by conflicting with recipients’ motivation and sense of professionalism (e.g. [107]) [60, 61].

##### Implementation process

How feedback was introduced into clinical practice impacted all feedback cycle processes. Feedback tailored to the specific requirements of the health care organisation and its staff appeared more successful because it aligned with their needs and improved *Workflow fit* (see “Organisation or team characteristics” section; *Adaptability*) [65, 99]. For example, if quality indicator definitions could be amended to fit existing data sources [69] or focus on local clinical problems [91].

When training and support were provided on how to use an intervention (not the clinical topic under scrutiny; *Training and support*), it improved recipients’ *Knowledge and skills in quality improvement* (see “Health professional characteristics” section; e.g. [91]) [80, 106]. Further, if the training demonstrated the intervention’s potential benefits (*Observability*), recipients were also more likely to engage with it [65, 108]. These benefits could be to recipients themselves, such as improved feedback user-friendliness (*Usability*, the “Feedback display” section; e.g. [98]), or to patient care (e.g. [88]). *Trend* (see “Feedback display” section) could also increase *Observability* if its trajectory was positive (Table 8, quote 13) [109].

Interventions considered “expensive” to deploy, in terms of time, human, or financial resources, were generally less effective because they required more resource or effort (*Cost*) [80, 99]. Examples of expensive interventions included when data collection was *Conducted by recipients* (see “Data collection and analysis method” section; e.g. [67]) or when feedback was delivered solely face-to-face (see “Feedback delivery” section; e.g. [88]).

We found more consistent evidence to support interventions that made recipients feel like they “owned” the feedback intervention rather than those imposed via external policies or directives [65, 99] because they harnessed their autonomy and internal motivation to improve patient care (*Ownership*) [60, 61]. Despite this, we found little support for seeking input from recipients into the design and implementation of feedback (*Linkage at the development stage*) [65].

#### Mechanisms (research objective 3)

We found seven explanatory mechanisms through which the above variables operated. Many mirrored constructs from existing theories of context and implementation [65, 99, 108], and variables often effected change through multiple mechanisms (Table 5).

##### Complexity

*Complexity* is how straightforward it was to undertake each feedback cycle process. This could refer to the number of steps required or how difficult they were to complete. Simple feedback facilitated all feedback cycle processes.

##### Relative advantage

*Relative advantage* refers to whether health professionals believed the feedback had a perceived advantage over alternative ways of working, including other feedback interventions. Understandably, variables operating via this mechanism depended on the specific circumstances into which they were implemented. *Relative advantage* facilitated all feedback cycle processes.

##### Resource match

*Resource match* details whether health professionals, organisations, and teams had adequate resources to engage with and respond to those required by the feedback intervention. It included time, staff capacity and skills, equipment, physical space, and finances. When *Resource match* was achieved, all feedback cycle processes were facilitated.

##### Compatibility

*Compatibility* characterises the degree to which the feedback interventions aligned with the beliefs, values, needs, systems, and processes of the health care organisations and their staff. *Compatibility* facilitated all feedback cycle processes.

##### Credibility

*Credibility* was how health professionals perceived the trustworthiness and reliability of the feedback. Recipients were more likely to believe and engage with credible feedback [48], which facilitated *Interaction*, *Verification*, *Acceptance*, *Intention*, and *Behaviour*.

##### Social influence

*Social influence* specifies how much the feedback harnessed the social dynamics of health care organisations and teams. Exploiting *Social influence* could facilitate all feedback cycle processes.

##### Actionability

*Actionability* describes how easily health professionals could take action in response to feedback and in turn how directly that action influenced patient care. *Actionability* facilitated *Intention*, *Behaviour*, and *Clinical performance improvement*.

#### Propositions (research objective 4)

We distilled the above hypotheses of how context and intervention variables influenced feedback cycle processes (Table 5) into three propositions that govern the effects of feedback interventions (Table 6). Each proposition summarised multiple variable hypotheses, though only related to a mutually exclusive set of explanatory mechanisms.

## Discussion

### Summary of findings

CP-FIT describes causal pathways of feedback effectiveness synthesised from 65 qualitative studies of 73 interventions (Table 4), and 30 pre-existing theories (Table 7). It states that effective feedback is a cyclical process of *Goal setting*, *Data collection and analysis*, *Feedback*, recipient *Interaction*, *Perception*, and *Acceptance* of the feedback, followed by *Intention*, *Behaviour*, and *Clinical performance improvement* (the feedback cycle; Fig. 3). Feedback becomes less effective if any individual process fails causing progress round the cycle to stop and is influenced by variables relating to the feedback itself (its *Goal*, *Data collection and analysis methods*, *Feedback display*, and *Feedback delivery*), the recipient (*Health professional characteristics*, and *Behavioural response*), and context (*Organisation or team characteristics*, *Patient population*, *Co-interventions* and *Implementation process*). These variables exert their effects via explanatory mechanisms of *Complexity*, *Relative advantage*, *Resource match*, *Compatibility*, *Credibility*, *Social influence*, and *Actionability* (Table 5) and are summarised by three propositions (Table 6).

### Applying CP-FIT in practice and research

Each of Table 5’s 42 high-confidence hypotheses can be viewed as specific design recommendations to increase feedback effectiveness. For example, hypothesis 12 (*Trend*) recommends feedback should display recipients’ current performance in relation to their past performance; hypothesis 17 (*Source knowledge and skill*) recommends feedback should be delivered by a person or organisation perceived as having an appropriate level of knowledge or skill by recipients; and hypothesis 26 (*Leadership support*) recommends that feedback interventions should seek the support of senior managers in health care organisations when implemented. For practitioners and policy-makers, CP-FIT therefore provides guidance they should consider when developing and deploying feedback interventions. This includes national clinical audits (e.g. [110, 111]), pay-for-performance programmes (e.g. [112, 113]), and learning health systems (where routinely collected health care data is analysed to drive continuous improvement [114])—such programmes are large-scale, address impactful clinical problems (e.g. cardiovascular mortality or antimicrobial resistance) [9, 10], and require substantial expenditure to develop and maintain (e.g. data collection and analysis infrastructure) [4, 115]. Using CP-FIT thus has the potential to improve care for large numbers of patients, in addition to reducing the opportunity cost from unsuccessful feedback initiatives and improving returns on health care systems’ financial investments.

Table 5’s hypotheses can also be translated into explanations why feedback may or may not have been effective. Additional file 6 provides examples of how to do this by presenting three case studies of different feedback interventions included in our meta-synthesis [74, 86, 116], and using CP-FIT to explain their successes and failures. CP-FIT can therefore help researchers and feedback evaluators assess and explain feedback’s observed or predicted effects. Specifically for qualitative methodologists, Additional file 5 provides a comprehensive codebook that can be used to analyse data and discover causal pathways. For quantitative investigators, both Table 5 and Additional file 5 provide over 200 potentially falsifiable hypotheses to test. As illustrated in Additional file 6, CP-FIT may be particularly useful in process evaluations to identify weak points in a feedback interventions’ logic model (i.e. the feedback cycle; Fig. 3) [17, 117] and barriers and facilitators to its use (i.e. its variables) [11].

Although developed specifically for feedback, CP-FIT may also have relevance to other quality improvement strategies that analyse patient data and communicate those analyses to health professionals in order to effect change. Examples include computerised clinical decision support and educational outreach [118], where CP-FIT concepts such as *Accuracy* (see “Data collection and analysis method” section), *Timeliness* (see “Feedback display” section), *Credibility* (see “Credibility” section), and *Actionability* (see “Actionability” section) may all be important. CP-FIT concepts related to population-level feedback (e.g. *Benchmarking* and *Trend*; the “Feedback display” section) may be less relevant when the focus of the intervention is on individual patient-level care, such as in clinical decision support [18].

### Comparison to existing literature

Table 9 shows how CP-FIT may explain reasons for feedback effectiveness variation found in the latest Cochrane review [8]. CP-FIT suggests further sources of variation not identified that could be operationalised in a future update of the review: for example, if feedback allows *Exclusions* or provides *Patient lists* (see “Data collection and analysis method” and “Feedback display” sections, respectively).

**Table 9:** How CP-FIT may explain findings from the Cochrane review

Cochrane review finding: Feedback may be most effective when …	Potential explanation according to CP-FIT
… The health professionals are not performing well to start out with.	Low *Performance level* facilitates *Intention* and *Behaviour* because it increases *Compatibility* with recipients’ personal views (i.e. that they want to provide high quality patient care) and *Actionability* (i.e. low performance implies room for improvement).
… The person responsible for the audit and feedback is a supervisor or colleague.	A supervisor or colleague is likely to be perceived to have greater knowledge and skill (*Source—knowledge and* skill), which facilitates *Acceptance* by increasing *Credibility*.
… It is provided more than once.	Multiple instances of feedback are inherent to the feedback cycle (Fig. 3).
… It is given both verbally and in writing.	Feedback that is actively “pushed” to recipients i.e. verbally (*Active delivery*) facilitates *Interaction* by reducing *Complexity* by ensuring the feedback received. However, solely providing feedback face-to-face (verbally) inhibits *Interaction* by decreasing *Resource match* as it requires significant time commitment from recipients, so is enhanced if also provided in other ways.
… It includes clear targets and an action plan.	“Targets” in the Cochrane review equated to *Benchmarking* and *Trend*, both of which facilitate *Perception*, *Intention*, *Behaviour* by decreasing *Complexity* (making it easier for recipients to know what constitutes “good performance” and therefore what requires a corrective response) and increasing *Social influence* (stimulating recipients’ sense of competition). *Action planning* and *Problem solving* facilitate *Intention* and *Behaviour* by increasing *Actionability* (providing practical support on how to respond effectively to the feedback message) and *Resource match* (by addressing health professionals’ general lack of knowledge and skills to perform these behaviours).

CP-FIT aligns well with tentative best practices for effective feedback interventions [5, 11] and provides potential evidence-based explanations as to why they may work (Table 10). It also provides additional potential recommendations such as automating data collection and analysis (*Automation*; see “Data collection and analysis method” section) and gaining leadership support (*Leadership support*; see “Organisation or team characteristics” section). An advantage of CP-FIT over these existing best practice recommendations is that it provides parsimonious generalisable principles (in the form of its explanatory mechanisms and propositions; see “Mechanisms (research objective 3)” and “Propositions (research objective 4)” sections, respectively). Consequently, CP-FIT’s hypotheses can be extended beyond those in Table 5 if they conform to these constructs. For example, the *Complexity* (see “Complexity” section) of a feedback interventions’ targeted clinical behaviour (*Goal*; see “Goal” section) may be reasonably expected to influence its effectiveness [80, 119], despite not being a consistent finding in our synthesis.

**Table 10:** Tentative best practices for feedback interventions compared to CP-FIT

Brehaut et al. [11]	Ivers et al. [5]	CP-FIT variables
Address credibility of the information.	Data are valid	*Accuracy* *Source—knowledge and skill* *Function*
	Delivery comes from a trusted source	*Source—knowledge and skill*
Provide feedback as soon as possible and at a frequency informed by the number of new patient cases	Data are based on recent performance	*Timeliness*
Provide individual rather than general data.	Data are about the individual/team’s own behaviour(s)	*Specificity*
Provide multiple instances of feedback.	Audit cycles are repeated, with new data presented over time	Multiple instances of feedback are inherent to the feedback cycle (Fig. 3).
Provide feedback in more than 1 way.	Presentation is multi-modal including either text and talking or text and graphical materials	*Active delivery*
Choose comparators that reinforce desired behaviour change	The target performance is provided	*Benchmarking* *Trend*
	Feedback includes comparison data with relevant others	
Recommend actions that can improve and are under the recipient’s control.	Targeted behaviour is likely to be amenable to feedback	*Controllability* *Performance level*
	Recipients are capable and responsible for improvement	
Recommend actions that are consistent with established goals and priorities	Goals set for the target behaviour are aligned with personal and organisational priorities	*Importance* *Relevance* *Workflow alignment*
Recommend specific actions	Goals for target behaviour are specific, measurable, achievable, relevant, time-bound	*Action planning* *Problem solving* *Peer discussion*
	A clear action plan is provided when discrepancies are evident	
Closely link the visual display and summary message	N/A	*Usability*
Minimise extraneous cognitive load for feed- back recipients.	N/A	*Prioritisation* *Usability*
Provide short, actionable messages followed by optional detail.	N/A	*Patient lists* *Prioritisation*
Address barriers to feedback use.	N/A	CP-FIT in its entirety can be used to address barriers
Prevent defensive reactions to feedback.	N/A	*Function*
Construct feedback through social interaction.	N/A	*Peer discussion*

Table 7 demonstrates how pre-existing theories contribute to, and overlap with, CP-FIT. In comparison to other theories used to model clinical performance feedback [14–16, 48], CP-FIT adds value for health care settings by specifying potential unintended consequences (see “The feedback cycle (research objective 1)” section); detailing new context-related constructs, for example in relation to the organisation or team (see “Organisation or team characteristics” section); and elaborating on specific aspects of the feedback process, for example data collection and analysis (see “Data collection and analysis method” section). This wider and more detailed view may explain why CP-FIT occasionally provides different predictions: [14, 16, 48] for example, Feedback Intervention Theory predicts the presentation of others’ performance (*Normative information*) decreases effectiveness by diverting attention away from the task at hand [16], whereas CP-FIT states it does the opposite by harnessing the social dynamics between recipients (*Benchmarking*; see “Data collection and analysis method” section).

To our knowledge, a systematic search and synthesis of qualitative evaluations of feedback interventions has not been previously undertaken. However, two reviews exploring the use of patient-reported outcome measure (PROM) feedback in improving patient care have been recently published [120, 121]. Although neither explicitly attempted to develop theory, their main findings can be mapped to CP-FIT constructs. Boyce et al. [120] found there were practical difficulties in collecting and managing PROMs data related to an organisation’s resources and compatibility with existing workflows (cf. CP-FIT Propositions 1 and 2, respectively; Table 6); whereas Greenhalgh et al. [121] note “actionability” as a key characteristic in the effective use of PROM data (cf. CP-FIT Proposition 3; Table 6). Both noted the “credibility” of data and source from which it was fed back were essential to securing health professional’s acceptance (cf. CP-FIT’s *Credibility*; see “Credibility” section).

Colquhoun et al. generated 313 theory-informed hypotheses about feedback interventions by interviewing subject experts [122]. Many of the hypotheses appear in CP-FIT (e.g. feedback will be more effective if patient-specific information is provided cf. CP-FIT’s *Patient lists*; see “Data collection and analysis method” section), though some are contradictory (e.g. feedback will be less effective when presented to those with greater expertise cf. CP-FIT’s *Knowledge and skills in quality improvement* and *clinical topic*; see “Health professional characteristics” section) [122]. A possible explanation is that Colquhoun et al.’s hypotheses have been informed by disparate research paradigms (including those outside health care) rather than attempting to develop a unifying theory based on empirical evaluations of feedback interventions like CP-FIT. Work is ongoing to prioritise these hypotheses for empirical testing [122], which will also help further validate CP-FIT.

### Limitations

Like all literature syntheses, our findings reflect only what has been reported by its constituent studies. Consequently, CP-FIT may not include features of feedback interventions or contexts associated with effectiveness that have been under-reported. This may manifest by such findings being absent, having “low” or “moderate” GRADE-CERQual ratings (Additional file 5) or unclear effects (e.g. *Frequency*; see “Feedback delivery” section or *Direction*, see “Behavioural response” section). For similar reasons CP-FIT’s current form may also lack detail regarding certain construct definitions (e.g. how is good *Usability* [see “Feedback display” section] or effective *Action planning* [see “Co-interventions” section] best achieved?), how particular variables may be manipulated in practice (e.g. how can we persuade health professionals of a feedback topic’s *Importance* [see “Goal” section] or to undertake *Organisation-level* as well as *Patient-level* behaviour [see “Behavioural response” section]?), and inherent tensions within the theory (e.g. how do we ensure *Compatibility* [see “Compatibility” section] whilst also attempting to change health professional behaviour?). Future research should address these evidence gaps by evaluating innovative new feedback designs delivered in different contexts, employing both robust qualitative and quantitative approaches, using CP-FIT as a framework.

Finally, CP-FIT does not currently quantify the relative effect sizes of its variables and mechanisms. It is possible that variables appearing to influence feedback effectiveness with “high” GRADE-CERQual confidence may in fact have negligible effects on patient care. Consequently, future work should aim to quantitatively test CP-FIT’s hypotheses and refine its assumptions.

## Conclusions

The advent of electronic health records and web-based technologies has resulted in widespread use and expenditure on feedback interventions [4, 115]. Whilst there is pressure to provide higher quality with lower costs, the messy reality of health care means feedback initiatives have varying success [8]. This results in missed opportunities to improve care for large populations of patients (e.g. [9, 10]) and see returns on financial investments. Feedback interventions are often as complex as the health care environments in which they operate, with multiple opportunities and reasons for failure (Fig. 3 and Table 5). To address these challenges, we have presented the first reported qualitative meta-synthesis of real-world feedback interventions and used the results to develop the first comprehensive theory of feedback designed specifically for health care. CP-FIT contributes new knowledge on how feedback works in practice (research objective 1) and factors that influence its effects (research objectives 2 and 3, respectively), in a parsimonious and usable way (research objective 4). CP-FIT meets the definition of a theory provided in the “Background” section [13] because it (1) coherently describes the processes of clinical performance feedback (see “The feedback cycle (research objective 1)” section and Fig. 3), (2) was arrived at by inferring causal pathways of effectiveness and ineffectiveness from 65 studies of 73 feedback interventions (as detailed in Additional file 3), (3) can provide explanations as to why feedback interventions were effective or ineffective (as demonstrated by the case studies in Additional file 6), and (4) generates predictions about what factors make feedback interventions more or less effective (see hypotheses in Table 4 and Additional file 5). We hope our findings can help feedback designers and practitioners build more effective interventions, in addition to supporting evaluators discern why a particular initiative may (not) have been successful. We strongly encourage further research to test CP-FIT whilst exploring its applicability to other quality improvement strategies, refining and extending it where appropriate.

Contributions to the literature

- Providing quantitative summaries of clinical performance when treating specific groups of patients (“feedback”) is a widely used quality improvement strategy, yet it has varying success.
- Theory could help explain what factors influence feedback success; however, existing theories lack detail and specificity to health care.
- This is the first systematic review and meta-synthesis of qualitative evaluations of feedback interventions and presents the first comprehensive health care-specific feedback theory that can be used to design, implement, and evaluate feedback (Clinical Performance Feedback Intervention Theory; CP-FIT).
- Using CP-FIT could help improve care for large numbers of patients, reduce opportunity costs from unsuccessful interventions, and improve returns on feedback infrastructure investment.

## Additional files

Additional file 1: Search terms. (DOCX 118 kb)

Additional file 2: Example data extraction form. (DOCX 61 kb)

Additional file 3: Data synthesis method. (DOCX 171 kb)

Additional file 4: Study details. (DOCX 155 kb)

Additional file 5: CP-FIT codebook. (DOCX 248 kb)

Additional file 6: CP-FIT case studies. (DOCX 933 kb)

## Abbreviations

CP-FIT: Clinical Performance Feedback Intervention Theory
PROM: Patient-reported outcome measure
PROSPERO: Prospective Register of Systematic Reviews
