# Supplementary material for: Clinical Performance Feedback Intervention Theory (CP-FIT): a new theory for designing, implementing, and evaluating feedback in health care based on a systematic review and meta-synthesis of qualitative research
Source: Implement Sci. 2019 Apr 26;14:40. doi: 10.1186/s13012-019-0883-5 (PMC6486695; doi:10.1186/s13012-019-0883-5)
Supplement: Supplementary file 1 — Search terms. (DOCX 118 kb) [file 13012_2019_883_MOESM1_ESM.docx]

# Additional file 1: Search terms

**MEDLINE (Ovid) 1946 to January Week 3 2015 – searched 25th March 2015**

| 1 | (audit* adj3 feedback).tw. | 1676 |
| --- | --- | --- |
| 2 | Clinical Audit/ | 833 |
| 3 | Medical Audit/ | 14940 |
| 4 | Nursing Audit/ | 2953 |
| 5 | Dental Audit/ | 370 |
| 6 | Management Audit/ | 2394 |
| 7 | Benchmarking/ | 10374 |
| 8 | "Commission on Professional and Hospital Activities"/ | 229 |
| 9 | Feedback/ | 26135 |
| 10 | Feedback, Psychological/ | 2302 |
| 11 | Utilization Review/ | 7145 |
| 12 | Drug Utilization Review/ | 3078 |
| 13 | Concurrent Review/ | 376 |
| 14 | Peer Review, Health Care/ | 1306 |
| 15 | (audit or audits or auditing).tw. | 24805 |
| 16 | feedback.tw. | 75736 |
| 17 | (review adj3 record?).tw. | 9996 |
| 18 | chart review.tw. | 20512 |
| 19 | (practice data or hospital* data).tw. | 3244 |
| 20 | benchmark*.tw. | 15495 |
| 21 | or/2-20 | 186397 |
| 22 | exp Health Personnel/ | 376175 |
| 23 | exp Hospitals/ | 206858 |
| 24 | exp Professional Practice/ | 219267 |
| 25 | Family Practice/ | 59981 |
| 26 | Professional Competence/ | 20499 |
| 27 | Clinical Competence/ | 66711 |
| 28 | Physician's Practice Patterns/ | 42310 |
| 29 | Nurse's Practice Patterns/ | 1068 |
| 30 | Dentist's Practice Patterns/ | 1702 |
| 31 | Quality Assurance, Health Care/ | 49537 |
| 32 | Quality of Health Care/ | 58038 |
| 33 | ((health* personnel or health care personnel or physician? or doctor? or clinician? or nurse? or provider? or practitioner? or resident? or professional? or nursing or clinical) adj3 (skill or skills or behavior or behavior or competence)).tw. | 27615 |
| 34 | ((clinical or medical or dental or private or general or family or professional or hospital?) adj practice?).tw. | 164564 |
| 35 | (practice pattern? or pattern of practice).tw. | 4627 |
| 36 | (quality adj (assurance or improvement or control)).tw. | 56964 |
| 37 | (healthcare quality orhealthcare quality orquality ofhealthcare or quality of health care or quality of care).tw. | 31494 |
| 38 | Performance.tw. | 487430 |
| 39 | ((influenc* or chang*) adj3 (behavior* or behavior*)).tw. | 47229 |
| 40 | or/22-39 | 1579525 |
| 41 | 21 and 40 | 56737 |
| 42 | 1 or 41 | 57748 |
| 43 | ((("semi-structured" or semistructured or unstructured or informal or "in-depth" or indepth or "face-to-face" or structured or guide) adj3 (interview* or discussion* or questionnaire*)) or (focus group* or qualitative or ethnograph* or fieldwork or "field work" or "key informant")).ti,ab. or interviews as topic/ or focus groups/ or narration/ or qualitative research/ | 210149 |
| 44 | 42 and 43 | 3520 |
| 45 | Animals/ | 5358571 |
| 46 | Humans/ | 13628325 |
| 47 | 45 not (45 and 46) | 3879559 |
| **48** | **44 not 47** | **3510** |

**EMBASE (Ovid) 1980 to 2015 Week 04 – searched 25th March 2015**

| 1 | (audit* adj3 feedback).tw. | 2130 |
| --- | --- | --- |
| 2 | Medical Audit/ | 38426 |
| 3 | Feedback System/ | 59035 |
| 4 | Negative Feedback/ | 11073 |
| 5 | Positive Feedback/ | 6141 |
| 6 | "utilization review"/ | 65402 |
| 7 | "medical record review"/ | 60421 |
| 8 | (audit or audits or auditing).tw. | 46609 |
| 9 | feedback.tw. | 98828 |
| 10 | (review adj3 record?).tw. | 15290 |
| 11 | chart review.tw. | 38408 |
| 12 | (practice data or hospital* data).tw. | 5465 |
| 13 | benchmark*.tw. | 22442 |
| 14 | or/2-13 | 352975 |
| 15 | exp Health Care Personnel/ | 961050 |
| 16 | exp Hospital/ | 719419 |
| 17 | exp Professional Practice/ | 271288 |
| 18 | Professional Competence/ | 24700 |
| 19 | Nursing Competence/ | 544 |
| 20 | Clinical Competence/ | 43524 |
| 21 | Health Care Quality/ | 189463 |
| 22 | Quality Control/ | 123686 |
| 23 | ((health* personnel or health care personnel or physician? or doctor? or clinician? or nurse? or provider? or practitioner? or resident? or professional? or nursing or clinical) adj3 (skill or skills or behavior or behavior or competence)).tw. | 36116 |
| 24 | ((clinical or medical or dental or private or general or family or professional or hospital?) adj practice?).tw. | 228866 |
| 25 | (practice pattern? or pattern of practice).tw. | 6822 |
| 26 | (quality adj (assurance or improvement or control)).tw. | 85444 |
| 27 | (healthcare quality orhealthcare quality orquality ofhealthcare or quality of health care or quality of care).tw. | 43079 |
| 28 | performance.tw. | 654258 |
| 29 | ((influenc* or chang*) adj3 (behavior* or behavior*)).tw. | 61638 |
| 30 | or/15-29 | 2726571 |
| 31 | 14 and 30 | 135799 |
| 32 | 1 or 31 | 136885 |
| 33 | limit 32 to exclude medline journals | 8788 |
| 34 | (interview: or qualitative).tw. or exp health care organization/ | 1467514 |
| 35 | 33 and 34 | 2800 |
| 36 | Nonhuman/ | 4432890 |
| **37** | **35 not 36** | **2775** |

**CINAHL Plus (Ebsco) 1937 to present – searched 25th March 2015**

| S42 | S40 AND S41 | 2,080 |
| --- | --- | --- |
| S41 | ((MH “study design+” not MM “study design+”) or MH “attitude” or (MH “interviews+” not MM “interviews+”)) | 801,586 |
| S40 | S39 (Limiters - Exclude MEDLINE records) | 8,029 |
| S39 | S37 or S38 | 24,058 |
| S38 | S13 and S36 | 23,681 |
| S37 | TI ( audit* and feedback ) or AB ( audit* and feedback ) | 882 |
| S36 | S14 or S15 or S16 or S17 or S18 or S19 or S20 or S21 or S22 or S23 or S24 or S25 or S26 or S27 or S28 or S29 or S30 or S31 or S32 or S33 or S34 or S35 | 763,238 |
| S35 | TI ( influenc* N3 behavior* or influenc* N3 behavior* or chang* N3 behavior* or chang* N3 behavior* ) or AB ( influenc* N3 behavior* or influenc* N3 behavior* or chang* N3 behavior* or chang* N3 behavior* ) | 13,762 |
| S34 | TI performance or AB performance | 79,068 |
| S33 | TI ( “health care quality” or “healthcare quality” or quality W1 healthcare or quality W2 care ) or AB ( “health care quality” or “healthcare quality” or quality W1 healthcare or quality W2 care ) | 23,252 |
| S32 | TI ( quality W0 assurance or quality W0 improvement or quality W0 control ) or AB ( quality W0 assurance or quality W0 improvement or quality W0 control ) | 14,113 |
| S31 | TI practice N1 pattern* or AB practice N1 pattern* | 1,665 |
| S30 | TI ( clinical W0 practice* or medical W0 practice* or dental W0 practice* or private W0 practice* or general W0 practice* or family W0 practice* or professional W0 practice* or hospital* W0 practice* ) or AB ( clinical W0 practice* or medical W0 practice* or dental W0 practice* or private W0 practice* or general W0 practice* or family W0 practice* or professional W0 practice* or hospital* W0 practice* ) | 52,684 |
| S29 | TI ( “health personnel” N3 competence or “healthcare personnel” N3 competence or “health care personnel” N3 competence or physician N3 competence or physicians N3 competence or doctor N3 competence or doctors N3 competence or clinician N3 competence or clinicians N3 competence or nurse N3 competence or nurses N3 competence or provider N3 competence or providers N3 competence or practitioner N3 competence or practitioners N3 competence or resident N3 competence or residents N3 competence or professional N3 competence or professionals N3 competence or nursing N3 competence or clinical N3 competence ) or AB ( “health personnel” N3 competence or “healthcare personnel” N3 competence or “health care personnel” N3 competence or physician N3 competence or physicians N3 competence or doctor N3 competence or doctors N3 competence or clinician N3 competence or clinicians N3 competence or nurse N3 competence or nurses N3 competence or provider N3 competence or providers N3 competence or practitioner N3 competence or practitioners N3 competence or resident N3 competence or residents N3 competence or professional N3 competence or professionals N3 competence or nursing N3 competence or clinical N3 competence ) | 3,279 |
| S28 | TI ( “health personnel” N3 behavior or “healthcare personnel” N3 behavior or “health care personnel” N3 behavior or physician N3 behavior or physicians N3 behavior or doctor N3 behavior or doctors N3 behavior or clinician N3 behavior or clinicians N3 behavior or nurse N3 behavior or nurses N3 behavior or provider N3 behavior or providers N3 behavior or practitioner N3 behavior or practitioners N3 behavior or resident N3 behavior or residents N3 behavior or professional N3 behavior or professionals N3 behavior or nursing N3 behavior or clinical N3 behavior ) or AB ( “health personnel” N3 behavior or “healthcare personnel” N3 behavior or “health care personnel” N3 behavior or physician N3 behavior or physicians N3 behavior or doctor N3 behavior or doctors N3 behavior or clinician N3 behavior or clinicians N3 behavior or nurse N3 behavior or nurses N3 behavior or provider N3 behavior or providers N3 behavior or practitioner N3 behavior or practitioners N3 behavior or resident N3 behavior or residents N3 behavior or professional N3 behavior or professionals N3 behavior or nursing N3 behavior or clinical N3 behavior ) | 2,703 |
| S27 | TI ( “health personnel” N3 behavior or “healthcare personnel” N3 behavior or “health care personnel” N3 behavior or physician N3 behavior or physicians N3 behavior or doctor N3 behavior or doctors N3 behavior or clinician N3 behavior or clinicians N3 behavior or nurse N3 behavior or nurses N3 behavior or provider N3 behavior or providers N3 behavior or practitioner N3 behavior or practitioners N3 behavior or resident N3 behavior or residents N3 behavior or professional N3 behavior or professionals N3 behavior or nursing N3 behavior or clinical N3 behavior ) or AB ( “health personnel” N3 behavior or “healthcare personnel” N3 behavior or “health care personnel” N3 behavior or physician N3 behavior or physicians N3 behavior or doctor N3 behavior or doctors N3 behavior or clinician N3 behavior or clinicians N3 behavior or nurse N3 behavior or nurses N3 behavior or provider N3 behavior or providers N3 behavior or practitioner N3 behavior or practitioners N3 behavior or resident N3 behavior or residents N3 behavior or professional N3 behavior or professionals N3 behavior or nursing N3 behavior or clinical N3 behavior ) | 1,487 |
| S26 | TI ( “health personnel” N3 skills or “healthcare personnel” N3 skills or “health care personnel” N3 skills or physician N3 skills or physicians N3 skills or doctor N3 skills or doctors N3 skills or clinician N3 skills or clinicians N3 skills or nurse N3 skills or nurses N3 skills or provider N3 skills or providers N3 skills or practitioner N3 skills or practitioners N3 skills or resident N3 skills or residents N3 skills or professional N3 skills or professionals N3 skills or nursing N3 skills or clinical N3 skills ) or AB ( “health personnel” N3 skills or “healthcare personnel” N3 skills or “health care personnel” N3 skills or physician N3 skills or physicians N3 skills or doctor N3 skills or doctors N3 skills or clinician N3 skills or clinicians N3 skills or nurse N3 skills or nurses N3 skills or provider N3 skills or providers N3 skills or practitioner N3 skills or practitioners N3 skills or resident N3 skills or residents N3 skills or professional N3 skills or professionals N3 skills or nursing N3 skills or clinical N3 skills ) | 9,576 |
| S25 | TI ( “health personnel” N3 skill or “healthcare personnel” N3 skill or “health care personnel” N3 skill or physician N3 skill or physicians N3 skill or doctor N3 skill or doctors N3 skill or clinician N3 skill or clinicians N3 skill or nurse N3 skill or nurses N3 skill or provider N3 skill or providers N3 skill or practitioner N3 skill or practitioners N3 skill or resident N3 skill or residents N3 skill or professional N3 skill or professionals N3 skill or nursing N3 skill or clinical N3 skill ) or AB ( “health personnel” N3 skill or “healthcare personnel” N3 skill or “health care personnel” N3 skill or physician N3 skill or physicians N3 skill or doctor N3 skill or doctors N3 skill or clinician N3 skill or clinicians N3 skill or nurse N3 skill or nurses N3 skill or provider N3 skill or providers N3 skill or practitioner N3 skill or practitioners N3 skill or resident N3 skill or residents N3 skill or professional N3 skill or professionals N3 skill or nursing N3 skill or clinical N3 skill ) | 1,620 |
| S24 | (MH “Quality of Nursing Care”) | 8,802 |
| S23 | (MH “Quality of Health Care”) | 46,420 |
| S22 | (MH “Quality Assurance”) | 16,265 |
| S21 | (MH “Prescribing Patterns”) | 2,899 |
| S20 | (MH “Practice Patterns”) | 9,901 |
| S19 | (MH “Nursing Skills”) | 2,838 |
| S18 | (MH “Clinical Competence”) | 25,035 |
| S17 | (MH “Professional Competence”) | 10,651 |
| S16 | (MH “Professional Practice+”) | 185,938 |
| S15 | (MH “Hospitals+”) | 77,109 |
| S14 | (MH “Health Personnel+”) | 372,615 |
| S13 | S1 or S2 or S3 or S4 or S5 or S6 or S7 or S8 or S9 or S10 or S11 or S12 | 53,381 |
| S12 | TI benchmark* or AB benchmark* | 4,333 |
| S11 | TI hospital* W0 data or AB hospital* W0 data | 933 |
| S10 | TI “practice data” or AB “practice data” | 362 |
| S9 | TI “chart review” or AB “chart review” | 5,175 |
| S8 | TI review N3 record* or AB review N3 record* | 3,245 |
| S7 | TI feedback or AB feedback | 13,672 |
| S6 | TI ( audit or audits or auditing or feedback ) or AB ( audit or audits or auditing or feedback ) | 25,013 |
| S5 | (MH “Utilization Review”) | 1,657 |
| S4 | (MH “Feedback”) | 7,187 |
| S3 | (MH “Benchmarking”) | 5,172 |
| S2 | (MH “Nursing Audit”) | 853 |
| S1 | (MH “Audit”) | 11,752 |
