# Supplementary material for: Clinical Performance Feedback Intervention Theory (CP-FIT): a new theory for designing, implementing, and evaluating feedback in health care based on a systematic review and meta-synthesis of qualitative research
Source: Implement Sci. 2019 Apr 26;14:40. doi: 10.1186/s13012-019-0883-5 (PMC6486695; doi:10.1186/s13012-019-0883-5)
Supplement: Supplementary file 2 — Example data extraction form. (DOCX 61 kb) [file 13012_2019_883_MOESM2_ESM.docx]

**STUDY DETAILS**

| **Study ID** |  |
| --- | --- |
| **Date data extracted** |  |
| **Study Citation** |  |
| **Setting** |  |
| **Aim statement / research question of study (can be taken from the abstract)** |  |
| **Main findings / codes / framework / theory** |  |
| **Main changes to model (if any)** |  |

**RESEARCH PARAMETERS** [1]

| **#** | **COREQ reporting criteria** | **Describe if reported – otherwise “N”** |
| --- | --- | --- |
| 1 | Interviewer/facilitator: Which author/s conducted the interview or focus group? |  |
| 2 | Credentials: What were the researcher’s credentials? E.g. PhD, MD |  |
| 3 | Occupation: What was their occupation at the time of the study? |  |
| 4 | Gender: Was the researcher male or female? |  |
| 5 | Experience and training: What experience or training did the researcher have? |  |
| 6 | Relationship established: Was a relationship established prior to study commencement? |  |
| 7 | Participant knowledge of the interviewer: What did the participants know about the researcher? e.g. personal goals, reasons for doing the research |  |
| 8 | Interviewer characteristics: What characteristics were reported about the interviewer/facilitator? e.g. Bias, assumptions, reasons and interests in the research topic |  |
| 9 | Methodological orientation and Theory: What methodological orientation was stated to underpin the study? e.g. grounded theory, discourse analysis, ethnography, phenomenology, content analysis [NB – leave blank if do not explicitly mention] |  |
| 10 | Sampling: How were participants selected? e.g. purposive, convenience, consecutive, snowball |  |
| 11 | Method of approach: How were participants approached? e*.g. face-to-face, telephone, mail, email* |  |
| 12 | Sample size: How many participants were in the study? |  |
| 13 | Non-participation: How many people refused to participate or dropped out? Reasons? |  |
| 14 | Setting of data collection: Where was the data collected? e*.g. home, clinic, workplace* |  |
| 15 | Presence of non-participants: Was anyone else present besides the participants and researchers? |  |
| 16 | Description of sample: What are the important characteristics of the sample? *e.g. demographic data, date* |  |
| 17 | Interview guide: Were questions, prompts, guides provided by the authors? Was it pilot tested? |  |
| 18 | Repeat interviews: Were repeat interviews carried out? If yes, how many? |  |
| 19 | Audio/visual recording: Did the research use audio or visual recording to collect the data? |  |
| 20 | Field notes: Were field notes made during and/or after the interview or focus group? |  |
| 21 | Duration: What was the duration of the interviews or focus group? |  |
| 22 | Data saturation: Was data saturation discussed? |  |
| 23 | Transcripts returned: Were transcripts returned to participants for comment and/or correction? |  |
| 24 | Number of data coders: How many data coders coded the data? |  |
| 25 | Description of the coding tree: Did authors provide a description of the coding tree? |  |
| 26 | Derivation of themes: Were themes identified in advance or derived from the data? |  |
| 27 | Software: What software, if applicable, was used to manage the data? |  |
| 28 | Participant checking: Did participants provide feedback on the findings? |  |
| 29 | Quotations presented: Were participant quotations presented to illustrate the themes / findings? Was each quotation identified? e*.g. participant number* |  |
| 30 | Data and findings consistent: Was there consistency between the data presented and the findings? |  |
| 31 | Clarity of major themes: Were major themes clearly presented in the findings? |  |
| 32 | Clarity of minor themes: Is there a description of diverse cases or discussion of minor themes? |  |

**INTERVENTION**

| **Has the intervention been judged a success? How?** |  |
| --- | --- |
| **How was the intervention implemented / delivered? Use the Behaviour Change Tehcnique Taxonomy** **where possible** [2] |  |

| **Modifiable elements** [3] (*plus additional) | **Describe (briefly) or Unclear** |
| --- | --- |
| 1. Was the feedback given to an individual, a group or both? |  |
| 2. Was it given to the person in whom the practice change was desired (eg, healthcare provider vs hospital administrator) |  |
| 3. Was there feedback about the processes of care (eg, rate of antibiotic prescription) |  |
| 4. Was there feedback about patient outcomes |  |
| 5. Was there feedback about something other than processes of care or patient outcomes (if yes, specified) |  |
| 6. Was the feedback about individual provider performance |  |
| 7. Was the feedback about the performance of the provider group |  |
| 8. Was the feedback about individual patient cases |  |
| 9. Was the feedback about an aggregate of patient cases |  |
| 10. Did the feedback identify a specific behaviour(s) to be changed |  |
| 11. What was the comparison provided in the feedback (specified) |  |
| 12. Were graphical elements included in the feedback |  |
| 13. What was the lag between the time of the audit and the delivery of the feedback (days, weeks, months, years, a mix) |  |
| 14. What rationale was given for using A&F (specified)  *[e.g. What are its theoretical underpinnings?*  *(Either explicit references to theory, or implied mechanisms of change by authors)]* |  |
| 15. Was the feedback given face to face |  |
| 16. Were providers explicitly asked to consider the implications the A&F had for their practice  *[e.g. action planning]* |  |
| 17. What was the total number of times the feedback was given (specified). |  |
| *What was the task being studied?** |  |
| *How was the audit performed?** |  |
| *What data was used to perform the audit?** |  |

**QUALITY APPRAISAL** [4]

| **Broad areas** | **Criteria** | **Each criterion**  (Y / N / N/A) | **Overall judgment**  (Y / N / N/A) |
| --- | --- | --- | --- |
| Clear statement of, and rationale for, research question/aims/purposes | Clarity of focus demonstrated |  |  |
|  | Explicit purpose given, such as descriptive/explanatory intent, theory building, hypothesis testing |  |  |
|  | Link between research and existing knowledge demonstrated |  |  |
| Study thoroughly contextualised by existing literature | Evidence of systematic approach to literature review, location of literature to contextualise the findings, or both |  |  |
| Method/design apparent, and consistent with research intent | Rationale given for use of qualitative design |  |  |
|  | Discussion of epistemological/ontological grounding |  |  |
|  | Rationale explored for specific qualitative method (e.g. ethnography, grounded theory, phenomenology) |  |  |
|  | Discussion of why particular method chosen is most appropriate/sensitive/relevant for research question/aims |  |  |
|  | Setting appropriate |  |  |
| Data collection strategy apparent and appropriate | Were data collection methods appropriate for type of data required and for specific qualitative method? |  |  |
|  | Were they likely to capture the complexity/diversity of experience and illuminate context in sufficient detail? |  |  |
|  | Was triangulation of data sources used if appropriate? |  |  |
| Sample and sampling method appropriate | Selection criteria detailed, and description of how sampling was undertaken |  |  |
|  | Justification for sampling strategy given |  |  |
|  | Thickness of description likely to be achieved from sampling |  |  |
|  | Any disparity between planned and actual sample explained |  |  |
| Analytic approach appropriate | Approach made explicit (e.g. Thematic distillation, constant comparative method, grounded theory) |  |  |
|  | Was it appropriate for the qualitative method chosen? |  |  |
|  | Was data managed by software package or by hand and why? |  |  |
|  | Discussion of how coding systems/conceptual frameworks evolved |  |  |
|  | How was context of data retained during analysis |  |  |
|  | Evidence that the subjective meanings of participants were portrayed |  |  |
|  | Evidence of more than one researcher involved in stages if appropriate to epistemological/theoretical stance |  |  |
|  | Did research participants have any involvement in analysis (e.g. member checking) |  |  |
|  | Evidence provided that data reached saturation or discussion/rationale if it did not |  |  |
|  | Evidence that deviant data was sought, or discussion/ rationale if it was not |  |  |
| Context described and taken account of in interpretation | Description of social/physical and interpersonal contexts of data collection |  |  |
|  | Evidence that researcher spent time ‘dwelling with the data’, interrogating it for competing/alternative explanations of phenomena |  |  |
| Clear audit trail given | Sufficient discussion of research processes such that others can follow ‘decision trail’ |  |  |
| Data used to support interpretation | Extensive use of field notes entries/verbatim interview quotes in discussion of findings |  |  |
|  | Clear exposition of how interpretation led to conclusions |  |  |
| Researcher reflexivity demonstrated | Discussion of relationship between researcher and participants during fieldwork |  |  |
|  | Demonstration of researcher’s influence on stages of research process |  |  |
|  | Evidence of self-awareness/insight |  |  |
|  | Documentation of effects of the research on researcher |  |  |
|  | Evidence of how problems/complications met were dealt with |  |  |
| Demonstration of sensitivity to ethical concerns | Ethical committee approval granted |  |  |
|  | Clear commitment to integrity, honesty, transparency, equality and mutual respect in relationships with participants |  |  |
|  | Evidence of fair dealing with all research participants |  |  |
|  | Recording of dilemmas met and how resolved in relation to ethical issues |  |  |
|  | Documentation of how autonomy, consent, confidentiality, anonymity were managed |  |  |
| Relevance and transferability evident | Sufficient evidence for typicality specificity to be assessed |  |  |
|  | Analysis interwoven with existing theories and other relevant explanatory literature drawn from similar settings and studies |  |  |
|  | Discussion of how explanatory propositions/emergent theory may fit other contexts |  |  |
|  | Limitations/weaknesses of study clearly outlined |  |  |
|  | Clearly resonates with other knowledge and experience |  |  |
|  | Results/conclusions obviously supported by evidence |  |  |
|  | Interpretation plausible and ‘makes sense’ |  |  |
|  | Provides new insights and increases understanding |  |  |
|  | Significance for current policy and practice outlined |  |  |
|  | Assessment of value/empowerment for participants |  |  |
|  | Outlines further directions for investigation |  |  |
|  | Comment on whether aims/purposes of research were achieved |  |  |
| **Total** | | |  |

**MAIN STRENGTHS/LIMITATIONS**

| **Main strengths of study**  (according to reviewer – i.e. you) |  |
| --- | --- |
| **Main strengths of study**  (according to study author) |  |
| **Main limitations of study**  (according to reviewer – i.e. you) |  |
| **Main limitations of study**  (according to study author) |  |

**SUPPLEMENTARY SEARCHES**

| **Papers in bibliography that may be relevant (out of how many?)**   - Out of how many? - New papers to synthesise or for background discussion |  |
| --- | --- |
| **Papers in citation searching that may be relevant (using WoS)**   - Out of how many? - New papers to synthesise or for background discussion |  |
| **Papers in related article searching that may be relevant (Using WoS – limit to first 100 results)**   - Out of how many? - New papers to synthesise or for background discussion |  |

**MEMOS**

|  |
| --- |

**References**

[1] A. Tong, P. Sainsbury, J. Craig, Consolidated criteria for reporting qualitative research (COREQ): a 32-item checklist for interviews and focus groups, Int J Qual Heal. Care. 19 (2007) 349–357.

[2] S. Michie, M. Richardson, M. Johnston, C. Abraham, J. Francis, W. Hardeman, et al., The behavior change technique taxonomy (v1) of 93 hierarchically clustered techniques: building an international consensus for the reporting of behavior change interventions., Ann. Behav. Med. 46 (2013) 81–95. doi:10.1007/s12160-013-9486-6.

[3] H. Colquhoun, S. Michie, A. Sales, N. Ivers, J.M. Grimshaw, K. Carroll, et al., Reporting and design elements of audit and feedback interventions: a secondary review, BMJ Qual Saf. (2016) 1–7. doi:10.1136/bmjqs-2015-005004.

[4] D. Walsh, S. Downe, Appraising the quality of qualitative research, Midwifery. 22 (2006) 108–119. doi:10.1016/j.midw.2005.05.004.
