# Supplementary material for: Clinical Performance Feedback Intervention Theory (CP-FIT): a new theory for designing, implementing, and evaluating feedback in health care based on a systematic review and meta-synthesis of qualitative research
Source: Implement Sci. 2019 Apr 26;14:40. doi: 10.1186/s13012-019-0883-5 (PMC6486695; doi:10.1186/s13012-019-0883-5)
Supplement: Supplementary file 3 — Data synthesis method. (DOCX 171 kb) [file 13012_2019_883_MOESM3_ESM.docx]

**Appendix 3: Data synthesis method**

**Stage 1: Code findings from individual papers**

Papers were independently coded line-by-line in batches of four by BB and WG using Framework Analysis^1^ informed by Realistic Evaluation.^2^ Excerpts were entered as separate rows in a spreadsheet with associated thematic codes in adjacent columns relating to features of the intervention, context, resultant outcomes, and potential explanatory mechanisms.^2^ These were initially developed inductively from study findings:^3^ intervention codes described the design or implementation of feedback (e.g. how clinical performance data were analysed, or feedback was presented); context codes described anything existing prior to the intervention’s introduction (e.g. characteristics of the organisation or health professional); outcome codes described consequences of introducing the intervention (e.g. health professionals understanding their performance, or taking action to improve patient care); and mechanism codes attempted to explain how the outcome may have occurred (e.g. because the feedback design was easier to understand or take action based upon). After coding each batch of manuscripts, BB and WG compared findings and resolved discrepancies through discussion.

**Stage 2: Generalise findings across papers and build on pre-existing theories to formulate hypotheses**

Each batch of coded manuscripts was discussed with two further independent reviewers (GDW and TB). The aim was to critique the line-by-line coding done in Stage 1,^4^ and identify demi-regularities between studies to formulate hypotheses about how feedback interventions work in general.^5^ A codebook of higher-level constructs was created, with definitions, diagrams, and descriptions of their inter-relationships to support our emerging hypotheses.^1^ Where possible, we built on constructs from relevant existing behaviour change theories to develop our ideas and avoid synonymy rather than coining new terms (see ‘Identifying behaviour change theories’ below) ^6^.

**Stage 3: Test and refine**

Stages 1 and 2 were repeated on further batches of studies by applying the codebook deductively in order to test our emerging hypotheses, iteratively improving them via a modified version of Analytic Induction.^7^ New studies either supported a particular hypothesis or construct, or led to its adjustment or falsification.

**Stage 4: Assess confidence in findings**

After all papers had been synthesised, their excerpts and codes from Stage 1 were transferred into Nvivo (version 10; QSR International). Author BB used Matrix Coding to assess the confidence in our findings according to the GRADE-CERQual method.^8^ For each hypothesis we generated, the supporting studies were evaluated for: methodological limitations (see ‘Study selection and data extraction’ section in main manuscript); relevance; coherence; and adequacy.^8^ Based on these criteria, a judgment of either ‘high’, ‘moderate’, or ‘low’ confidence in the finding was made.^8^ The final list of hypotheses and their GRADE-CERQual ratings were agreed through iterative discussion with the review team.

**Stage 5: Distil hypotheses into propositions**

To create a usable and parsimonious theory, we summarised hypotheses with ‘high’ GRADE-CERQual ratings into a core set of propositions. We iteratively formulated and refined potentially suitable statements, returning to paper excerpts from Stage 1 as necessary, until agreed by the entire review team.

**Identifying behaviour change theories**

We identified relevant existing behaviour change theories (both general and specific to feedback) in order to: help explain and organise our findings, build on existing bodies of knowledge to advance our understanding, place our results in the wider context of associated literature, and avoid synonymy with existing theories rather than coining new terms. We defined behaviour change theories as theories that “seek to explain why, when and how a behaviour does or does not occur, and the important sources of influence to be targeted in order to alter the behaviour”.^9^ We identified potentially suitable theories from: included primary studies in the review; supplementary searches to identify further primary studies (see ‘Search strategy’ section in main manuscript); international experts;^10^ a compendium of behaviour change theories;^9^ and a systematic review of theory in feedback intervention research.^11^ We also conducted a separate systematic literature search following Booth and Carroll’s methodology,^12^ which used the following search string: *((quality OR performance OR clinical) AND (audit OR feedback OR measurement OR indicator OR dashboard OR scorecard OR “report card”)) AND (theor* OR framework* OR model* OR concept*)*. We executed the search in December 2015 in Google Scholar (in order to search manuscripts’ full text as these topics are often not only present in abstracts). Any paper felt to potentially contain description of, or reference to, a behaviour change theory was read in full by reviewer BB. This was limited to the first 500 results in Google Scholar to maintain manageability and relevance. Each identified behaviour change theory was assessed for its: explanatory power (did it explain our emerging codes and hypotheses?); clarity (did it contain unambiguous concepts?); implementation (could its concepts be operationalised consistently by coders?); and testability (were its propositions falsifiable?).^13^ All criteria had to be met in order to contribute to the metasynthesis. Relevant constructs from included theories were used to: develop the names and descriptions of codes in our codebook, explain our emerging hypothesis, and suggest further concepts to explore in our data. The full list of theories we considered for inclusion were:

1. Action Theory Model of Consumption (Bagozzi)
2. Adult Learning Theory (Knowles)
3. Affective Events Theory (Weiss & Cropanzano)
4. Aids Risk Reduction Model (Catania et al.)
5. Ancillary Benefits of Performance Measurement Model (Powell et al.)
6. Awareness-to-adherence model (Pathman et al.)
7. Bearing Bad News (Ilgen & Davis)
8. Behavioural-Ecological Model of Adolescent Aids Prevention (Hovell et al.)
9. Behavioral Psychology Theory (Ullman & Krasner)
10. CEOS Theory (Borland)
11. Change Theory (Lewin)
12. Classical Conditioning (Pavlov)
13. Clinical Practice Guideline Adherence Model (Cabana et al.)
14. Cognitive Adaptation Theory (Taylor)
15. Cognitive Dissonance (Festinger)
16. Cognitive Load Theory (Sweller et al.)
17. COM-B System (Michie et al.)
18. Consolidated Framework for Implementation Research (Damschroder et al.)
19. Consumption as Social Practices (Spaargaren & Van Vliet)
20. Containment Theory (Reckless)
21. Contemporary performance measurement systems (Franco-santos et al.)
22. Control Theory (Carver & Scheier)
23. Differential Association Theory (Sutherland)
24. Diffusion of Innovations (Rogers)
25. Diffusion of Innovations in Service Organizations (Greenhalgh et al.)
26. Dual Processing Theory (Kahneman)
27. Ecological Model for Preventing Type 2 Diabetes in Minority Youth (Burnet et al.)
28. Extended Information Processing Model (Flay et al.)
29. Extended Parallel Processing Model (Witte)
30. Feedback Intervention Theory (Kluger & DeNisi)
31. Feedforward Theory (Kluger & Van Dijk)
32. Fit between Individuals, Task and Technology framework (FITT; Ammenwerth et al.)
33. Focus Theory of Normative Conduct (Cialdini et al.)
34. Formative Feedback Model (Shute)
35. General Theory of Crime (Gottfredson & Hirschi)
36. General Theory of Deviant Behaviour (Kaplan)
37. Goal Directed Theory (Bagozzi)
38. Goal-Framing Theory (Lindenberg & Steg)
39. Goal Setting Theory (Locke & Latham)
40. Guideline Interdependence Model (Pawson et al.)
41. Health Action Process Approach (Schwarzer)
42. Health Behaviour Goal Model (Maes & Gebhardt)
43. Health Behaviour Internalisation Model (Bellg)
44. Health Belief Model (Rosenstock)
45. Health Promotion Model (Pender et al.)
46. Hierarchy of Levels of Evaluation (Kirkpatrick)
47. Hierarchical Model of Intrinsic and Extrinsic Motivation (Vallerand 1997)
48. Human Error Theory (Reason)
49. I-Change Model (De Vries et al.)
50. Implementation Intentions (Gollwitzer)
51. Individual Feedback Theory (Ilgen et al.)
52. Information-Motivation-Behavioural Skills Model (Fisher & Fisher)
53. Information-Motivation-Behavioural Skills Model of Adherence (Fisher et al.)
54. Informed Self-Assessment Model (Sargeant et al.)
55. Integrated Theoretical Model for Alcohol and Other Drug Abuse Prevention (Gonzalez)
56. Integrated Theory of Drinking Behaviour (Wagenaar & Perry)
57. Integrated Theory of Health Behaviour Change (Ryan)
58. Integrative Model of Behavioural Prediction (Fishbein)
59. Integrative Model of Factors Influencing Smoking Behaviours (Flay et al.)
60. Integrative Model of Health Attitude and Behaviour Change (Flay)
61. Integrative Model of Factors Influencing Smoking And Attitude And Health Behaviour Change (Flay et al.)
62. Knowledge-to-Action Cycle (Straus & Holroyd-Leduc)
63. Locus of Control (Rotter)
64. Locus of Performance Assessment (Exworthy et al.)
65. Model Depicting Impact of Performance Feedback on Physician Patient-Management Behavior (Payne & Hysong)
66. Model of Information Systems Success (Delone & McLean)
67. Model of Pro-Environmental Behaviour (Kollmuss & Agyeman)
68. Model That Builds Relationship, Explores Reactions and Content, and Coaches for Performance Change (R2C2; Sargeant et al.)
69. Motivation-Opportunities-Abilities Model (Ölander & Thøgersen)
70. Motivation Theory (Pritchard & Ashwood)
71. Multilevel approach to change (Ferlie & Shortell)
72. Needs-Opportunities-Abilities Model (Gatersleben & Vlek)
73. Norm Activation Theory (Schwartz)
74. Normalisation Process Theory (May)
75. Normative Social Influence (Asch)
76. Obedience to authority (Milgram)
77. Operant Learning Theory (Skinner)
78. Organisational Learning Theory (Nonaka & Takeuchi)
79. Organization Theory (Lawler)
80. Organization Development Theory (Levitt)
81. Patient-Reported Outcomes Measure Model (Boyce et al.)
82. Precaution Adoption Process Model (Weinstein & Sandman)
83. Pressure System Model (Katz)
84. PRIME Theory (West)
85. Persuasion Theory (Cialdini)
86. Precede/Proceed Planning Model (Green & Kreeuter)
87. Problem Behaviour Theory (Jessor)
88. Promoting Action on Research Implementation in Health Services (PARiHS; Rycroft-Malone)
89. Prospect Theory (Kahneman & Tversky)
90. Protection Motivation Theory (Rogers)
91. Prototype Willingness Model (Gerrard et al.)
92. Rational Addiction Model (Becker & Murphy)
93. Reflective Impulsive Model (Strack & Deutsch)
94. Reference Group Theory (Merton)
95. Regional Audit Model (Paskins et al.)
96. Regulatory Fit Theory (Higgins)
97. Regulatory Focus Theory (Higgins)
98. Relapse Prevention Model (Marlatt & Gordon)
99. Risk as Feelings Theory (Lowenstein et al.)
100. Self-Affirmation Theory (Steele)
101. Self-Determination Theory (Deci & Ryan)
102. Self-Efficacy Theory (Bandura)
103. Self-Regulation Theory (Kanfer & Gaelick)
104. Six Staged Model of Communication Effects (Vaughan & Everett)
105. Social Action Theory (Ewart)
106. Social Action Theory (Weber)
107. Social Change Theory (Thompson & Kinne)
108. Social Cognitive Theory (Bandura)
109. Social Comparison Theory (Festinger)
110. Social Consensus Model of Health Education (Romer & Hornik)
111. Social Development Model (Hawkins & Weis)
112. Social Ecological Model of Behaviour Change (Panter-Brick et al.)
113. Social Ecological Model of Walking (Alfonzo)
114. Social Facilitation (Michaels et al.)
115. Social Impact Theory (Latané)
116. Social Identity Theory (Tajfel & Turner)
117. Social Influence Model of Consumer Participation (Dholakia et al.)
118. Social Judgment Theory (Sherif et al.)
119. Social Learning Theory (Miller & Dollard)
120. Social Loafing (Ringelmann)
121. Social Norms Theory (Perkins & Berkowitz)
122. Structuration theory (Giddens; Stones)
123. Systems Model of Health Behaviour Change (Kersell & Milsum)
124. Task-Technology-Fit Model (TTF; Goodhue & Thompson)
125. Technology Acceptance Model (Davis; Venkatesh & Davis; Venkatesh & Bala)
126. Technology Diffusion Theory (Penland)
127. Temporal Self-Regulation Theory (Hall & Fong)
128. Terror Management Theory (Greenberg et al.)
129. Terror Management Health Model (Goldenberg & Arndt)
130. Theory of Cognitive Dissonance (Festinger)
131. Theory of Interpersonal Behaviour (Triandis)
132. Theory of Medical Education (Coles & Holm)
133. Theory of Normative Social Behaviour (Rimal & Real)
134. Theory of Planned Behaviour (Ajzen)
135. Theory of Reasoned Action (Fishbein)
136. Theory of Triadic Influence (Flay & Petraitis)
137. Transcontextual Model of Motivation (Hagger et al.)
138. Transtheoretical Model of Behaviour Change (Prochaska & DiClemente)
139. Unified Theory of Acceptance and Use of Technology (UTAUT; Venkatesh at al.)
140. Unintended Consequences of Performance Measurement (Powell at al.)
141. Value Belief Norm Theory (Stern et al.)
142. Value Chain of Information (Coiera)

**References**

1. Ritchie J, Spencer L. Qualitative data analysis for applied policy research. In: Bryman A, Burgess R, editors. Analysing Qualitative Data. Routledge; 1994. p. 173–94.

2. Pawson R, Tilley N. Realistic Evaluation. Sage Publications (CA); 1997. 256 p.

3. Thomas J, Harden A. Methods for the thematic synthesis of qualitative research in systematic reviews. BMC Med Res Methodol. 2008 Jan;8:45.

4. Lincoln Y, Guba E. Naturalistic Inquiry. Newbury Park: Sage; 1985.

5. Pawson R. Evidence-Based Policy：A Realist Perspective. London: SAGE Publications; 2006.

6. Brehaut JC, Eva KW. Building theories of knowledge translation interventions: use the entire menu of constructs. Implement Sci. Implementation Science; 2012 Jan;7(1):114.

7. Byng R, Norman I, Redfern S. Using Realistic Evaluation to Evaluate a Practice-level Intervention to Improve Primary Healthcare for Patients with Long-term Mental Illness. Evaluation. 2005;11(1):69–93.

8. Lewin S, Glenton C, Munthe-Kaas H, Carlsen B, Colvin CJ, Gülmezoglu M, et al. Using Qualitative Evidence in Decision Making for Health and Social Interventions: An Approach to Assess Confidence in Findings from Qualitative Evidence Syntheses (GRADE-CERQual). PLOS Med. 2015;12(10):e1001895.

9. Michie S, West R, Campbell R, Brown J, Gainforth H. ABC of Behaviour Change Theories. London: Silverback Publishing; 2014.

10. Ottawa Hospital Research Institute. The Audit & Feedback MetaLab [Internet]. 2016. Available from: http://www.ohri.ca/auditfeedback/

11. Colquhoun HL, Brehaut JC, Sales A, Ivers N, Grimshaw J, Michie S, et al. A systematic review of the use of theory in randomized controlled trials of audit and feedback. Implement Sci. Implementation Science; 2013 Jan;8(1):66.

12. Booth A, Carroll C. Systematic searching for theory to inform systematic reviews: is it feasible? Is it desirable? Heal Info Libr J. Health Economics & Decision Science (HEDS), School of Health & Related Research (ScHARR), University of Sheffield, Sheffield, UK Health Economics & Decision Science (HEDS), School of Health & Related Research (ScHARR), University of Sheffield, Sheffield, ; 2015 Jun 11;(1471–1842 (Electronic)).

13. Noyes J, Hendry M, Booth A, Chandler J, Lewin S, Glenton C, et al. Current use and Cochrane guidance on selection of social theories for systematic reviews of complex interventions. J Clin Epidemiol. Elsevier Ltd; 2016;75:78–92.
