# Supplementary material for: Clinical Performance Feedback Intervention Theory (CP-FIT): a new theory for designing, implementing, and evaluating feedback in health care based on a systematic review and meta-synthesis of qualitative research
Source: Implement Sci. 2019 Apr 26;14:40. doi: 10.1186/s13012-019-0883-5 (PMC6486695; doi:10.1186/s13012-019-0883-5)
Supplement: Supplementary file 4 — Study details. (DOCX 155 kb) [file 13012_2019_883_MOESM4_ESM.docx]

**Additional file 4: Study details**

| **ID** | **Citation** | **Country** | **Setting** | **Feedback topic(s)** | **Target recipient(s)** | **Number of study participants** | **Brief description of feedback intervention** | **Quality appraisal (out of 12)** |
| --- | --- | --- | --- | --- | --- | --- | --- | --- |
| 187 | A.M. Grant, B. Guthrie, T. Dreischulte, Developing a complex intervention to improve prescribing safety in primary care: mixed methods feasibility and optimisation pilot study., BMJ Open. 4 (2014) e004153. doi:10.1136/bmjopen-2013-004153. | Scotland | Primary care | Medication safety | Physicians | 10 | Lists of patients provided to primary care practices by research team. | 5 |
| 1591 | K.M. Cresswell, S. Sadler, S. Rodgers, A. Avery, J. Cantrill, S. a Murray, et al., An embedded longitudinal multi-faceted qualitative evaluation of a complex cluster randomized controlled trial aiming to reduce clinically important errors in medicines management in general practice., Trials. 13 (2012) 78. doi:10.1186/1745-6215-13-78. | England | Primary care | Medication safety | Physicians | 52 | Pharmacist-led feedback where lists of patients provided to primary care practices. | 9 |
| 2249 | M. Exworthy, E.K. Wilkinson, A. McColl, M. Moore, P. Roderick, H. Smith, et al., The role of performance indicators in changing the autonomy of the general practice profession in the UK, Soc. Sci. Med. 56 (2003) 1493–1504. doi:10.1016/S0277-9536(02)00151-X. | England | Primary care | Chronic care | Physicians Nurses | 29 | Face-to-face presentation of feedback to primary care practice by research team. | 9 |
| 7301 | K. Stevenson, R. Baker, A. Farooqi, R. Sorrie, K. Khunti, Features of primary health care teams associated with successful quality improvement of diabetes care : a qualitative study, Fam Pr. 18 (2001) 21–6. | England | Primary care | Diabetes | Physicians Nurses | 18 | Feedback intervention conducted by external group to primary care practice. | 6 |
| 627 | S. Barklie, K. Stevenson, Why do some practices fall behind schedule when undertaking a multipractice audit? Lessons for Clincal Governors?, J. Clin. Governance1. 7 (1999) 181–185. | England | Primary care | Diabetes | Physicians Nurses | 10 | Primary care practices conducted Feedback intervention themselves, some had help from facilitators. | 4 |
| 2857 | T.L. Guldberg, P. Vedsted, T. Lauritzen, V. Zoffmann, Suboptimal quality of type 2 diabetes care discovered through electronic feedback led to increased nurse-GP cooperation. A qualitative study., Prim. Care Diabetes. 4 (2010) 33–39. | Denmark | Primary care | Diabetes | Physicians Nurses | 13 | Computer programme that provides lists of patients. | 8 |
| 7194 | J. Søndergaard, M. Andersen, J. Kragstrup, P. Hansen, L. Freng Gram, Why has postal prescriber feedback no substantial impact on general practitioners’ prescribing practice? A qualitative study., Eur. J. Clin. Pharmacol. 58 (2002) 133–6. doi:10.1007/s00228-002-0455-4. | Denmark | Primary care | Prescribing (general) | Physicians | 8 | Unsolicited mailed feedback conducted by researchers. | 8 |
| S1 | N. Ivers, J. Barnsley, R. Upshur, K. Tu, B. Shah, J. Grimshaw, et al., My approach to this job is ... one person at a time, Can Fam Physician. 60 (2014) 258–266. | Canada | Primary care | Chronic care | Physicians | 12 | Mailed feedback conducted by researchers. | 10 |
| 7783 | B. Vachon, B. Desorcy, M. Camirand, J. Rodrigue, L. Quesnel, C. Guimond, et al., Engaging primary care practitioners in quality improvement: making explicit the program theory of an interprofessional education intervention., BMC Health Serv. Res. 13 (2013) 106. | Canada | Primary care | Diabetes | Physicians, nurses, pharmacists, and other professionals | 17 | Feedback provided face-to-face by researchers, in addition to a workshop. | 7 |
| S6 | E.K. Wilkinson, A. McColl, M. Exworthy, P. Roderick, H. Smith, M. Moore, et al., Reactions to the use of evidence-based performance indicators in primary care: a qualitative study., Qual. Health Care. 9 (2000) 166–74. | England | Primary care | Chronic care | Physicians Nurses | 52 | See Exworthy et al. 2003 | 10 |
| S7 | S. Johnston, M. Green, P. Thille, C. Savage, L. Roberts, G. Russell, et al., Performance feedback: an exploratory study to examine the acceptability and impact for interdisciplinary primary care teams., BMC Fam. Pract. 12 (2011) 14. | Canada | Primary care | Chronic care, patient experience | Physicians Nursing Staff Nurse Practitioners Pharmacists Social Workers Dieticians Management Administrative Support Staff | 32 | Research team provided face-to-face feedback in groups. | 7 |
| S4 | A. Powell, K.M. White, M.R. Partin, K. Halek, S.J. Hysong, E. Zarling, et al., More than a score: a qualitative study of ancillary benefits of performance measurement., BMJ Qual. Saf. (2014) 1–8. doi:10.1136/bmjqs-2013-002149. | US | Primary care | Chronic care | Facility administrators Quality improvement/PM specialists Primary care physician/nurse leaders Physicians Non-physician practitioners Intake nurses | 60 | Quarterly performance measurement and feedback, linked to pay-for-performance. | 8 |
| S15 | E. Nouwens, J. van Lieshout, M. Wensing, Determinants of impact of a practice accreditation program in primary care: a qualitative study., BMC Fam. Pract. 16 (2015) 78. doi:10.1186/s12875-015-0294-x. | Netherlands | Primary care | Chronic care, health care structure, patient experience | Physicians Nurses | 33 | Written feedback provided by external team, which is discussed with a trained observer with the whole practice team. Practice are forced to write action plans. | 6 |
| S28 | M.S. Rowan, W. Hogg, C. Martin, E. Vilis, Family physicians’ reactions to performance assessment feedback., Can. Fam. Physician. 52 (2006) 1570–1571. | Canada | Primary care | Preventive medicine, patient experience | Physicians | 8 | Feedback provided in face-to-face session by external team. | 8 |
| S30 | H. Beckman, A.L. Suchman, K. Curtin, R.A. Greene, Physician Reactions to Quantitative Individual Performance Reports, Am J Med Qual. 21 (2006) 192–199. doi:10.1177/1062860606287577. | US | Primary care | Chronic care, paediatrics, patient experience | Physicians | 19 | Mailed feedback reports by external organisation, linked to pay-for-performance. | 4 |
| 6087 | A.A. Powell, K.M. White, M.R. Partin, K. Halek, J.B. Christianson, B. Neil, et al., Unintended consequences of implementing a national performance measurement system into local practice, J. Gen. Intern. Med. 27 (2012) 405–412. doi:10.1007/s11606-011-1906-3. | US | Primary care | Chronic care | Facility administrators QI / PM specialists Clinic physician leaders Clinic nurse leaders Physicians Non-physician practitioners Intake nurses | 60 | See Powell et al. 2014 | 8 |
| S32 | M.L. Lippert, M.B. Kousgaard, L. Bjerrum, General practitioners uses and perceptions of voluntary electronic feedback on treatment outcomes – a qualitative study, BMC Fam. Pract. 15 (2014) 193. doi:10.1186/s12875-014-0193-6. | Denmark | Primary care | Chronic care | Physicians | 9 | Computerised feedback with benchmarking and patient lists. | 8 |
| 5033 | R. Mannion, M. Goddard, Impact of published clinical outcomes data: case study in NHS hospital trusts., BMJ. 323 (2001) 260–3. doi:10.1136/bmj.323.7307.260. | Scotland | Hospital | Cancer surgery, stroke | Physicians Nurses Managers | 48 | National audit published by health system. | 8 |
| 5532 | C. Nessim, C.M. Bensimon, B. Hales, C. Laflamme, D. Fenech, A. Smith, Surgical site infection prevention: A qualitative analysis of an individualized audit and feedback model, J. Am. Coll. Surg. 215 (2012) 850–857. doi:10.1016/j.jamcollsurg.2012.08.007. | Canada | Hospital | General surgery | Surgeons Anaesthetists Junior doctors Nurses | 18 | Personalised paper-based feedback: Case-specific compliance (patient lists). Aggregated results to nursing group and residents. | 6 |
| 8167 | F.C. Wright, M. Fitch, A.J. Coates, M. Simunovic, A qualitative assessment of a provincial quality improvement strategy for pancreatic cancer surgery., Ann. Surg. Oncol. 18 (2011) 629–635. | Canada | Hospital | Cancer surgery | Surgeons Adminstrators | 24 | Written feedback provided by regional organisation regarding hospital-level performance on pancreas surgery outcomes. | 8 |
| S52 | A.I.G. Ramsay, S. Turner, G. Cavell, C.A. Oborne, R.E. Thomas, G. Cookson, et al., Governing patient safety: lessons learned from a mixed methods evaluation of implementing a ward-level medication safety scorecard in two English NHS hospitals., BMJ Qual. Saf. 23 (2014) 136–46. doi:10.1136/bmjqs-2012-001730. | England | Hospital | Medication safety | Nurses Physicians | 49 | Face-to-face feedback by researchers including discussing possible reasons and solutions for suboptimal performance. | 5 |
| 7694 | S. Turner, J. Higginson, C.A. Oborne, R.E. Thomas, A.I.G. Ramsay, N.J. Fulop, Codifying knowledge to improve patient safety: A qualitative study of practice-based interventions, Soc. Sci. Med. 113 (2014) 169–176. doi:10.1016/j.socscimed.2014.05.031. | England | Hospital | Medication safety | Nurses Physicians | 10 | See Ramsay et al. 2014 | 8 |
| 1948 | M. Dixon-Woods, S. Redwood, M. Leslie, J. Minion, G.P. Martin, J.J. Coleman, Improving quality and safety of care using “technovigilance”: an ethnographic case study of secondary use of data from an electronic prescribing and decision support system., Milbank Q. 91 (2013) 424–454. | England | Hospital | Medication safety | Physicians Nurses Managers | 10 | Secondary use of data from clinical decision support systems in a hospital to provide feedback to clinicians and managers. Part of routine practice. | 11 |
| 512 | B. Seip, J. Frich C., G. Hoff, Physicians’ experiences with a quality assurance programme., Clin. Gov. An Int. J. 17 (2012) 297–306. | Norway | Hospital - outpatient | Gastroscopy | Physicians Nurses Managers | 8 | Written reports provided to doctors. | 7 |
| 1271 | M. Cameron, G. Penney, G. McLeer, M. Sharon, A. Walker, Impact on Maternity Professionals of Novel Audit Feedback, (2015) 75–95. | Scotland | Hospital | Obstetrics | Physicians Midwives | 17 | Three different interventions delivered by research team: 1. Printed feedback (anonymized service-level data); 2. + action planning letter; 3. 2 + Facilitated action planning face-to-face. | 6 |
| 2023 | S. Dunn, A.E. Sprague, D.B. Fell, J. Dy, J. Harrold, B. Lamontagne, et al., The use of a quality indicator to reduce elective repeat Caesarean section for low-risk women before 39 weeks’ gestation: the Eastern Ontario experience., J. Obstet. Gynaecol. Canada. 35 (2013) 306–316. | Canada | Hospital | Obstetrics | Physicians Nurses Managers | 9 | 1 indicator – unnecessary caesarean. Data from a database. Support from research team. | 5 |
| 7816 | J.H.M. Veillard, M.L. Schiøtz, A.L. Guisset, A.D. Brown, N.S. Klazinga, The PATH project in eight European countries: An evaluation, Int. J. Health Care Qual. Assur. 26 (2013) 703–713. doi:10.1108/IJHCQA-11-2011-0065. | Belgium, Estonia, France, Germany, Hungary, Poland, Slovakia and Slovenia | Hospital | Obstetrics,  Antimicrobial stewardship,  Mortality,  Admission rates,  Staff experience,  Health care structures,  Patient experience | Physicians, nurses, managers | 20 | Multinational project. Feedback was calculated and results fed back to participating hospital managers using a hospital performance dashboard and individual indicators. | 7 |
| 2841 | O. Groene, N. Klazinga, V. Kazandjian, P. Lombrail, P. Bartels, The World Health Organization Performance Assessment Tool for Quality Improvement in Hospitals (PATH): an analysis of the pilot implementation in 37 hospitals., Int. J. Qual. Health Care. 20 (2008) 155–161. | Belgium, Canada, Denmark, France, Slovakia, South Africa | Hospital | Obstetrics,  Antimicrobial stewardship,  Mortality,  Admission rates,  Staff experience,  Health care structures,  Patient experience | Physicians, nurses, managers | 43 | Similar to Veillard et al. 2013. Hospitals collected data themselves then reported back to WHO. | 2 |
| 2794 | V. Grando, M. Rantz, M. Maas, Nursing home staff’s views on quality improvement interventions: a follow up study, J. Gerontol. Nurs. 33 (2007) 40–47. | US | Nursing home | Nursing - general | nurses, managers | 23 | Two different interventions. Group 1: quarterly reports and educational workshop. Group 2: Same plus on-site / phone support from advanced practice nurses. | 5 |
| 4222 | H. Kristensen, L. Hounsgaard, Evaluating the impact of audits and feedback as methods for implementation of evidence in stroke rehabilitation., Br. J. Occup. Ther. 77 (2014) 251–259. doi:10.4276/030802214X13990455043520. | Denmark | Hospital | Stroke | Occupational therapists | 22 | Quarterly feedback presented both orally and in writing as tables, which were handed out to the participants at each audit session. | 5 |
| 5235 | M.M. Meijers, R.J.G. Halfens, D.M. Mijnarends, H. Mostert, J.M.G.A. Schols, A feedback system to improve the quality of nutritional care., Nutrition. 29 (2013) 1037–1041. | Netherlands | Nursing home | Nutritional care | Nurses, managers | 30 | Computerised feedback programme. Staff collect their own data using objective assessments e.g. patient questionnaires / assessments | 4 |
| S81 | G. Harvey, A. Kitson, Achieving improvement through quality: an evaluation of key factors in the implementation process, J. Adv. Nurs. 24 (1996) 185–195. | England | Hospital | Nursing - general | Nurses | 14 | Three interventions: 1) Monitor - Care processes and structure; Acute medicine and surgery; Peer assessment? Sample of patients; 250 criteria - y/n responses  2) QualPacs - 2 assessors - observe care and audit medical records; 68 criteria 3) DySSy - no pre-defined criteria; 3 stage process of defining quality, measuring, and taking action; continuous quality improvement; small groups of practitioners 4-6 work with trained facilitators. | 5 |
| 5357 | C. Morrell, G. Harvey, A. Kitson, Practitioner based quality improvement: a review of the Royal College of Nursing’s dynamic standard setting system., Qual. Health Care. 6 (1997) 29–34. | England | Hospital | Nursing - general | Nurses | 28 | DySSy – see Harvey and Kitson 1996 | 6 |
| 5857 | C. Palmer, J. Bycroft, K. Healey, A. Field, M. Ghafel, Can formal collaborative methodologies improve quality in primary health care in New Zealand? Insights from the EQUIPPED Auckland Collaborative., J. Prim. Health Care. 4 (2012) 328–336. | New Zealand | Primary care | Chronic care | Physicians Nurses Managers | 15 | BTS (Breakthrough Series) Collaborative. Computerised audit tools, facilitation and protected time to engage with the Feedback intervention. | 3 |
| 7025 | N. Shepherd, T.J. Meehan, F. Davidson, T. Stedman, An evaluation of a benchmarking initiative in extended treatment mental health services., Aust. Health Rev. 34 (2010) 328–333. | Australia | mental health - inpatient | Mental health | Nurses Managers | 84 | Written and face-to-face feedback provided by external team. A State-wide forum helped recipients review their findings, and discuss areas that had the potential for improvement. | 4 |
| 7049 | K. Siddiqi, J. Newell, What were the lessons learned from implementing clinical audit in Latin America?, Clin. Gov. An Int. J. (2009) 21–22. doi:10.1108/14777270910976157. | Bolivia, Peru, Cuba | Primary care | TB diagnosis | Physicians Nurses | 43 | Feedback intervention organised by a committee across different organisations. Results were fed back to the health professionals in each committee meeting. | 8 |
| 7050 | K. Siddiqi, A. Volz, L. Armas, L. Otero, R. Ugaz, E. Ochoa, et al., Could clinical audit improve the diagnosis of pulmonary tuberculosis in Cuba, Peru and Bolivia?., Trop. Med. Int. Health. 13 (2008) 566–578. | Bolivia, Peru, Cuba | Primary care | TB diagnosis | Physicians Nurses | 43 | See Siddiqi and Newell 2009. | 7 |
| 8249 | Z. Paskins, H. John, A. Hassell, I. Rowe, The perceived advantages and disadvantages of regional audit: a qualitative study, Clin. Gov. An Int. J. (2011). | England | Hospital - outpatient | Rheumatology | Physicians, nurses, managers | 7 | Six different audits based on national clinical guidelines conducted locally coordinated by a central committee. Each audit was led by a consultant member of the committee coordinated by a group of trainees and had information technology support to design proformas and collate data from a clinical audit department within the region. Each unit received details of its individual performance with anonymised results from other units | 10 |
| S16 | M.B. Boyce, J.P. Browne, J. Greenhalgh, Surgeon’s experiences of receiving peer benchmarked feedback using patient-reported outcome measures: a qualitative study., Implement. Sci. 9 (2014) 84. doi:10.1186/1748-5908-9-84. | Ireland | Hospital | Orthopaedics | Orthopaedic surgeons | 11 | Patient-reported outcomes fed back as reports showing comparisons to other surgeons. | 9 |
| S17 | J.S. Ross, L. Williams, T.M. Damush, M. Matthias, Physician and other healthcare personnel responses to hospital stroke quality of care performance feedback: a qualitative study, BMJ Qual. Saf. (2015) bmjqs-2015-004197. doi:10.1136/bmjqs-2015-004197. | US | Hospital | Stroke | Managers | 41 | Joint Commission and VHA-specific stroke care quality measures. Reports sent to an Executive Director and Director of Quality at each facility, and each facility was given time to review and make corrections to the final data. Publically released. Data collected and analysed by VHA office of quality and performance; now office of performance measurement. | 7 |
| S19 | A. Taylor, J. Neuburger, K. Walker, D. Cromwell, O. Groene, How is feedback from national clinical audits used? Views from English National Health Service trust audit leads., J. Health Serv. Res. Policy. 21 (2016) 91. doi:10.1177/1355819615612826. | England | Hospital | Cancer | Physicians, nurses, managers | 32 | Four national clinical audits:  National Oesophago-Gastric Cancer Audit (NOGCA), National Bowel Cancer Audit (NBOCAP), National Head and Neck Cancer Audit (DAHNO) and the National Lung Cancer Audit (NLCA). | 6 |
| S33 | C. McFadyen, S. Lankshear, D. Divaris, M. Berry, A. Hunter, J. Srigley, et al., Physician level reporting of surgical and pathology performance indicators: A regional study to assess feasibility and impact on quality, Can. J. Surg. 58 (2015) 31–40. doi:10.1503/cjs.004314. | Canada | Hospital | Cancer surgery | Physicians - surgeons and pathologists | 11 | Data collected by querying a centralized database of cancer pathology synoptic reports. Each specimen in the indicator was validated by the research team. Feedback given as written or emailed electronic documents. | 3 |
| S62 | S. Tierney, R. Kislov, C. Deaton, A qualitative study of a primary-care based intervention to improve the management of patients with heart failure: the dynamic relationship between facilitation and context., BMC Fam. Pract. 15 (2014) 1–10. doi:10.1186/1471-2296-15-153. | England | Primary care | Heart failure | Physicians Nurses | 16 | Feedback intervention supported by external team. Education and training provided. Support doing the audit - case finding and register verification. Feedback provided face-to-face and in electronic documents. | 10 |
| S67 | M. Dixon-Woods, M. Leslie, J. Bion, C. Tarrant, What Counts? An Ethnographic Study of Infection Data Reported to a Patient Safety Program, Milbank Q. 90 (2012) 548–591. doi:10.1111/j.1468-0009.2008.00538.x. | England | Hospital | Intensive care | Physicians Nurses Managers | 122 | Matching Michigan intervention. National quality improvement programme. Education, support, and mentoring provided. Dedicted team within each unit to do Feedback intervention. Units submitted data online. Feedback provided centrally with anonymised comparisons to other units. | 10 |
| S71 | M. Gort, M. Broekhuis, G. Regts, How teams use indicators for quality improvement - a multiple-case study on the use of multiple indicators in multidisciplinary breast cancer teams., Soc. Sci. Med. 96 (2013) 69–77. doi:10.1016/j.socscimed.2013.06.001. | Netherlands | Hospital | Cancer surgery | Surgeons, nurses, radiologists, internal medicine specialists | 22 | External project facilitators to conduct Feedback intervention based on national guidelines.  Data for each indicator were registered in a standardized system All participants were invited to share their experiences and project results during two working conferences. Between and after these conferences, the individual teams could organize additional team meetings to discuss results, progress and any other topic. | 5 |
| S104 | V.L. Payne, S.J. Hysong, Model depicting aspects of audit and feedback that impact physicians’ acceptance of clinical performance feedback, BMC Health Serv. Res. 16 (2016) 260. doi:10.1186/s12913-016-1486-3. | US | Primary care | Chronic care | Physicians | 12 | See Powell et al. 2014 | 7 |
| 3351 | S.J. Hysong, M.K. Knox, P. Haidet, Examining clinical performance feedback in Patient-Aligned Care Teams., J. Gen. Intern. Med. 29 Suppl 2 (2014) S667-74. doi:10.1007/s11606-013-2707-7. | US | Primary care | Chronic care | Physicians, nurses, managers | 48 | See Powell et al. 2014 | 6 |
| S105 | A.C. Eldh, M. Fredriksson, S. Vengberg, C. Halford, L. Wallin, T. Dahlström, et al., Depicting the interplay between organisational tiers in the use of a national quality registry to develop quality of care in Sweden., BMC Health Serv. Res. 15 (2015) 519. doi:10.1186/s12913-015-1188-2. | Sweden | Hospital | Stroke | All hospital staff - clinicians and managers | 44 | National clinical audit. Registry data. Centralised data analysis. Since 1998. National benchmarking. | 2 |
| S132 | A.C. Eldh, M. Fredriksson, C. Halford, L. Wallin, T. Dahlström, S. Vengberg, et al., Facilitators and barriers to applying a national quality registry for quality improvement in stroke care., BMC Health Serv. Res. 14 (2014) 354. doi:10.1186/1472-6963-14-354. | Sweden | Hospital | Stroke | All hospital staff - clinicians and managers | 25 | See Eldh et al. 2015. | 5 |
| S38 | R. McDonald, J. White, T.R. Marmor, Paying for Performance in Primary Medical Care: Learning about and Learning from “Success” and “Failure” in England and California, J. Health Polit. Policy Law. 34 (2009) 747–776. doi:10.1215/03616878-2009-024. | US | Primary care | Chronic care, preventive medicine | Physicians | 20 | Findings based on California intervention only. Feed- back on performance provided by physician organization (IPA or medical group). Linked to pay-for-performance. Derived from mainly paper medical records. Data collected by third parties. | 6 |
| S112 | R. Mcdonald, M. Roland, Pay for Performance in Primary Care in England and California: Comparison of Unintended Consequences, (2009) 121–127. doi:10.1370/afm.946.Confl. | US | Primary care | Chronic care, preventive medicine | Physicians | 20 | See McDonald et al. 2009. | 8 |
| S117 | L.J. Damschroder, C.H. Robinson, J. Francis, D.R. Bentley, S.L. Krein, A.M. Rosland, et al., Effects of Performance Measure Implementation on Clinical Manager and Provider Motivation, J. Gen. Intern. Med. 29 (2014) 877–884. doi:10.1007/s11606-014-3020-9. | US | Primary care | Diabetes | Physicians Nurses Managers | 62 | Automatic data extraction from electronic health records. | 8 |
| S118 | L.M. Chadwick, A. Macphail, J.E. Ibrahim, L. Mcauliffe, S. Koch, Y. Wells, Senior staff perspectives of a quality indicator program in public sector residential aged care services: A qualitative cross-sectional study in Victoria, Australia, Aust. Heal. Rev. 40 (2016) 54–62. doi:10.1071/AH14197. | Australia | Nursing home | Nursing - general | Nurses Managers | 56 | Data collected by recipients and sent centrally. Fed back as written reports quarterly. | 7 |
| S14 | Z. Landis-lewis, R. Manjomo, O.J. Gadabu, M. Kam, B.N. Simwaka, S.L. Zickmund, et al., Barriers to using eHealth data for clinical performance feedback in Malawi : A case study, Int. J. Med. Inform. 84 (2015) 868–875. | Malawi | Hospital - outpatient | HIV/AIDS | Physicians | 32 | Electronic medical record data automatically extracted at clinic level. Quarterly reports generated, also via supervisors face-to-face. | 8 |
| S120 | L.G. de Vos Maartje, S.N. van der Veer, W.C. Graafmans, N.F. de Keizer, K.J. Jager, G.P. Westert, et al., Process evaluation of a tailored multifaceted feedback program to improve the quality of intensive care by using quality indicators., BMJ Qual. Saf. 22 (2013) 233–41. doi:10.1136/bmjqs-2012-001375. | Netherlands | Hospital | Intensive care | Nurses, Physicians | 9 | “InFoQI”. Three elements: 1) Pro- vision of comprehensive monthly and quarterly feed- back reports - written 2) Establishment of a local multidisciplinary QI team and 3) two educational out- reach visits. | 5 |
| S127 | S.G. Yi, N.P. Wray, S.L. Jones, B.L. Bass, J. Nishioka, S. Brann, et al., Surgeon-specific performance reports in general surgery: An observational study of initial implementation and adoption, J. Am. Coll. Surg. 217 (2013) 636–647.e1. doi:10.1016/j.jamcollsurg.2013.04.040. | US | Hospital | General surgery | Colorectal and general surgical surgeons | 23 | Surgeon specific feedback. Data collection and analysis done by nurses. Feedback sent via secure electronic mail. | 5 |
| S25 | M. Lloyd, S. Watmough, S. O’Brien, N. Furlong, K. Hardy, Formalized prescribing error feedback from hospital pharmacists: Physicians’ attitudes and opinions, Br J Hosp Med. 76 (2015) 713–8. | England | Hospital | Prescribing (general) | Physicians | 10 | All prescriptions audited and fed back by pharmacists to individual doctors. | 7 |
| S136 | M. Scholte, C.W.M. Neeleman-van der Steen, P.J. van der Wees, M.W.G. Nijhuis-van der Sanden, J. Braspenning, The Reasons behind the (Non)Use of Feedback Reports for Quality Improvement in Physical Therapy: A Mixed-Method Study, PLoS One. 11 (2016) e0161056. doi:10.1371/journal.pone.0161056. | Netherlands | Primary care | Physiotherapy, organisational management | Physiotherapists | 12 | “Qualiphy”. Nationwide National benchmarking programme. First was a self-rating, then extracted from EHRs. Initially voluntary, then paid for participation by health insurers. | 7 |
| S150 | L. Jeffs, S. Beswick, J. Lo, Y. Lai, A. Chhun, H. Campbell, Insights from staff nurses and managers on unit-specific nursing performance dashboards: a qualitative study., BMJ Qual. Saf. (2014) 1–6. doi:10.1136/bmjqs-2013-002595. | Canada | Hospital | Nursing - general | Nurses | 56 | “Care Utilising Evidence (CUE)” dashboard. Interactive computerised dashboard tool  displaying performance data for each unit’s selected guidelines  on smart board and/or computer workstation. Also printed on bulletin boards and fed back to teams face-to-face by team leaders. | 7 |
| S109 | K. Kirschner, J. Braspenning, J.E.A. Jacobs, R. Grol, Experiences of general practices with a participatory pay-for-performance program: a qualitative study in primary care., Aust. J. Prim. Health. 19 (2013) 102–6. doi:10.1071/PY12032. | Netherlands | Primary care | Chronic care, preventive medicine, practice management | Physicians | 29 | Written feedback provided as part of –ay-for-performance scheme. | 5 |
| S154 | R. a Simms, H. Ping, A. Yelland, A.J. Beringer, R. Fox, T.J. Draycott, Development of maternity dashboards across a UK health region; current practice, continuing problems., Eur. J. Obstet. Gynecol. Reprod. Biol. 170 (2013) 119–24. doi:10.1016/j.ejogrb.2013.06.003. | England | Hospital | Obstetrics | Nurses - midwives | 24 | Ten different “dashboards” across multiple organisations. Data manually collected and analysed by recipients. Dashboards could be paper or computerised. | 5 |
| S158 | L. McLellan, T. Dornan, P. Newton, S.D. Williams, P. Lewis, D. Steinke, et al., Pharmacist-led feedback workshops increase appropriate prescribing of antimicrobials, J. Antimicrob. Chemother. (2016) dkv482. doi:10.1093/jac/dkv482. | England | Hospital | Prescribing (general) | Physicians - junior | 10 | Data collected and analysed by pharmacists from prescription cards. Appropriateness judged by an expert panel. Written feedback and face-to-face given. Workshops involving education and reflection also provided. | 7 |
| S159 | S. Redwood, N.B. Ngwenya, J. Hodson, R.E. Ferner, J.J. Coleman, Effects of a computerized feedback intervention on safety performance by junior Physicians: results from a randomized mixed method study, BMC Med Inf. Decis Mak. 13 (2013) 63. doi:10.1186/1472-6947-13-63. | England | Hospital | Prescribing (general) | Physicians - junior | 19 | Web-based dashboard. Data collected as secondary use from Clinical Decision Support system. Email reminders send with link to dashboard every week. | 8 |
| S175 | L. Jeffs, D. Doran, L. Hayes, C. Mainville, S. VanDeVelde-Coke, L. Lamont, et al., Implementation of the National Nursing Quality Report Initiative in Canada: Insights From Pilot Participants., J. Nurs. Care Qual. 30 (2015) E9-16. doi:10.1097/NCQ.0000000000000122. | Canada | Hospital | Nursing - general | Nurses - leaders | 18 | Data collected by recipients. Central web-based reporting system. Feedback via electronic documents and the web. Organisational and unit-level feedback. Peer workshops provided. | 6 |
| S176 | L. Jeffs, J. Lo, S. Beswick, A. Chuun, Y. Lai, H. Campbell, et al., Enablers and barriers to implementing unit-specific nursing performance dashboards., J. Nurs. Care Qual. 29 (2014) 200–3. doi:10.1097/NCQ.0000000000000064. | Canada | Hospital | Nursing - general | Nurses | 56 | See Jeffs et al. 2014 | 4 |
