# Supplementary material for: Clinical Performance Feedback Intervention Theory (CP-FIT): a new theory for designing, implementing, and evaluating feedback in health care based on a systematic review and meta-synthesis of qualitative research
Source: Implement Sci. 2019 Apr 26;14:40. doi: 10.1186/s13012-019-0883-5 (PMC6486695; doi:10.1186/s13012-019-0883-5)
Supplement: Supplementary file 5 — CP-FIT codebook. (DOCX 248 kb) [file 13012_2019_883_MOESM5_ESM.docx]

**Additional file 5: Clinical Performance Feedback Intervention Theory codebook**

**Introduction**

This additional file presents the codebook developed from our metasynthesis in tables in the following pages. Each code has a definition, references to supporting papers and pre-existing theories, and GRADE-CERQual ratings. Codes with a ‘high’ GRADE-CERQual rating are current constructs of Clinical Performance Feedback Intervention Theory (CP-FIT).

**Annotations**

- *Italics* are used to indicate when codes cross-reference each other.
- Relevant constructs of other pre-existing theories are provided in brackets.
- In general, most predictor variables have direct effects on feedback cycle processes. However, some had indirect effects by influencing other variables first. These are denoted as ‘ripple effects’ [1].
- Some predictor variables often had opposing effects on Feedback Cycle processes, suggesting that simply overcoming a ‘barrier’ or implementing a ‘facilitator’ does not necessarily lead to success [2]. These situations are denoted as ‘effect tensions’.
- Codes with ‘high’ GRADE-CERQual ratings had no or very minor concerns regarding all four criteria (methodological limitations, relevance, coherence, and adequacy) [3]. Reasons explaining the ratings of ‘moderate’ and ‘low’ codes are provided.
- Supporting paper IDs refer to identification numbers given in Additional file 4.

**References**

[1] J. Jagosh, P.L. Bush, J. Salsberg, A.C. Macaulay, T. Greenhalgh, G. Wong, et al., A realist evaluation of community-based participatory research: partnership synergy, trust building and related ripple effects, BMC Public Health. 15 (2015) 725. doi:10.1186/s12889-015-1949-1.

[2] K. Checkland, S. Harrison, M. Marshall, Is the metaphor of “barriers to change” useful in understanding implementation? Evidence from general medical practice., J. Health Serv. Res. Policy. 12 (2007) 95–100. doi:10.1258/135581907780279657.

[3] S. Lewin, C. Glenton, H. Munthe-Kaas, B. Carlsen, C.J. Colvin, M. Gülmezoglu, et al., Using Qualitative Evidence in Decision Making for Health and Social Interventions: An Approach to Assess Confidence in Findings from Qualitative Evidence Syntheses (GRADE-CERQual), PLOS Med. 12 (2015) e1001895. doi:10.1371/journal.pmed.1001895.

**The feedback cycle**

| **Feedback Cycle process** | **Description** | **Evidence** |
| --- | --- | --- |
| *Goal setting (a)* | The clinical topic and its associated clinical behaviours or patient outcomes against which performance will be measured are decided. This usually occurs as a one-off step at the beginning of the process. | Supporting paper IDs (n=8): 7049, 7050, S154 S154, S175 S175, 2841, 4222, 8249, S109  Theories: Goal Setting Theory (Goal)  GRADE-CERQual rating: High |
| *Goal setting (b)* | Goal setting may be re-visited after *Data collection and analysis* has taken place or attempted e.g. see *Conducted by recipients, Automation, Measurability*. *Performance level* is also relevant here if current performance too extreme (either too high to improve further or too low to result in improvement). | Supporting paper IDs (n=5): 2841, 7049, 8249, 4222, 7049  Theories: Goal Setting Theory (Goal), Feedback Intervention Theory (Eliminating Feedback-Standard Gap Strategy – 2. Abandon / 3. Change standard)  GRADE-CERQual rating: Moderate  Reason: Concerns regarding coherence, relevance, and methodological limitations of findings. |
| *Data collection and analysis* | Clinical performance data is collected on a defined population of patients and analysed in accordance with *Goal setting*. | Supporting paper IDs (n=29): 1948, 2841, 5357, 627, 7025, 7301, 8249, S118, S132, S136, S175 S175, S154 S154, S62, S67, 1271, 5857, 627, 7049, 7050, 7816, 8167, 8249, S105, S109, S120, S127, S14, S15, S16, S175, S19, S25,    GRADE-CERQual rating: High |
| *Feedback* | A message regarding the measured clinical performance is generated and communicated to health professionals in whom improvement is desired. | Supporting paper IDs (n=11): S154, 7049, 1948, 2023, 7025, S105, S17, S19, 2794, 5033, S52  Theories: Control Theory (Input), Individual Feedback Theory (Complex feedback stimulus), Feedback Intervention Theory (Feedback intervention), Feedback (Goal Setting Theory)  GRADE-CERQual rating: High |
| *Interaction* | The health professionals receive the feedback message. | Supporting paper IDs (n=45): S176, S175 S175, S154 S154, 1271, 1591, 1948, 2794, 2841, 3351, 512, 5357, 5857, 6087, 7025, 7049, 7816, S105, S109, S118, S127, S132, S136, S14, S15, S175, S176, S19, S25, S30, S32, S4, S52, S62, S71, S81, 2794, 2857, 5033, 7194, 7694, 7783, S1, S104, S120, S150, S154, S17, S6, S67  Theories: Information Value Chain (Interaction)  GRADE-CERQual rating: High |
| *Perception* | The health professionals interpret the feedback message. This does not have to be an accurate interpretation. | Supporting paper IDs (n=35): S176 S176, S175 S175, S158 S158, 1948, 2794, 2841, 2857, 4222, 5033, 5235, 5857, 627, 7194, 7694, 7783, S1, S105, S118, S120, S127, S132, S136, S15, S150, S154, S158, S16, S19, S25, S30, S38, S4, S6, S62, S7, S71  Theories: Control Theory (Comparator), Individual Feedback Theory  GRADE-CERQual rating: High |
| *Acceptance* | The health professionals believe the feedback message is an accurate portrayal of their performance. This belief does not necessarily have to be correct. They become aware of their measured level of performance. | Supporting paper IDs (n=58): S175 S175, S159 S159, S158 S158, 1271, 1591, 187, 2023, 2249, 2794, 2841, 2857, 4222, 5033, 512, 5357, 5532, 5857, 6087, 7025, 7049, 7050, 7194, 7301, 7694, 7783, 7816, 8167, 8249, S1, S104, S105, S109, S112, S118, S120, S127, S132, S136, S14, S15, S150, S16, S17, S19, S25, S28, S30, S32, S33, S38, S4, S52, S6, S62, S67, S7, S71, S81.  Theories: Individual Feedback Theory, Control Theory, Feedback Intervention Theory (Eliminating Feedback-Standard Gap Strategy – 4. Reject feedback)  GRADE-CERQual rating: High |
| *Verification* | Health professionals interrogate the feedback message or the underlying clinical performance data in an attempt to improve their *Perception* of the feedback message prior to its *Acceptance*. | Supporting paper IDs (n=13): 1591, 2023, 2794, 5033, 7194, 7694, S1, S104, S118, S127, S38, S6    GRADE-CERQual rating: High |
| *Intention* | The health professionals make a conscious decision to behave in a certain way in response to the feedback message. | Supporting paper IDs (n=52): S176 S176, S175 S175, S159 S159, S158 S158, 1271, 187, 1948, 2023, 2249, 2857, 4222, 5033, 5235, 5357, 5857, 627, 7025, 7049, 7050, 7194, 7301, 7694, 7783, 7816, 8167, 8249, S1, S104, S105, S109, S117, S118, S120, S127, S132, S136, S15, S150, S16, S17, S19, S25, S28, S30, S32, S33, S38, S52, S6, S62, S67, S7, S71  Theories: Individual Feedback Theory, Theory of Planned Behaviour  GRADE-CERQual rating: High |
| *Behaviour* | The health professionals behave in a way consistent with their conscious decision (intentions). This may be an increase, decrease, change, or maintenance of Data collection and analysised behaviour. | Supporting paper IDs (n=58): 1271, 1591, 187, 1948, 2023, 2794, 2841, 2857, 3351, 4222, 5033, 512, 5235, 5357, 5532, 5857, 627, 7025, 7049, 7050, 7194, 7301, 7694, 7783, 7816, 8167, 8249, S1, S104, S105, S112, S117, S118, S120, S127, S132, S136, S14, S15, S158, S159, S16, S17, S175, S19, S25, S28, S30, S32, S38, S4, S52, S6, S62, S67, S7, S71, S81.  Theories: Individual Feedback Theory, Control Theory (Output), Feedback Intervention Theory (Eliminating Feedback-Standard Gap Strategy – 1. Change behaviour), Goal setting theory; Multilevel approach to change**;** Theory of Planned Behaviour  GRADE-CERQual rating: High |
| *Clinical performance improvement* | Clinical performance as measured by the feedback intervention may improve, worsen, or remain the same. | Supporting paper IDs (n=39): 1591, 187, 1948, 2023, 2794, 2857, 3351, 4222, 512, 5357, 5532, 5857, 6087, 7049, 7050, 7301, 7816, 8167, S1, S104, S117, S118, S132, S15, S154, S158, S159, S16, S19, S25, S30, S38, S4, S52, S6, S62, S67, S71, S81.  Theories: Individual Feedback Theory, Control Theory (Impact on environment)  GRADE-CERQual rating: High |
| **Potential unintended outcomes – positive** | | |
| *Improved record keeping* | Feedback interventions may lead to improved record keeping by recipients addressing suboptimal clinical performance measured due to poor record keeping. This may impact on feedback *Accuracy*. | Supporting paper IDs (n=8): 187, 2023, 2794, 7049, 7816, S132, S32, S6  GRADE-CERQual rating: High |
| *Improved knowledge and awareness* | Feedback interventions may increase recipients’ knowledge and awareness of the clinical topic addressed by the feedback intervention by reminding (e.g. S52, S6) or informing (e.g. S62, S30) them of important aspects of clinical performance they did or did not already know, respectively. This may impact on recipients’ *Knowledge and skills in clinical topic*. | Supporting paper IDs (n=20): 1591, 187, 2023, 4222, 5532, 627, 7049, 7050, S1, S105, S118, S127, S132, S158, S25, S30, S4, S52, S6, S62  GRADE-CERQual rating: High |
| *Patient engagement* | Patients may become more engaged in their care. | Supporting paper IDs (n=2): S4, S62  GRADE-CERQual rating: Low  Reason: Serious concerns regarding adequacy of findings. |
| **Potential unintended outcomes – negative** | | |
| *Gaming* | In response to feedback interventions, health professionals may unethically manipulate clinical data (e.g. record a care process has happened when it has not 1948) or change their patient population (e.g. a surgeon may choose not to operate on high-risk patients S127) in order to artificially improve their measured clinical performance). | Supporting paper IDs (n=9): 1948, 8167, S109, S112, S127, S136, S16, S38, S67.  Theories: Cognitive dissonance, self-affirmation  GRADE-CERQual rating: Moderate  Reason: Moderate concerns regarding coherence and adequacy of findings. |
| *Tunnel vision* | In response to feedback intervention, health professionals may become overly focused on the topic against which clinical performance is measured, to the detriment of other clinical areas. This may manifest during the care of individual patients (e.g. 6087) or during quality improvement activities (e.g. 2857). | Supporting paper IDs (n=6): 1948, 2857, 512, 6087, S109, S117, S67  Theories: Cognitive dissonance, self-affirmation  GRADE-CERQual rating: Moderate  Reason: Serious concerns regarding adequacy of findings. |
| *Inappropriate care* | Patients may receive care that is either unnecessary or for which they have not provided consent in order to improve measured clinical performance. | Supporting paper IDs (n=2): 6087, S112  Theories: Cognitive dissonance, self affirmation  GRADE-CERQual rating: Low  Reason: Serious concerns regarding methodological limitations and adequacy of findings. |

**Feedback variables**

| **Name** | **Description, effect hypothesis, and mechanism** | **Evidence** |
| --- | --- | --- |
| ***Goal*** | | |
| *Importance* | Description: The perceived clinical importance of the topic within the feedback message according to the recipient. The importance of a topic can relate to whether it: 1) represents ‘good’ clinical care, 2) impacts patient outcomes, 3) is comprehensive (i.e. measures all relevant aspects of clinical care), and 4) addresses a perceived quality problem (i.e. an area in which performance is suboptimal). | Supporting paper IDs (n=28): 1591, 187, 1948, 2023, 2249, 2794, 2857, 4222, 5033, 512, 5857, 6087, 7049, 7050, 7194, 7694, 7816, 8167, 8249, S1, S127, S16, S28, S30, S32, S38, S6, S7 |
|  | Effect hypothesis and mechanism (1): Increased importance facilitates *Interaction, Acceptance, Intention,* and *Behaviour* by increasing *Credibility* (because the recipient believes it is an important topic), *Compatibility* (with recipients’ views on what is clinically significant) and *Relative advantage* (as they may not receive information about important topics from elsewhere)*.* | Supporting paper IDs (n=21): 1591, 187, 2023, 2249, 2794, 2857, 4222, 512, 5857, 6087, 7049, 7050, 7194, 7694, 7816, 8167, S127, S16, S28, S30, S32, S6, S7  Theories: Goal Setting Theory (Goal commitment); Self-determination Theory (Intrinsic motivation); COM-B System (Motivation); Motivation-Opportunities-Abilities Model (Motivation); Theory of Planned Behaviour (Attitude)  GRADE-CERQual rating: High |
| *Controllability* | Description: The degree to which the topic of the feedback message, and any changes that need to be made in response to suboptimal performance, is perceived to be within the control of the recipient. | Supporting paper IDs (n=23): 1271, 187, 2249, 5532, 7025, 7301, 8167, S1, S104, S112, S117, S118, S120, S127, S132, S136, S154, S16, S25, S30, S52, S6, S71 |
|  | Effect hypothesis and mechanism (1): Increased controllability facilitates *Acceptance, Intention, Behaviour,* and *Clinical performance improvement* by increasing *Actionability.* | Supporting paper IDs (n=18): 1271, 187, 2249, 5532, 7025, 8167, S104, S117, S118, S120, S127, S132, S136, S154, S16, S25, S30, S52, S71  Theories: Goal Setting Theory (Self-efficacy); Individual Feedback Theory (Locus of control); Locus of Control theory; Self-Efficacy Theory; Theory of Planned Behaviour (Perceived behavioural control)  GRADE-CERQual rating: High |
| *Relevance* | Description: The relevance of the feedback message topic to the recipients’ job. | Supporting paper IDs (n=8): 7301, S120, S132, S136, S25, S52, S6, S71 |
|  | Effect hypothesis and mechanism (1): Increased relevance facilitates *Data collection and analysis* (when *Conducted by recipients*)*, Acceptance, Intention,* and *Behaviour* by increasing *Compatibility* (with a recipients’ job) and therefore also *Actionability,* and *Relative advantage.* | Supporting paper IDs (n=8): 7301, S120, S132, S136, S25, S52, S6, S71  Theories: Diffusion of Innovations (Task relevance); Technology Acceptance Model (Job relevance)  GRADE-CERQual rating: High |
| *Evidence base* | Description: The evidence base and rationale supporting the goal against which clinical performance is measured. This may be derived from clinical guidelines (e.g. S105), research studies (e.g. S120), or expert opinion (e.g. S38). | Supporting paper IDs (n=20): 1271, 2023, 2249, 7049, 7050, 7816, 8167, S1. S105, S112, S117, S120, S132, S14, S30, S38, S4, S6, S7, S71 |
|  | Effect hypothesis and mechanism (1): A strong evidence base facilitates *Acceptance* by increasing *Credibility* and *Compatibility* with recipients’ *motivation* (ensuring they are providing evidence-based care). | Supporting paper IDs (n=15): 1271, 2023, 2249, 7050, 7816, 8167, S1, S105, S120, S132, S14, S30, S4, S6, S7  Theories: Feedback Intervention Theory (Norms)GRADE-CERQual rating: Moderate  Reason: Minor concerns regarding the coherence of findings (some papers reported recipients not agreeing with evidence-based measures i.e. *Compatibility* 1271, 2249, S1). |
| *Process vs outcome* | Description: Whether clinical performance is measured regarding care processes (e.g. 5033) or patient outcomes (e.g. S127). Can correlate with *Accuracy* (and *Benchmarking*), as outcome measures are often perceived as inaccurate when comparing health professionals if not analysed with sufficient casemix adjustment, and *Controllability* as often process measures are perceived to be within health professionals’ control. | Supporting paper IDs (n=12): 1591, 5033, 5532, 5857, 8167, S120, S127, S16, S30, S32, S7, S71 |
|  | Effect hypothesis and mechanism (1a – effect tension): Process measures facilitate *Perception, Acceptance, Intention, Behaviour (patient-level),* and *Performance improvement* by decreasing *Complexity* (the consequence of process measures are easier to understand, whereas outcome measures often require further statistical analysis e.g. S120), and increasing *Actionability* (process measures are easier to influence) and *Credibility* (the attribution of process measures to a health professional’s clinical performance are more believable). | Supporting paper IDs (n=10): S120, S127, S16, S30, S32, S71, 5033, S7, 5532, 1591  GRADE-CERQual rating: Moderate  Reason: Minor concerns regarding adequacy and coherence of findings |
|  | Effect hypothesis and mechanism (1b – effect tension): Process measures inhibit *Intention,* and *Behaviour* because they decrease *Compatibility* and *Credibility* because they may not improve patient outcomes. | Supporting paper IDs (n=10): S120, S127, S16, S30, S32, S71, 5033, S7, 5532, 1591  GRADE-CERQual rating: Moderate  Reason: Minor concerns regarding adequacy and coherence of findings |
|  | Effect hypothesis and mechanism (2): Outcome measures lead to *Gaming* (unintended consequence) by encouraging health professionals to increase *Actionability* via other means. | Supporting paper IDs (n=2): 8167, S127  GRADE-CERQual rating: Low  Reason: Serious concerns regarding adequacy of findings. |
| ***Data collection and analysis method*** | | |
| *Accuracy* | Description: The perceived accuracy of measured clinical performance. This may relate to: 1) the nature of source data, 2) the method of analysis, and/or 3) sample size. Often related to the positive predictive value of the feedback. | Supporting paper IDs (n=41): 1271, 1591, 187, 1948, 2023, 2249, 2794, 2841, 5033, 6087, 7049, 7194, 7301, 7694, 7783, 7816, 8249, S1, S104, S109, S112, S118, S120, S127, S132, S136, S14, S154, S159, S16, S17, S19, S28, S30, S38, S6, S67, S7, S71, S81 |
|  | Effect hypothesis and mechanism (1): Greater Accuracy facilitates Acceptance, Intention, and Behaviour, by increasing *Credibility* (by more accurately reflecting recipients’ clinical performance), and *Relative advantage* (particularly if there is no existing feedback intervention against which to compare, or if an existing feedback intervention is less accurate). | Supporting paper IDs (n=40): 1271, 1591, 187, 1948, 2023, 2249, 2794, 2841, 5033, 6087, 7049, 7194, 7301, 7694, 7783, 7816, 8249, S1, S104, S105, S109, S112, S118, S120, S127, S132, S136, S14, S154, S159, S16, S17, S19, S28, S30, S38, S6, S67, S7, S71, S81  Theories: Individual Feedback Theory (Credibility)  GRADE-CERQual rating: High |
|  | Effect hypothesis and mechanism (2): Lower *Accuracy* facilitates *Verification* because there is reduced *Credibility* of the feedback message and recipients are motivated to check it. | Supporting paper IDs (n=5): 1591, 5033, 7694, S38, S6  GRADE-CERQual rating: Moderate  Reasons: Moderate concerns regarding the adequacy and coherence of findings. |
| *Exclusions* | Description: Recipients can exclude patients they deem unsuitable to be included in the measurement of their clinical performance. For example if there is *Choice alignment* (e.g. if the patient refuses care S112) or if it is *Clinically inappropriate* (e.g. due to existing comorbidities e.g. S6). | Supporting paper IDs (n=15): 1591, 2023, 2794, 4222, 6087, 7694, S1, S104, S112, S117, S118, S159, S30, S38, S6, |
|  | Effect hypothesis and mechanism (1): The inability to exclude patients inhibits *Acceptance* by decreasing *Credibility* (by less accurately reflecting recipients’ clinical performance), *Actionability* (by preventing recipients focusing on patients over which they can improve upon), *Relative advantage* (as most feedback interventions do not provide this feature), and *Compatibility* (by preventing health professionals from judiciously/autonomously applying scientific evidence, and providing patient-centred care, whilst still achieving high levels of clinical performance). | Supporting paper IDs (n=9): 2023, 2794, 4222, 7694, S1, S104, S112, S38, S6  Theories: Cognitive dissonance  GRADE-CERQual rating: High |
|  | Effect hypothesis and mechanism (2): The inability to exclude patients negative Emotions (frustration). | Supporting paper IDs (n=3): S112, S117, S38  GRADE-CERQual rating: Low  Reasons: Serious concerns regarding the adequacy of findings. |
| *Conducted by recipients* | Description: Clinical performance data are collected and/or analysed by the recipients of the feedback intervention. | Supporting paper IDs (n=26): 1271, 1591, 187, 2841, 5357, 5857, 6087, 627, 7025, 7301, 7816, 8249, S105, S109, S112, S118, S120, S127, S132, S15, S154, S16, S175, S19, S67, S81 |
|  | Effect hypothesis and mechanism (1): Inhibits Data collection and analysis by decreasing *Resource match* and *Relative advantage,* and increasing *Complexity*. | Supporting paper IDs (n=21): 1271, 2841, 5357, 5857, 627, 7025, 7301, 7816, 8249, S105, S109, S118, S120, S127, S132, S15, S154, S16, S175, S19, S67  GRADE-CERQual rating: High |
|  | Effect hypothesis and mechanism (2): Increases *Cost* by decreasing *Resource match* (ripple effect). | Supporting paper IDs (n=21): 1271, 2841, 5357, 5857, 627, 7025, 7301, 7816, 8249, S105, S109, S118, S120, S127, S132, S15, S154, S16, S175, S19, S67  GRADE-CERQual rating: High |
|  | Effect hypothesis and mechanism (3): Improves *Teamwork* (ripple effect) by engaging recipients in a common goal. | Supporting paper IDs (n=3): 5357, 7025, S118  GRADE-CERQual rating: Low  Reason: Moderate concerns regarding coherence, adequacy, and methodological limitations of findings. |
|  | Effect hypothesis and mechanism (4a – effect tension): Increases Data collection and analysis *Accuracy* (ripple effect). | Supporting paper IDs (n=4): 7816, S132, S19, S67  GRADE-CERQual rating: Low  Reason: Moderate concerns regarding coherence, adequacy, and methodological limitations of findings. |
|  | Effect hypothesis and mechanism (4b – effect tension): Decreases Data collection and analysis *Accuracy* (ripple effect). | Supporting paper IDs (n=4): 7816, S132, S19, S67  GRADE-CERQual rating: Low  Reason: Moderate concerns regarding coherence, adequacy, and methodological limitations of findings. |
|  | Effect hypothesis and mechanism (5): Increases *Ownership* of the feedback intervention (ripple effect). | Supporting paper IDs (n=2): 627, S118  GRADE-CERQual rating: Low  Reason: Moderate concerns regarding coherence, adequacy, and methodological limitations of findings. |
| *Automation* | Description: Whether clinical performance data are collected regarding individual patients by hand (either using paper e.g. S154 or electronic health records e.g. S109; Manual) rather than automatically (e.g. 1948; Automatic ). | Supporting paper IDs (n=16): 1591, 1948, 5857, 7049, 7050, 7816, S105, S109, S132, S136, S14, S154, S16, S175, S6, S62 |
|  | Effect hypothesis and mechanism (1): Automation facilitates *Data collection and analysis* by increasing *Resource match* and *Relative advantage*, and decreasing *Complexity*. | Supporting paper IDs (n=11): 1948, 5857, 7049, 7050, 7816, S109, S136, S14, S16, S175, S62  GRADE-CERQual rating: High |
| *Measurability* | Description: Whether it is perceived as possible to efficiently measure the *Goal* of the feedback intervention. | Supporting paper IDs (n=3): 2841, 8249, 7049 |
|  | Effect hypothesis and mechanism (1): Lower measurability inhibits *Data collection and analysis* by increasing *Complexity* and decreasing *Resource match.* | Supporting paper IDs (n=3): 2841, 8249, 7049  GRADE-CERQual rating: Low  Reason: Moderate concerns regarding coherence, adequacy, and methodological limitations of findings. |
| *Electronic health record* | Description: Clinical performance data are collected from electronic health records. | Supporting paper IDs (n=9): 2249, S1, S105, S112, S132, S136, S14, S38, S6 |
|  | Effect hypothesis and mechanism: No clear effect (not enough data to draw a conclusion). Possible confounder for *Accuracy*, and *Automation*. | N/A |
| *Number of data sources* | Description: Number of sources of clinical data used to calculate clinical performance. | Supporting paper IDs (n=4): 2841, 8249, S154, S175 |
|  | Effect hypothesis and mechanism: Inhibits Data collection and analysis by increasing Complexity (more data sources is more complex), and decreasing Compatibility (more data sources are less likely to ‘fit’ with existing data systems) and Resource match (more data sources require more resource). | Supporting paper IDs (n=3): 8249, S154, S175  GRADE-CERQual rating: Serious concerns regarding adequacy of findings |
| *Patient-reported* | Description: The data source includes patient-reported e.g. patient-reported outcome measures (PROMs) or experience measures (PREMs). May correlate with *Accuracy* as some recipients may view patient-reported data as unreliable. | Supporting paper IDs (n=6): S136, S15, S16, S28, S30, S7 |
|  | Effect hypothesis and mechanism (1a – effect tension): Facilitates Acceptance and Intention by increasing Compatibility (with recipients’ motivations to provide patient-centred care). | Supporting paper IDs (n=6): S136, S15, S16, S28, S30, S7  GRADE-CERQual rating: Moderate  Reason: Moderate concerns regarding the coherence of findings |
|  | Effect hypothesis and mechanism (1b – effect tension): Inhibits Acceptance and Intention by decreasing Credibility (as some recipients may view patient-reported data as unreliable). |  |
| ***Feedback display*** | | |
| *Benchmarking* | Description: Compares recipients’ clinical performance with other health professionals. Other health professionals may include those internal or external to their organisation, and the comparison may be anonymous or identifiable. | Supporting paper IDs (n=34): 1271, 1948, 2794, 2841, 2857, 3351, 5033, 512, 5857, 7025, 7194, 7783, 7816, 8167, 8249, S104, S105, S109, S118, S120, S127, S132, S158, S16, S17, S175, S19, S25, S32, S33, S4, S6, S67, S7 |
|  | Effect hypothesis and mechanism (1): Facilitates *Perception*, *Intention* and *Behaviour* via reducing *Complexity* (enabling comparison of with others enables recipients to better understand how well they are performing, and which areas require improvement) and increasing *Social influence* (by harnessing competition between recipients [social comparison theory], changing recipients’ behaviour if they see others behaving differently [Persuasion Theory – Social proof; Social Norms Theory – Descriptive norms], and trying to maintain their status in a group of high performing clinicians [Reference group theory]). | Supporting paper IDs (n=12): 1948, 2857, 5033, 5857, 7783, 8249, S104, S105, S118, S120, S127, S132, S175, S17, S19, S25, S32, S6, S7  Theories: Social Comparison Theory; Persuasion Theory (Social proof); Social Norms Theory (Descriptive norms); Reference Group Theory  [Disagrees with Feedback Intervention Theory (Normative information)]  GRADE-CERQual rating: High |
|  | Effect hypothesis and mechanism (2): Facilitates *Organisation-level* behaviour (ripple) by increasing *Actionability* (by providing insight into how other organisations’ performance compares). | Supporting paper IDs (n=2): 5033, S19  GRADE-CERQual rating: Low  Reasons: Serious concerns regarding adequacy and relevance of findings. |
|  | Effect hypothesis and mechanism (3): Induces both positive and negative *Emotions* (ripple effect) dependent on whether relative *Performance* *level* is high or low respectively via *Social influence* (by increasing competition [social comparison theory]) and *Compatibility* with recipients’ expectations. | Supporting paper IDs (n=6): 512, 7025, S104, S158, S17, S4  GRADE-CERQual rating: Moderate  Reasons: Moderate concerns regarding adequacy of findings. |
|  | Effect hypothesis and mechanism (4): Inhibits *Acceptance* (effect tension) by decreasing *Credibility* (when comparisons between health professionals may be perceived as invalid, irrelevant or unfair e.g. without statistical adjustment in outcome measures or when there are considered other clear differences between health professionals). May correlate with *Accuracy*. | GRADE-CERQual rating: Moderate  Reasons: Minor concerns regarding adequacy of findings.  Supporting paper IDs (n=4): 7194, S127, S16, S17, S67 |
|  | Effect hypothesis and mechanism (5): Inhibits *Acceptance* (effect tension) by decreasing *Compatibility* and *Credibility* (some feedback intervention recipients believe comparing health professional’s performance is inappropriate and a threat to their autonomy). | Supporting paper IDs (n=3): 1271, 2857, 7194  GRADE-CERQual rating: Low  Reasons: Serious concerns regarding coherence and adequacy of findings. |
|  | Effect hypothesis and mechanism (6): Facilitates *Interaction* by increasing Relative advantage (the ability to view other health professionals’ clinical performance information is not generally available) and Social influence (by increasing competition [social comparison theory]). | Supporting paper IDs (n=4): 7816, S118, S19, S32  GRADE-CERQual rating: Low  Reasons: Serious concerns regarding coherence and adequacy of findings. |
|  | Effect hypothesis and mechanism (7): Increases *Resource* (ripple effect) by enabling comparisons to other health organisations to persuade management to fund organisational change (*Social influence*). | Supporting paper IDs (n=3): 5033, S19, S25  GRADE-CERQual rating: Low  Reasons: Serious concerns regarding adequacy and relevance of findings. |
| *Framing* | Description: Whether recipients’ clinical performance is presented to emphasise their achievements (positive framing) or shortfalls (negative framing), independent of their actual level of performance (see *Performance level*). May correlate with *Function* and *Non-financial reward,* as they often co-exist. | Supporting paper IDs (n=6): 1271, S104, S117, S62, S67, S81 |
|  | Effect hypothesis and mechanism (1): Positive framing facilitates *Acceptance* by increasing *Compatibility* with recipients’ *professional role*, *motivation*, and *expectations* (as health professionals generally intend to and believe they provide a good standard of care). | Paper (n=3): S104, S62, S81  Theories: Individual Feedback Theory (Sign) [Disagrees with Feedback Intervention Theory (Sign / Praise)]  GRADE-CERQual rating: Low  Reason: Serious concerns regarding coherence and adequacy of findings. |
|  | Effect hypothesis and mechanism (2): Positive framing causes positive *Emotions* (ripple effect) by increasing *Compatibility* with recipients’ *professional role*, *motivation*, and *expectations* (as health professionals generally intend to and believe they provide a good standard of care). | Paper (n=3): 1271, S104, S117  Theories: Individual Feedback Theory (Sign), Feedback Intervention Theory (Sign / Praise)  GRADE-CERQual rating: Low  Reason: Serious concerns regarding coherence and adequacy of findings. |
| *Graphical elements* | Description: Clinical performance is presented using graphical elements such as icons (e.g. 3351), use of colours (e.g. S117), dials, bar charts or line charts. May correlate with *Trend,* as they can co-exist. | Supporting paper IDs (n=8): 3351, 7025, 7194, S117, S127, S150, S154, S62 |
|  | Effect hypothesis and mechanism (1): Facilitates *Perception* by reducing *Complexity* (making the feedback message simpler to understand). | Supporting paper IDs (n=5): S127, S154, S62, 7025, S150  Theories: Feedback Intervention Theory (Graphical elements)  GRADE-CERQual rating: Low  Reason: Serious concerns regarding adequacy of findings. |
| *Number of metrics* | Description: The number of quality indicators in the feedback message that summarise clinical performance. | Supporting paper IDs (n=17): 1271, 2794, 6087, 7194, 7816, S1, S104, S105, S109, S112, S117, S127, S132, S154, S17, S38, S4 |
|  | Effect hypothesis and mechanism (1): Increased number of metrics inhibits *Perception* and *Intention* by increasing *Complexity* (making the feedback message as a whole more difficult to understand and plan to address). | Supporting paper IDs (n=7): 1271, S1, S105, S112, S17, S127, S154, S38  GRADE-CERQual rating: Moderate  Reason: Minor concerns regarding adequacy of findings. |
|  | Effect hypothesis and mechanism (2a – effect tension): Increased number metrics may facilitate *Tunnel vision* (unintended consequence) by decreasing *Resource match* (leaving less time to address patients’ concerns). | Supporting paper IDs (n=2): 6087, S109  GRADE-CERQual rating: Low  Reason: Serious concerns regarding the coherence, adequacy, relevance, and methodological limitations of findings. |
|  | Effect hypothesis and mechanism (2b – effect tension): Increased number metrics may reduce *Tunnel vision* (unintended consequence) by increasing *Importance* via *Compatibility* (ensuring the metrics comprehensively cover what is considered to be important aspects of clinical care). | Supporting paper IDs (n=2): 6087, S109  GRADE-CERQual rating: Low  Reason: Serious concerns regarding the coherence, adequacy, relevance, and methodological limitations of findings. |
| *Patient lists* | Description: The feedback message provides lists of patients included in the calculation of clinical performance. This may include those patients (not) receiving desired care processes (e.g. 1591, 2857), or those who have experienced a particular outcome of interest (e.g. 5532). Correlates with *Detailed patient-level information,* as they often co-exist. | Supporting paper IDs (n=14): 1591, 187, 2857, 3351, 5532, 6087, 7194, S1, S104, S112, S117, S30, S32, S38 |
|  | Effect hypothesis and mechanism (1): Facilitates *Verification* and *Acceptance* by increasing *Credibility* (demonstrating to recipients how their clinical performance was calculated). | Supporting paper IDs (n=10): 1591, 187, 5532, 6087, S104, S112, S32, S38, S1, 7194  Theories: Individual Feedback Theory (Supporting data)  GRADE-CERQual rating: High |
|  | Effect hypothesis and mechanism (2): Facilitates *Perception, Intention* and *Behaviour (patient-level)* by increasing *Actionability* (by providing the identity of patients who may have received poor care, so that it can be corrected or lessons learned) and decreasing *Complexity* (enabling recipients to easily understand how their clinical performance may be suboptimal by reviewing the care of individual patients). | Supporting paper IDs (n=14): 2857, 7194, S1, S117, S38, 1591, 187, 3351, 5532, S112, S32, S38, S104, S117  GRADE-CERQual rating: High |
| *Performance level* | Description: The level of clinical performance communicated by the feedback message perceived by the recipient – i.e. whether the performance level is considered high (performing well) or low (performing badly). | Supporting paper IDs (n=40): 1271, 187, 1948, 2023, 2794, 2857, 3351, 4222, 5033, 512, 5857, 7025, 7049, 7050, 7194, 7301, 8167, S1, S104, S109, S117, S118, S127, S132, S136, S150, S158, S16, S17, S19, S28, S30, S32, S38, S4, S52, S6, S67, S7, S71 |
|  | Effect hypothesis and mechanism (1): Low performance level facilitates *Verification* by decreasing *Compatibility* (as health professionals often believe they provide good care, so they are motivated to check the data). | Supporting paper IDs (n=5): S104, S118, S127, S30, S6  Theories: Feedback Intervention Theory (Sign, Eliminating Feedback-Standard Gap Strategies), Cognitive dissonance  GRADE-CERQual rating: High |
|  | Effect hypothesis and mechanism (2a – effect tension): Low/high performance level facilitates/inhibits (respectively) *Intention, Behaviour* and *Performance improvement* by increasing/decreasing *Compatibility* (as improving clinical performance aligns with their motivations [self-affirmation, cognitive dissonance, control theory, feedback intervention theory – Eliminating Feedback-Standard Gap Strategies, goal setting theory, and Individual Feedback Theory]), and increasing/decreasing *Actionability* (low performance implies there is room for improvement). | Supporting paper IDs (n=29): 1271, 1948, 2794, 2857, 3351, 5033, 5857, 7049, 7050, 7194, 7301, 8167, S104, S109, S132, S136, S150, S16, S17, S19, S127, S28, S30, S32, S38, S52, S6, S67, S71  Theories: Control Theory, Individual Feedback Theory (Sign), Feedback Intervention Theory (Sign, Eliminating Feedback-Standard Gap Strategies), Goal Setting Theory, COM-B System (Motivation, Opportunity); Motivation-Opportunities-Abilities Model (Motivation, Opportunity), Self-affirmation, Cognitive dissonance  GRADE-CERQual rating: High |
|  | Effect hypothesis and mechanism (2b – effect tension): Low performance level inhibits *Acceptance* by decreasing *Compatibility* with expectations (as health professionals often believe they provide good care [self-affirmation theory, cognitive dissonance theory, feedback intervention theory - Eliminating Feedback-Standard Gap strategies, Self-determination theory]) and/or *Resource match* (as they do not have the resources – time, skills, money – to spend on increasing performance). | Supporting paper IDs (n=17): 187, 2794, 4222, 5857, 7049, 7194, 7301, 8167, S1, S104, S132, S158, S16, S17, S19, S28, S30, S7  Theories: Self-affirmation theory, cognitive dissonance theory, feedback intervention theory (Eliminating Feedback-Standard Gap strategies), Self-determination theory (Competence)  GRADE-CERQual rating: Moderate  Reason: Moderate concerns regarding coherence of findings – often non-acceptance may be explained by other variables. |
|  | Effect hypothesis and mechanism (3): Low/high performance level causes negative/positive *Emotions* (ripple effect) (e.g. disappointment/happiness) by decreasing/increasing *Compatibility* with their expectations (as health professionals often believe they provide good care). | Supporting paper IDs (n=18): 1271, 187, 2794, 512, 7025, S1, S104, S109, S117, S127, S132, S150, S158, S16, S17, S19, S28, S4  Theories: Cognitive dissonance  GRADE-CERQual rating: High |
| *Prioritisation* | Description: The feedback message includes design features that effectively summarise and communicate the relative importance of its contents e.g. patients most at risk S1, or highlighting areas of clinical performance that require most urgent attention S127. | Supporting paper IDs (n=11): 2857, 3351, 5033, 7194, S1, S105, S127, S136, S30, S38, S6 |
|  | Effect hypothesis and mechanism (1): Its absence inhibits *Perception, Intention* and *Behaviour* by increasing *Complexity* (making the feedback message more difficult to interpret and decide the most important aspects on which to focus), and decreasing *Actionability* (by making it unclear which aspects of clinical performance require urgent attention) and decreasing *Relative advantage* (as most existing health information systems do not prioritise information to users). | Supporting paper IDs (n=8): S1, S105, S127, S136, S30, S38, S6, 3351, 7194  Theories: Cognitive Load Theory  GRADE-CERQual rating: High |
| *Specificity* | Description: The degree to which the feedback message presents the clinical performance of an individual clinician (e.g. 1948) versus their wider team (e.g. S7) or organisation (e.g. S19). | Supporting paper IDs (n=13): 1948, 3351, 5033, 512, 5532, S104, S127, S158, S19, S30, S4, S6, S7 |
|  | Effect hypothesis and mechanism (1): Increased specificity facilitates *Acceptance, Perception, Intention, Behaviour* and *Performance improvement* by increasing *Actionability* (because the feedback message refers directly to the recipients’ behaviour over which they have most control)*,* and *Relative advantage*. | Supporting paper IDs (n=11): 1948, 3351, 512, 5532, S19, S104, S30, S158, S19, S6, S7  Theories: Individual Feedback Theory (Feedback specificity); [Disagrees with Feedback Intervention Theory (Group feedback)]  GRADE-CERQual rating: High |
|  | Effect hypothesis and mechanism (2): Increased specificity facilitates *Patient-level* behaviour (ripple effect) by increasing *Actionability* (because the feedback message refers directly to the recipients’ behaviour over which they have most control) for individual patients the recipient cares for, rather than the wider organisation. | Supporting paper IDs (n=4): 3351, 512, 5532, S7  GRADE-CERQual rating: Moderate  Reason: Moderate concerns regarding the adequacy of findings. |
|  | Effect hypothesis and mechanism (3): Improves *Teamwork* (ripple effect) by using *Social influence* (Reference group) to enable staff members within an organisation to understand the their specific role in the delivery of care. | Paper (n=3): 3351, 5532, S7  GRADE-CERQual rating: Low  Reason: Serious concerns regarding adequacy and coherence of findings. |
| *Target* | Description: An expected achievement level is set for clinical performance, generally according to expert opinion. This is different to *Benchmarking*, where others’ clinical performance is presented but without explicit judgment as to what levels of achievement are expected. | Supporting paper IDs (n=4): S109, S117, S154, 2794 |
|  | Effect hypothesis and mechanism (1): Facilitates *Goal setting, Perception* and *Intention* by decreasing *Complexity* (making it easier for recipients to know what constitutes ‘good performance’ and therefore what requires a corrective response)*.* | Supporting paper IDs (n=2): S117, S154  Theories: Feedback Intervention Theory ([disagrees with Normative information]; Goal setting); Control Theory (Comparator); Goal Setting Theory (Goal)  GRADE-CERQual rating: Low  Reason: Serious concerns regarding adequacy and relevance of findings. |
|  | Effect hypothesis and mechanism (2): Inhibits *Acceptance* by decreasing *Credibility* if targets are set too high*.* | Supporting paper IDs (n=2): S117, 2794  Theories: Feedback intervention theory (Eliminating Feedback-Standard Gap strategies)  GRADE-CERQual rating: Low  Reason: Serious concerns regarding adequacy and relevance of findings. |
| *Timeliness* | Description: How quickly the feedback message is sent relative to the time the clinical performance actually occurred. This could be near real-time (e.g. 1948) or years (e.g. 5033). | Supporting paper IDs (n=17): 1948, 2841, 2857, 3351, 5033, 5532, 5857, 7816, S1, S104, S105, S118, S120, S132, S154, S17, S25 |
|  | Effect hypothesis and mechanism (1): Increased timeliness facilitates *Interaction, Perception, Acceptance, Intention, Behaviour* and *Performance improvement* by increasing *Actionability* (because more timely data is easier to action), *Credibility* (because more timely data represents a more current picture of performance), and *Relative advantage* (because many feedback interventions do not provide timely data). | Supporting paper IDs (n=15): 1948, 2841, 2857, 5857, 3351, S105, S118, 5033, 5532, S1, S120, S104, S132, S17, S25  Theories: Individual Feedback Theory (Timing)  GRADE-CERQual rating: High |
| *Trend* | Description: The feedback message provides information on the recipients’ historical clinical performance, in addition to their most current. May correlate with *Graphical*, as often trend data is presented as line graphs. | Supporting paper IDs (n=18): 187, 5033, 512, 5357, 5532, 5857, 7049, 7050, S105, S118, S127, S132, S150, S17, S175, S4, S7, S71 |
|  | Effect hypothesis and mechanism (1): Facilitates *Perception,* and *Intention,* by decreasing *Complexity* (helping interpret and identify when clinical performance requires action). | Supporting paper IDs (n=11): S132, S150, S175, 187, 512, 5357, 5857, 7050, 5532, 7049, S105, S118, S17, S175, S7, S71  Theories: Cognitive Fit, Feedback Intervention Theory (Velocity)  GRADE-CERQual rating: High |
|  | Effect hypothesis and mechanism (2): Can increase *Observability* (ripple effect) by demonstrating how health professionals’ clinical performance has improved over time with the feedback intervention (if it has). | Supporting paper IDs (n=7): 5033, 5357, 5532, 5857, 7049, 7050, S7  GRADE-CERQual rating: Moderate  Reason: Minor concerns about adequacy and coherence of findings. |
| *Usability* | Description: The perceived user-friendliness and clarity of the feedback message. | Supporting paper IDs (n=24): 2794, 2841, 2857, 5235, 5532, 7194, S1, S104, S105, S112, S118, S120, S127, S132, S136, S15, S16, S19, S30, S38, S6, S62, S71, S81 |
|  | Effect hypothesis and mechanism (1): Facilitates *Perception, Intention,* and *Behaviour* by increasing *Relative advantage* (because existing clinical information systems may be less user friendly) and *Resource match* (by requiring less resource to interpret its findings), and decreasing *Complexity* (making the feedback message easier to understand and act upon). | Supporting paper IDs (n=23): 2857, 7194, S105, S118, S127, S136, S15, 2794, 2841, 5235, S1, S105, S112, S120, S136, S16, S19, S30, S38, S6, S62, S71, S132  Theories: Cognitive Load Theory; Fit between Individuals, Task and Technology framework (Usability); Model of Information Systems Success (Usability); Technology Acceptance Model (Usability)  GRADE-CERQual rating: High |
| *Detailed patient-level information* | Description: The feedback message includes more detailed information than that provided by lists regarding the patients included in the calculation of clinical performance. For example, latest blood test results (e.g. S32). Correlates with *Patient lists,* as they often co-exist. | Supporting paper IDs (n=8): 1948, 2023, 2857, 7694, S127, S16, S32, S62 |
|  | Effect hypothesis and mechanism (1): Facilitates *Verification* and *Acceptance* by increasing *Credibility* (demonstrating to recipients how their clinical performance was calculated). | Supporting paper IDs (n=3) S127, S32, 7694  Theories: Individual Feedback Theory (Supporting data)  GRADE-CERQual rating: Moderate  Reason: Moderate concerns regarding adequacy of findings. |
|  | Effect hypothesis and mechanism (2): Facilitates *Perception, Intention* and *Behaviour (patient-level)* by increasing *Actionability* and decreasing *Complexity* (enabling recipients to easily understand how their clinical performance may be suboptimal, and which patients to target where appropriate). | Supporting paper IDs (n=5): 1948, 2857, S127, S32, S62  Theories: Individual Feedback Theory (Supporting data)  GRADE-CERQual rating: Moderate  Reason: Some concerns regarding adequacy of findings. |
|  | Effect hypothesis and mechanism (3): Facilitates *Patient-level* behaviour by increasing *Actionability* for individual patients. | Supporting paper IDs (n=3): 1948, 2857, S62  GRADE-CERQual rating: Moderate  Reason: Moderate concerns regarding adequacy of findings. |
| *Qualitative data* | Description: The feedback message includes qualitative comments about clinical performance. For example from individual patients (e.g. 512). | Supporting paper IDs (n=5): 1948, 512, 7694, S7, 5033 |
|  | Effect hypothesis and mechanism (1): Facilitates *Intention* and *Behaviour* by increasing *Credibility* (demonstrating how their clinical performance may impact individual patients) and *Compatibility* with *professional role*. | Supporting paper IDs (n=3) 1948, 512, 5033  GRADE-CERQual rating: Low  Reason: Serious concerns regarding adequacy of findings. |
| ***Feedback delivery*** | | |
| *Active delivery* | Description: How much the feedback message is actively ‘pushed’ to recipients. In general, feedback messages sent where users have to obtain the feedback themselves (e.g. web-based or computer application) are less active than those sent to them (e.g. via mail or e-mail), which are in turn less active than those delivered face-to-face. May correlate with *Peer discussion, Action planning* and *Problem solving* when delivered face-to-face, as they are often delivered concurrently. | Supporting paper IDs (n=31): 1271, 1591, 1948, 2023, 3351, 5033, 5857, 6087, 7025, 7049, 7194, 7694, 7783, S104, S105, S118, S120, S127, S132, S150, S17, S19, S25, S28, S33, S4, S52, S6, S62, S7, S81 |
|  | Effect hypothesis and mechanism (1a – effect tension): Facilitates *Interaction* by reducing *Complexity* (making the feedback message simpler to receive) and *Compatibility* (by aligning with their workflow processes). | Supporting paper IDs (n=22): 1271, 1591, 1948, 3351, 5033, 5857, 6087, 7049, 7194, 7694, 7783, S104, S118, S120, S132, S150, S17, S19, S4, S52, S6, S81  Theories: [Disagrees with Feedback Intervention Theory (Verbal)]  GRADE-CERQual rating: High |
|  | Effect hypothesis and mechanism (1b – effect tension): Inhibits *Interaction* if solely requires formal face-to-face feedback sessions by decreasing *Resource match* (as they require significant time commitment from recipients). | Supporting paper IDs (n=7): 1271, 5857, 7694, 7783, S120, S52, S6  GRADE-CERQual rating: High |
|  | Effect hypothesis and mechanism (1c – effect tension): Increases *Cost* by decreasing *Resource match* (ripple effect). | Supporting paper IDs (n=7): 1271, 5857, 7694, 7783, S120, S52, S6  GRADE-CERQual rating: High |
| *Delivery to a group* | Description: The feedback message is delivered to groups of health professionals rather than just individual health professionals. They will usually work together in the same organisation or team. May correlate with *Peer discussion, Action planning, Active delivery* and *Problem solving* when delivered face-to-face, as they are often delivered concurrently. | Supporting paper IDs (n=21): 1271, 2023, 3351, 5033, 5357, 5532, 5857, 7025, 7694, 7783, 7816, 8249, S132, S15, S150, S33, S52, S6, S7, S71, S81 |
|  | Effect hypothesis and mechanism (4): Improves *Teamwork* (ripple effect) by increasing *Social influence* (engaging the wider team towards the feedback intervention’s goal [Social proof, Liking, Reference group]). | Supporting paper IDs (n=10): 3351, 5357, 5532, 5857, 7025, 7783, S15, S52, S7, S71  Theories: Persuasion theory (Social Proof, Liking), Normative Social Influence, Self-Determination Theory (Relatedness), Reference group  GRADE-CERQual rating: High |
|  | Effect hypothesis and mechanism (2): Improves *Extra-organisational networks* (ripple effect) if feedback message is delivered to those recipients from different organisations. | Supporting paper IDs (n=5): 5857, 7025, 7783, 7816, 8249  GRADE-CERQual rating: Moderate  Reason: Moderate concerns regarding adequacy of findings. |
|  | Effect hypothesis and mechanism (3): Improves *Intra-organisational networks* (ripple effect) by engaging staff members within an organisation. | Supporting paper IDs (n=6): 5357, 5532, 7025, 7783, S52, S7  GRADE-CERQual rating: Moderate  Reason: Moderate concerns regarding adequacy of findings. |
|  | Effect hypothesis and mechanism (1): Improves the *feedback intervention attitude* (ripple effect) by engaging the wider team in the feedback intervention process. | Supporting paper IDs (n=4): 7025, S15, S33, S7  GRADE-CERQual rating: Low  Reason: Serious concerns regarding coherence and adequacy of findings. |
|  | Effect hypothesis and mechanism (5): Causes negative *Emotions* (e.g. embarrassment) (ripple effect) if clinical performance is poor. | Supporting paper IDs (n=2): S7, 1271  GRADE-CERQual rating: Low  Reason: Serious concerns regarding coherence and adequacy of findings. |
|  | Effect hypothesis and mechanism (6): Increases goal *Controllability*  (ripple effect) by making recipients realise their role in the wider group. | Supporting paper IDs (n=2): 5532, 5857  GRADE-CERQual rating: Low  Reason: Serious concerns regarding coherence and adequacy of findings. |
| *Frequency* | Description: The frequency of feedback messages produced and delivered to recipients on the same *Goal*. | Supporting paper IDs (n=8): 5532, 7194, S1, S104, S120, S14, S16, S17 |
|  | Effect hypothesis and mechanism (1a – effect tension): Increased frequency inhibits *Perception* by increasing *Complexity* (too much feedback makes it more difficult to understand) and decreasing *Resource match* (feedback provided too often gives less time to act on it). | Supporting paper IDs (n=4): 5532, 7194, S1, S120  Theories: Individual Feedback Theory (Frequency)  GRADE-CERQual rating: Moderate  Reason: Moderate regarding adequacy of findings. |
|  | Effect hypothesis and mechanism (1b – effect tension): Decreased frequency inhibits *Interaction* and *Intention* by increasing *Complexity* (making the feedback message more difficult to receive). | Supporting paper IDs (n=3): S17, S16, S14  Theories: Individual Feedback Theory (Frequency)  GRADE-CERQual rating: Low  Reason: Serious concerns regarding adequacy of findings. |
| *Function* | Description: The recipients’ perception of whether the function of the feedback intervention is to punish them for providing suboptimal care (punitive), or to support them improve care quality (supportive). Correlates with *Reporting, Ownership* and *Source – Location*, as they often co-exist. | Supporting paper IDs (n=34): 1271, 1948, 2249, 2857, 5033, 5357, 5857, 6087, 627, 7194, 8249, S1, S104, S105, S109, S112, S117, S118, S127, S136, S15, S159, S17, S19, S25, S28, S30, S33, S38, S52, S6, S62, S67, S7, S81 |
|  | Effect hypothesis and mechanism (1): Supportive functionality facilitates *Acceptance* by increasing *Compatibility* with recipients’ professional role, and motivation (as health professionals generally want to improve care quality), whereas a punitive functionality decreases *Compatibility* with their sense of autonomy. | Supporting paper IDs (n=18): 2249, 2857, 5357, 5857, 6087, 7194, S1, S104, S112, S118, S136, S19, S25, S28, S30, S6, S62, S7, S81  Theories: Self-determination Theory (Autonomy); COM-B System (Motivation); Motivation-Opportunities-Abilities Model (Motivation); Individual Feedback Theory (Feedback function), Diffusion of Innovations (Meaning)  GRADE-CERQual rating: High |
|  | Effect hypothesis and mechanism (2): Punitive functionality increases negative *Emotions* (ripple effect) (e.g. fear and anxiety) by decreasing *Compatibility* with recipients’ professional role, autonomy, and motivation (as health professionals generally want to improve care quality). | Paper (n=17): 1271, 1948, S104, S117, S127, S30, S38, S6, S67, S7  GRADE-CERQual rating: High |
|  | Effect hypothesis and mechanism (3a – effect tension): Punitive functionality may facilitate *Intention, Behaviour,* and *Performance improvement* through *Social influence* (as health professionals want to maintain their status as a high performing clinician [Reference group]). | Supporting paper IDs (n=10): 1948, 2249, 5033, S1, S104, S118, S19, S30, S33, S81  GRADE-CERQual rating: Moderate  Reason: Moderate regarding the coherence of findings. |
|  | Effect hypothesis and mechanism (3b – effect tension): Punitive functionality may inhibit *Intention, Behaviour,* and *Performance improvement* by decreasing *Compatibility* with recipients’ motivation (as they already want to improve care quality) and autonomy. | Supporting paper IDs (n=10): 1948, 2249, 5033, S1, S104, S118, S19, S30, S33, S81  GRADE-CERQual rating: Moderate  Reason: Moderate regarding the coherence of findings. |
|  | Effect hypothesis and mechanism (4): Punitive functionality facilitates *Tunnel vision* (unintended consequence) through *Social influence* (as health professionals tend to conform to authority [Authority]) and via *Compatibility* (to correct their own expectations of themselves [self-affirmation, Cognitive dissonance]). | Supporting paper IDs (n=2): 1948, S117  GRADE-CERQual rating: Low  Reason: Serious concerns regarding adequacy of findings |
| *External reporting* | Description: The feedback message is perceived by the recipient to also be communicated to additional entities. This may include external organisations such as governments (e.g. 2249) and insurance companies (e.g. S109), members of the public (e.g. S17), or those internal to the organisation such as managers (e.g. 1948). Correlates with *Function, Ownership* and *Source location*, as they often co-exist. | Supporting paper IDs (n=14): 1948, 2249, 5033, S104, S109, S127, S159, S17, S19, S28, S38, S67, S7  . |
|  | Effect hypothesis and mechanism (1): Increases negative *Emotions* (ripple effect) (e.g. anxiety, frustration) by decreasing *Relative advantage* and *Compatibility* with their professional role and motivation (as health professionals feel this violates their autonomy and questions their motivation to improve care quality). | Supporting paper IDs (n=2): 1948, S104, S109, S127, S28, S38, S67, S7  GRADE-CERQual rating: High |
|  | Effect hypothesis and mechanism (2): Leads to *Gaming* (unintended consequence) because health professionals want to appear high performing to both themselves (*Compatibility* [self-affirmation, Cognitive dissonance]) and to others *Social influence* [Reference group]). | Supporting paper IDs (n=2) 1948, S67  GRADE-CERQual rating: Low  Reason: Serious concerns regarding adequacy of findings. |
| *Source knowledge and skill* | Description: The perceived level of appropriate knowledge and skill of the person / organisation delivering the feedback message. This may relate to clinical knowledge and skills (e.g. 1591), or those relating to technical aspects of quality improvement (e.g. 2249). Correlates with *Source – Location*, as they often co-exist. | Supporting paper IDs (n=22): 1271, 1591, 2249, 5357, 5857, 7194, 7783, S1, S104, S112, S136, S19, S25, S28, S30, S38, S62, S67, S71, S81 |
|  | Effect hypothesis and mechanism (1): Greater source knowledge and skill facilitates *Acceptance* and *Intention* by increasing *Credibility, Compatibility, Relative advantage* (as the opportunity to receive feedback from a credible source is valued by health professionals, and aligns with their sense of autonomy), and harnessing *Social influence* (because recipients are more likely to be persuaded by a credible source of feedback). | Supporting paper IDs (n=15): 1271, 1591, 2249, 5857, 7194, 7783, S104, S112, S136, S19, S25, S28, S30, S62, S81  Theory: Individual Feedback Theory (Credibility), Persuasion Theory (Authority), Obedience to authority  GRADE-CERQual rating: High |
|  | Effect hypothesis and mechanism (2): Less source knowledge and skill increases negative *Emotions* (ripple effect) (e.g. frustration) by decreasing *Relative advantage* and *Compatibility* with *professional role* and *motivation* (as health professionals feel this violates their sense of autonomy). | Supporting paper IDs (n=4): 1271, S104, S38, S67  GRADE-CERQual rating: Moderate  Reason: Some concerns regarding adequacy of findings |
| *Source location* | Description: Whether the source is perceived to be internal or external to the health professionals’ organisation. Correlates with *Source – knowledge and skill*, as they often co-exist. | Supporting paper IDs (n=9): 1271, 2249, 5857, 7194, S28, S30, S38, S6, S67 |
|  | Effect hypothesis and mechanism (1): External source inhibits *Acceptance* by reducing *Compatibility* with recipients’ sense of autonomy. | Supporting paper IDs (n=5): 2249, 5857, 7194, S28, S30  GRADE-CERQual rating: Moderate  Reason: Minor concerns regarding adequacy and coherence of findings. |
|  | Effect hypothesis and mechanism (2): External source increases negative *Emotions* (ripple effect) (e.g. frustration) by reducing *Compatibility* with *professional role* (as health professionals feel this violates their autonomy). | Supporting paper IDs (n=4): S38, S6, S67  GRADE-CERQual rating: Low  Reason: Serious concerns regarding adequacy of findings. |
| *Medium* | Description: The format in which the feedback is delivered e.g. verbally, paper, electronically. | Supporting paper IDs (n=6): 1591, 3351, S1, S127, S30, S4 |
|  | Effect hypothesis and mechanism: No clear effect (not enough data to draw a conclusion). | N/A |

**Feedback variables**

| **Name** | **Description, effect hypothesis, and mechanism** | **Evidence** |
| --- | --- | --- |
| ***Health professional characteristics*** | | |
| *Feedback attitude* | Description: Degree of positivity towards feedback intervention in general (e.g. potential effectiveness S6), and related quality improvement and technical concepts (e.g. information technology). | Supporting paper IDs (n=24): 1271, 2249, 3351, 5357, 5857, 7301, 8249, S1, S112, S136, S14, S15, S16, S17, S175, S176, S28, S30, S38, S52, S6, S67, S7, S81 |
|  | Effect hypothesis and mechanism (1): A positive attitude towards feedback intervention facilitates *Data collection and analysis* and *Feedback* (when *Conducted by recipients), Interaction, Acceptance, Intention,* and *Behaviour* by increasing *Compatibility* (with recipients’ beliefs)*, Relative advantage* (harnessing their enthusiasm for something they feel is beneficial), and *Credibility* (because they already believe in the value of feedback). | Supporting paper IDs (n=20): 1271, 2249, 5357, 5857, 7301, 8249, S1, S136, S14, S15, S16, S17, S118, S175, S176, S28, S30, S38, S52, S6, S7  Theories: Technology Acceptance Model (Usefulness); COM-B System (Motivation); Motivation-Opportunities-Abilities Model (Beliefs); Theory of Planned Behaviour (Attitude)  GRADE-CERQual rating: High |
| *Emotions* | Description: A reaction to receiving feedback. May be positive (e.g. happy, encouraged) or negative (e.g. sad, anxious, angry). | Supporting paper IDs (n=23): 1271, 187, 1948, 2794, 4222, 512, 5532, 6087, 7025, S1, S104, S109, S112, S117, S118, S127, S132, S15, S150, S154, S158, S16, S17, S19, S25, S28, S30, S32, S38, S4, S6, S67, S7, S81 |
|  | Effect hypothesis and mechanism (1): Positive emotions facilitate *Intention, Behaviour,* and *Performance improvement* by increasing *Actionability* (making the recipient feel they can positively influence situations). | Supporting paper IDs (n=2): S104, S4  GRADE-CERQual rating: Low  Reason: Serious concerns regarding adequacy of findings. |
| *Knowledge and skills in clinical topic* | Description: The recipients’ awareness and understanding of the theory and performance of tasks relevant to the clinical performance topic in the feedback intervention. May correlate with *Clinical education* via a ripple effect (low confidence). | Supporting paper IDs (n=24): 1591, 187, 2023, 2794, 4222, 5532, 627, 7049, 7050, 7194, S1, S105, S117, S118, S127, S132, S15, S158, S25, S30, S4, S52, S6, S62 |
|  | Effect hypothesis and mechanism (1): Increased clinical knowledge and skills facilitates *Perception, Acceptance, Intention,* and *Behaviour* by increasing *Actionability* (reminding/providing the recipient with clinical skills and knowledge to improve their performance), *Resource match* (increasing resource of health professionals with the requisite knowledge and skills) and *Credibility* (by enabling to believe the feedback more). | Supporting paper IDs (n=14): 187, 2023, 4222, 7194, 2794, 7050, S117, S118, S132, S15, S158, S25, S30, S62  Theories: COM-B System (Capability); Motivation-Opportunities-Abilities Model (Ability)  GRADE-CERQual rating: High |
| *Knowledge and skills in quality improvement* | Description: The recipients’ awareness and understanding of theory and performance of tasks regarding relating to quality improvement (including the feedback intervention, and feedback intervention in general). Correlates with *Training and support* via a ripple effect (high confidence). | Supporting paper IDs (n=21): 2841, 3351, 4222, 5033, 5235, 5357, 5857, 627, 7049, 7050, 7816, 8249, S1, S118, S120, S127, S132, S15, S16, S175, S52, S6, S62, S7, S71, S81 |
|  | Effect hypothesis and mechanism (1): Increased quality improvement knowledge and skills facilitates *Goal setting*, *Data collection and analysis* and *Feedback* (when *Conducted by recipients), Perception, Acceptance, Intention, Behaviour* (both *Patient-level* – by providing skills on how to interpret feedback – and *Organisation-level* – by providing skills to plan and act improvement plans)*,* and *Performance improvement* by increasing *Actionability* (providing the recipient with technical skills and knowledge to improve their performance), *Resource match* (increasing resource of health professionals with the requisite knowledge and skills), and decreasing *Complexity* (making the feedback intervention simpler to engage with). | Supporting paper IDs (n=20): 2841, 3351, 5033, 5235, 5357, 5857, 627, 7049, 7050, S1, S120, S127, S132, S15, S16, S175, S6, S62, S7, S71  Theories: COM-B System (Capability); Motivation-Opportunities-Abilities Model (Ability)  GRADE-CERQual rating: High |
|  | Effect hypothesis and mechanism (2): Increased quality improvement knowledge and skills facilitates *Organisation-level* behaviour (ripple effect) by increasing *Actionability* (providing the recipient with technical skills and knowledge to improve their performance) by providing skills on how to interpret feedback, and to plan and act on improvement plans. | Supporting paper IDs (n=4): 5357, S1, S15, S16  GRADE-CERQual rating: Moderate  Reason: Moderate concerns regarding the adequacy of findings. |
| *Role* | Description: The job of the feedback recipient influenced the effectiveness of feedback (e.g. whether they were a nurse, doctor, or non-clinician). | Supporting paper IDs (n=25): 187, 1948, 2249, 3351, 5033, 5357, 6087, 627, 7194, 7694, 8167, S1, S117, S15, S17, S175, S176, S25, S28, S30, S4, S52, S6, S62, S7 |
|  | Effect hypothesis and mechanism: No clear effect (not enough data to draw a conclusion). | N/A |
| ***Behavioural response*** | | |
| *Patient-level vs organisation-level* | Description: Behaviour may relate to individual clinicians caring for individual patients, or the organisations in which this care takes place. Patient-level behaviours can occur either during (e.g. 3351) or outside (e.g. 187) the point-of-care; and may be either retrospective/remedial (e.g. S104) or prospective/preventive (e.g. S1). Organisation-level behaviours aim to systematically changing the way care is delivered by a health care organisation, and may relate to starting, stopping, or modifying: services and protocols (e.g. 1948), or staff roles and training (e.g. 2857). Patient-level behaviours result from feedback acting as a reminder or new knowledge regarding how to behave in future, or specific instructions where *Patient lists* are provided. Organisation-level behaviours require more interpretation and resource to enact (time, skill, knowledge etc). | Supporting paper IDs (n=58): 1271, 1591, 187, 1948, 2023, 2794, 2841, 2857, 3351, 4222, 5033, 512, 5235, 5357, 5532, 5857, 627, 7025, 7049, 7050, 7194, 7301, 7694, 7783, 7816, 8167, 8249, S1, S104, S105, S112, S117, S118, S120, S127, S132, S136, S14, S15, S158, S159, S16, S17, S175, S19, S25, S28, S30, S32, S38, S4, S52, S6, S62, S67, S7, S71, S81 |
|  | Effect hypothesis and mechanism (1): Organisation-level behaviour facilitates *Performance improvement* because it can facilitate multiple patient-level behaviours by augmenting the clinical environment in which they take place, whereas their absence can lead to limited effects only (*Actionability*). | Supporting paper IDs (n=10): 1948, 2023, 2857, 512, 7816, S19, S4, S1, S32, S118  Theory: Multilevel approach to change  GRADE-CERQual rating: High |
|  | Effect hypothesis and mechanism (2): Organisation-level behaviour can lead to increased *Resource* (ripple effect) via *Resource match* e.g. by recruiting new staff (e.g. 8167), purchasing new equipment (e.g. 5033), or freeing up existing resource as a result of organisational improvements (e.g. S32), in an attempt to improve clinical performance. | Supporting paper IDs (n=8): 5033, 7025, 8167, 8249, S118, S19, S25, S32  GRADE-CERQual rating: High |
| *Direction* | Description: The direction of behaviour required to improve on suboptimal performance. This may be an increase, decrease, change or maintenance of behaviour. | Supporting paper IDs (n=3): 187, 8167, S30 |
|  | Effect hypothesis and mechanism: No clear effect (not enough data to draw a conclusion). | N/A |

**Context variables**

| **Name** | **Description, effect hypothesis, and mechanism** | **Evidence** |
| --- | --- | --- |
| ***Organisation or team characteristics*** | | |
| *Champion* | Description: Individuals within an organisation who dedicate themselves to supporting, marketing, and ‘driving through’ an feedback intervention. | Supporting paper IDs (n=26): 1591, 1948, 2023, 2841, 2857, 3351, 4222, 5033, 5357, 5532, 5857, 7025, 7049, 7301, 7816, S120, S132, S15, S154, S176, S52, S6, S62, S67, S71, S81 |
|  | Effect hypothesis and mechanism (1): Facilitates *Goal setting*, *Data collection and analysis* and *Feedback* (when *Conducted collected by recipients*)*, Interaction, Intention,* and *Behaviour* by increasing *Resource match* and *Social influence* (by influencing others to become involved) | GRADE-CERQual rating: High  Supporting paper IDs (n=23): 1591, 1948, 2023, 2857, 3351, 4222, 5033, 5357, 5532, 5857, 7025, 7049, 7301, S120, S132, S15, S154, S176, S52, S62, S67, S71, S81  Theories: Diffusion of innovations (Champions), Consolidated Framework for Implementation Research (Champions), Persuasion theory (Social proof, Liking), Normative Social Influence |
| *Competing priorities* | Description: Number and relative importance of other jobs or responsibilities relevant to feedback recipients in addition to the feedback intervention, which are sufficiently different from the feedback intervention. Correlates with *Resource*, because competing priorities reduce resources. | Supporting paper IDs (n=47): 1271, 1591, 187, 1948, 2841, 2857, 3351, 4222, 512, 5357, 5857, 6087, 627, 7025, 7049, 7050, 7194, 7301, 7694, 7816, S1, S104, S105, S109, S112, S117, S118, S120, S127, S132, S14, S15, S154, S159, S16, S17, S176, S25, S28, S32, S38, S4, S52, S62, S67, S7, S71 |
|  | Effect hypothesis and mechanism (1): Inhibits *Data collection and analysis* and *Feedback* (when *Conducted collected by recipients*)*, Interaction, Perception, Intention,* and *Behaviour* by decreasing *Resource match* – especially if there is reduced *Compatibility* between the competing priorities and feedback intervention*.* | Supporting paper IDs (n=45): 1271, 1591, 187, 2841, 2857, 3351, 4222, 5357, 5857, 6087, 627, 7025, 7049, 7050, 7194, 7301, 7694, 7816, S1, S104, S105, S109, S112, S117, S118, S120, S127, S132, S14, S15, S154, S159, S16, S17, S176, S25, S28, S32, S38, S4, S52, S62, S67, S7, S71  Theories: Consolidated Framework for Implementation Research (Relative priority)  GRADE-CERQual rating: High |
|  | Effect hypothesis and mechanism (2): Leads to *Tunnel vision* (unintended consequence) by decreasing *Resource match* (less resource is available to focus on other aspects of care other than those measured by the feedback intervention)*.* | Supporting paper IDs (n=4): 1948, 512, 6087, S117  GRADE-CERQual rating: Low  Reasons: Serious concerns regarding the coherence and adequacy of the findings. |
|  | Effect hypothesis and mechanism (3): Increases negative *Emotions* (ripple effect) by decreasing *Resource match* (making working life more stressful) and *Compatibility* (reducing the ability of the recipient to focus on aspects of clinical work they feel may be more important). | Supporting paper IDs (n=3): 1271, 512, S1  GRADE-CERQual rating: Low  Reasons: Serious concerns regarding the adequacy of the findings. |
| *Similar quality improvement interventions* | Description: Presence of other quality improvement initiatives in addition to the feedback intervention. They may focus on similar clinical topics. Correlates with *Competing priorities,* as similar quality improvement initiatives may be viewed as such. | Supporting paper IDs (n=19): 1591, 187, 2841, 5357, 7049, 7050, 7816, 8167, S1, S112, S118, S127, S136, S15, S17, S175, S38, S6, S62, S67 |
|  | Effect hypothesis and mechanism (1a – effect tension): Increases *Workflow fit* (ripple effect) by increasing *Compatibility* (with existing workflows and priorities), *Resource match* (as resources to engage in feedback intervention may already exist from the other initiatives), and *Relative advantage* (if the feedback intervention has distinct advantages over the other interventions)*.* | Supporting paper IDs (n=18): 187, 2841, 5357, 7049, 7050, 7816, 8167, S1, S112, S118, S127, S136, S15, S17, S175, S38, S62, S67  GRADE-CERQual rating: Moderate  Reason: Moderate concerns regarding coherence of data. |
|  | Effect hypothesis and mechanism (1b – effect tension): Increases *Competing priorities* (ripple effect) by decreasing *Resource match* (if resources from engaging in the other initiatives are already used) and *Relative advantage* (if the feedback intervention does not have obvious advantages over the other interventions)*,* and increasing *Complexity* (by making it unclear which intervention should be given priority). | Supporting paper IDs (n=18): 187, 2841, 5357, 7049, 7050, 7816, 8167, S1, S112, S118, S127, S136, S15, S17, S175, S38, S62, S67  GRADE-CERQual rating: Moderate  Reason: Moderate concerns regarding coherence of data. |
| *Extra-organisational networks* | Description: The effectiveness of feedback intervention recipients’ organisations’ communications with other organisations. Correlates with *Peer discussion* through a ripple effect. | Supporting paper IDs (n=10): 1591, 7025, 7049, 7050, 7816, S105, S132, S15, S38, S62 |
|  | Effect hypothesis and mechanism (1): Facilitates *Perception, Intention,* and *Behaviour* by increasing *Actionability* and *Resource match* (providing practical support on interpreting and responding to issues in a feedback message). | Supporting paper IDs (n=9): 1591, 7025, 7049, 7050, S105, S132, S15, S38, S62  Theories: Diffusion of Innovations (Extra-organisational networks); Consolidated Framework for Implementation Research (Cosmopolitanism); Social Learning Theory (Copying / Matched-dependent behaviour)  GRADE-CERQual rating: High |
| *Intra-organisational networks* | Description: The effectiveness of communications with the feedback intervention recipients’ organisations. Correlates with *Teamwork,* as good intra-organisational networks are a feature of good teamwork. Correlates with *Peer discussion* and *Delivery to a group* through ripple effects (moderate confidence). | Supporting paper IDs (n=22): 2023, 2857, 4222, 5033, 5357, 5532, 627, 7049, 7050, 7816, 8167, S105, S109, S117, S132, S15, S175, S32, S38, S6, S71, S81 |
|  | Effect hypothesis and mechanism (1): Facilitates *Data collection and analysis* and *Feedback* (when *Conducted by recipients), Interaction, Intention,* and *Behaviour* by increasing *Actionability* and *Resource match* (providing practical support between colleagues on producing, communicating and responding effectively to feedback messages), *Compatibility* (with existing communication channels), and *Social influence* (by engaging the wider team towards the feedback intervention’s goal [Social proof, Liking, Reference group]). | Supporting paper IDs (n=19): 2857, 4222, 5033, 5532, 627, 7049, 7050, 8167, S105, S109, S117, S132, S15, S175, S32, S38, S6, S71, S81  Theories: Persuasion theory (Social Proof, Liking), Normative Social Influence, Self-Determination Theory (Relatedness), Reference group, Diffusion of Innovations (Intra-organisational networks), Consolidated Framework for Implementation Research (Networks & Communications)  GRADE-CERQual rating: High |
| *Leadership support* | Description: Advocacy for the feedback intervention from members of top management within the feedback intervention recipients’ organisation. | Supporting paper IDs (n=23): 1271, 1948, 2023, 4222, 512, 5357, 5532, 7025, 7050, 7816, 8167, S105, S117, S118, S132, S154, S16, S176, S19, S52, S62, S71, S81 |
|  | Effect hypothesis and mechanism (1a): Facilitates *Data collection and analysis* and *Feedback* (when *Conducted by recipients), Interaction, Intention,* and *Behaviour* by increasing *Social influence* (encouraging, permitting, and instructing staff to engage and respond [Influence theory – Authority]), *Resource match* (providing additional resources to engage with the feedback intervention as necessary), and *Credibility* (setting an example to health professionals to engage with the feedback intervention). | Supporting paper IDs (n=23): 1271, 1948, 2023, 4222, 512, 5357, 5532, 7025, 7050, 7816, 8167, S105, S117, S118, S132, S154, S16, S176, S19, S52, S62, S71, S81  Theories: Consolidated Framework for Implementation Research (Leadership Engagement); Diffusion of Innovations (Staff involvement and commitment); persuasion theory (Authority); Obedience to authority  GRADE-CERQual rating: High |
|  | Effect hypothesis and mechanism (1b): Facilitates *Organisation-level* behaviour (ripple effect) by increasing *Social influence* (encouraging, permitting, and instructing staff to engage and respond [Influence theory – Authority]), *Resource match* (providing additional resources to engage with the feedback intervention as necessary), and *Credibility* (setting an example to health professionals to engage with the feedback intervention). | Supporting paper IDs (n=6): 1948, 512, 8167, S118, S81, S16  Theories: Consolidated Framework for Implementation Research, Diffusion of innovations, persuasion theory (Authority)  GRADE-CERQual rating: High |
| *Opinion leaders* | Description: Advocacy for the feedback intervention from members of staff within the feedback intervention recipients’ organisation who exert either formal or informal influence on the attitudes and beliefs of their colleagues through their authority, status, and credibility. Correlates with *Champions* and *Leadership support* because often opinion leaders are often all three. | Supporting paper IDs (n=10): 1591, 2023, 5357, 5857, 7816, 8167, S132, S15, S176, S62 |
|  | Effect hypothesis and mechanism (1): Facilitates *Interaction, Intention, Behaviour,* and *Performance improvement* by increasing *Social influence* (encouraging health professionals to engage with the feedback intervention [Influence theory – Authority and Liking]), and *Credibility* (setting an example to health professionals to engage with the feedback intervention). | Supporting paper IDs (n=7): 2023, 5357, 5857, 8167, S132, S15, S176, S62.  Theories: Persuasion theory (Authority, Liking); Diffusion of Innovations (Opinion leaders); Consolidated Framework for Implementation Research (Opinion leaders)  GRADE-CERQual rating: Moderate  Reason: Moderate concerns regarding the adequacy and coherence of findings. |
| *Resource* | Description: The amount of material and non-material resource available within the feedback intervention recipients’ organisation, including financial resource, human resource, time, space, and equipment. Correlates with *Competing priorities*, because competing priorities reduce resources. | Supporting paper IDs (n=49): 1271, 1591, 187, 1948, 2023, 2794, 2841, 2857, 3351, 4222, 5033, 512, 5235, 5357, 5532, 5857, 6087, 627, 7025, 7049, 7194, 7301, 7694, 7783, 7816, 8167, S1, S104, S105, S109, S112, S117, S118, S120, S127, S132, S14, S15, S16, S17, S19, S25, S30, S32, S38, S4, S52, S6, S62, S67, S71, S81 |
|  | Effect hypothesis and mechanism (1a): Facilitates *Data collection and analysis* and *Feedback* (when *Conducted by recipients), Interaction, Perception, Intention,* and *Behaviour* by increasing *Resource match*. | Supporting paper IDs (n=48): 1271, 187, 2023, 2794, 2841, 2857, 3351, 4222, 5033, 512, 5235, 5357, 5532, 5857, 6087, 627, 7025, 7049, 7194, 7301, 7694, 7816, 8167, S1, S104, S105, S109, S112, S117, S118, S120, S127, S132, S14, S15, S16, S17, S19, S25, S30, S32, S38, S4, S52, S6, S62, S67, S81  Theories: Diffusion of Innovations (Dedicated time and resources); Consolidated Framework for Implementation Research (Available resources)  GRADE-CERQual rating: High |
|  | Effect hypothesis and mechanism (1b): Facilitates *Organisation-level* behaviour (ripple effect) by increasing *Resource match*. | Supporting paper IDs (n=7): 2023, 2857, 6087, 8167, S118, S19, S4  GRADE-CERQual rating: High |
|  | Effect hypothesis and mechanism (2): Leads to *Tunnel vision* (unintended consequence) by decreasing *Resource match* (less resource is available to focus on other aspects of care other than those measured by the feedback intervention)*.* | Supporting paper IDs (n=3): 1948, 512, 6087  GRADE-CERQual rating: Low  Reasons: Serious concerns regarding the coherence and adequacy of the findings. |
| *Staff turnover* | Description: The rate of staff at the feedback intervention recipients’ organisation replaced by new employees. | Supporting paper IDs (n=7): 2794, 7049, 7050, S118, S120, S127, S14 |
|  | Effect hypothesis and mechanism (1): Inhibits *Behaviour,* and *Clinical performance improvement* by decreasing *Resource match* (new staff require additional resource to train them in and make them aware of the feedback intervention). | Supporting paper IDs (n=7): 2794, 7049, 7050, S118, S120, S127, S14  GRADE-CERQual rating: Moderate  Reason: Moderate concerns regarding the adequacy of findings. |
| *Teamwork* | Description: The ability of the feedback intervention recipients’ organisation to work together effectively or cohesively towards a common goal. Correlates with *Intra-organisational networks,* as good intra-organisational networks are often a feature of good teamwork. | Supporting paper IDs (n=25): 2023, 2857, 3351, 4222, 5357, 5532, 6087, 627, 7049, 7050, 7301, 7783, 8167, S105, S117, S118, S132, S15, S175, S32, S38, S6, S67, S71, S81 |
|  | Effect hypothesis and mechanism (1a): Facilitates *Data collection and analysis* and *Feedback* (when *Conducted by recipients), Perception, Intention,* and *Behaviour* by increasing *Actionability* and *Resource match* (by providing practical support to colleagues on producing, communicating and responding effectively to feedback messages), and *Social influence* (by engaging the wider team towards the feedback intervention’s goal [Social proof, Liking, Reference group]). | Supporting paper IDs (n=22): 2023, 2857, 3351, 4222, 5532, 627, 7049, 7050, 7301, 7783, 8167, S105, S117, S118, S132, S15, S175, S32, S38, S6, S67, S71  Theories: Persuasion theory (Social Proof, Liking), Normative Social Influence, Self-Determination Theory (Relatedness), Reference group  GRADE-CERQual rating: High |
|  | Effect hypothesis and mechanism (1b): Facilitates *Organisation-level* behaviour (ripple effect) by increasing *Actionability* (providing practical support between colleagues on responding effectively to feedback messages). | Supporting paper IDs (n=3): 2857, 8167, S118  GRADE-CERQual rating: Moderate  Reason: Concerns regarding the adequacy of findings. |
| *Workflow fit* | Description: The degree of alignment of the feedback intervention with the systems used and processes conducted by the recipient or their wider organisation. Understandably, this depends on the specific context into which the intervention is being implemented. | Supporting paper IDs (n=18): 187, 2841, 2857, 3351, 7194, 7301, 7816, S1, S105, S112, S132, S14, S15, S176, S32, S6, S62, S67, S71 |
|  | Effect hypothesis and mechanism (1): Facilitates *Data collection and analysis* and *Feedback* (when *Conducted by recipients), Interaction, Perception, Acceptance, Intention, Behaviour,* and *Performance improvement* by increasing *Compatibility* (with existing workflows and systems), *Actionability* (ensuring they can engage with the feedback intervention during their working lives) and reducing *Complexity* (by reducing the need to change their workflows to integrate the feedback intervention). | Supporting paper IDs (n=18): 187, 2841, 2857, 3351, 7194, 7816, S1, S105, S112, S132, S14, S15, S176, S32, S6, S62, S67, 71  Theories: Diffusion of innovations in health service delivery and organisation (Compatibility); Fit between Individuals, Task and Technology framework (Fit); Task-Technology-Fit Model (Fit).  GRADE-CERQual rating: High |
| ***Patient population*** | | |
| *Choice alignment* | Description: Reasons for patients receiving suboptimal (measured) clinical care relating to their decisions or expectations.  Relevant papers (n=16) | Supporting paper IDs (n=16): 187, 512, 6087, 7049, 7050, 7194, 7301, S1, S104, S112, S117, S127, S28, S30, S32, S38 |
|  | Effect hypothesis and mechanism (1): Inhibits *Acceptance, Intention,* and *Behaviour,* and *Clinical performance improvement* by decreasing *Actionability* (either the health professionals have little control over the care provided – leading to non-acceptance, or they cannot act upon it to improve), *Compatibility* (with health professionals’ goals to provide patient-centred care), and *Complexity*. | Supporting paper IDs (n=13): 187, 6087, 7049, 7050, 7194, 7301, S104, S127, S28, S30, S32, S38, S6  Theories: Cabana Guideline model, Guidelines interdependence model  GRADE-CERQual rating: High |
|  | Effect hypothesis and mechanism (2): Leads to *Gaming* and *Inappropriate care* (unintended consequence) especially in the presence of *Financial reward* in an attempt to increase *Resource match* (to maintain resource, attempts to preserve financial income are preserved at any cost). | Supporting paper IDs (n=3): 6087, S112, S38  GRADE-CERQual rating: Low  Reason: Serious concerns regarding adequacy of findings. |
|  | Effect hypothesis and mechanism (3): Increases negative *Emotions* (ripple effect) by decreasing *Compatibility* (between recipients’ *motivations* and *expectations,* because health professionals generally strive to provide high quality patient-centred care). | Supporting paper IDs (n=3): S1, S112, S30GRADE-CERQual rating: Low  Reason: Serious concerns regarding adequacy of findings. |
| *Clinical appropriateness* | Description: Explanations for patients receiving suboptimal (measured) clinical care relating to the their specific clinical characteristics. This may include where guideline-recommended care is contraindicated due to medication allergies or existing conditions (e.g. 187), or their response to treatment is diminished because they are already on maximally tolerated therapy (e.g. S117). | Supporting paper IDs (n=16): 1591, 187, 2794, 4222, 6087, 8167, S1, S104, S112, S117, S127, S17, S30, S32, S67, S7 |
|  | Effect hypothesis and mechanism (1): Inhibits *Acceptance, Intention, Behaviour,* and *Clinical performance improvement* by decreasing *Actionability* (either the health professionals have little control over the care provided – leading to non-acceptance, or they cannot act upon it to improve), *Compatibility* (with health professionals’ goals to provide patient-centred care), and *Complexity*. | Supporting paper IDs (n=12): 1591, 187, 2794, 4222, 6087, S1, S104, S17, S117, S30, S32, S6  Theories: Cabana Guideline model, Guidelines interdependence model  GRADE-CERQual rating: High |
|  | Effect hypothesis and mechanism (3): Leads to *Gaming* (unintended consequence) in attempt to increase *Compatibility* (between recipients’ *motivations* and *expectations,* because health professionals generally strive to provide high quality patient-centred care). | Supporting paper IDs (n=4): 8167, S112, S127, S67  Theories: Cognitive dissonance  GRADE-CERQual rating: Moderate  Reason: Moderate regarding the adequacy of findings. |
|  | Effect hypothesis and mechanism (2): Leads to *Verification* by decreasing *Compatibility* (between recipients’ *motivations* and *expectations,* because health professionals generally strive to provide high quality patient-centred care) and *Credibility* (recipients will want to interrogate clinical performance data to look for reasons for suboptimal care). | Supporting paper IDs (n=2): 1591, S104  GRADE-CERQual rating: Low  Reason: Serious concerns regarding the adequacy of findings. |
|  | Effect hypothesis and mechanism (4): Increases negative *Emotions* (ripple effect) by decreasing *Compatibility* (between recipients’ *motivations* and *expectations,* because health professionals generally strive to provide high quality patient-centred care). | Supporting paper IDs (n=3): S1, S117, S30  GRADE-CERQual rating: Low  Reason: Serious concerns regarding adequacy of findings. |
| ***Co-interventions*** | | |
| *Problem solving* | Description: Analysis of reasons for sub-optimal clinical performance and formulation of solution(s) to address them. This may be performed as part of the feedback intervention, or the recipients may be required/supported to do it themselves – either on their own (e.g. S1); or with support from an *External change agent* (e.g. S158), the feedback provider (e.g. S15), or peers (e.g. S109). May correlate with *Action planning, Peer discussion, Active delivery* (when delivered face-to-face) and *Delivery to a group* (when delivered face-to-face) as they are often delivered concurrently. | Supporting paper IDs (n=22): 1271, 1948, 4222, 5857, 627, 7025, 7301, 7783, S1, S109, S117, S118, S127, S132, S15, S150, S158, S175, S25, S62, S71, S81 |
|  | Effect hypothesis and mechanism (1): Facilitates *Perception*, *Intention,* *Behaviour* and *Clinical performance improvement* by increasing *Actionability* (by providing practical support on how to act effectively to the feedback message) and *Resource match* (by addressing health professionals’ general lack of knowledge and skills to perform these behaviours). | Supporting paper IDs (n=22): 1271, 5857, 7025, 7301, S1, S109, S118, S127, S132, S15, S150, S25, 1948, 4222, 7783, S117, S118, S158, S175, S62, S71, S81  Theories: Feedback Intervention Theory (Correct solution)  GRADE-CERQual rating: High |
|  | Effect hypothesis and mechanism (2): Facilitates *Organisation-level* behaviour (ripple effect) by increasing *Actionability* (by providing practical support on how to act effectively to the feedback message), *Compatibility* (by aiming to provide tailored improvement solutions to health professionals) and *Resource match* (by addressing health professionals’ general lack of knowledge and skills to perform these behaviours). | Supporting paper IDs: 1948, 7783, S175  GRADE-CERQual rating: Moderate  Reasons: Minor concerns regarding adequacy and coherence of data. |
|  | Effect hypothesis and mechanism (3): Increases Teamwork (ripple effect) if *Problem solving* occurs through discussions between staff in the same organisation. | Supporting paper IDs (n=4): 5857, 7025, S109, S71  GRADE-CERQual rating: Low  Reason: Substantial concerns regarding adequacy and coherence of data. |
|  | Effect hypothesis and mechanism (4): Increases positive Emotions (ripple effect) if done in a supportive environment. | Supporting paper IDs (n=3): S127, S150, S158  GRADE-CERQual rating: Low  Reason: Substantial concerns regarding adequacy and coherence of data. |
|  | Effect hypothesis and mechanism (5): Increases Knowledge and skills in clinical topic (ripple effect). | Supporting paper IDs (n=3): 4222, S118, S158.  GRADE-CERQual rating: Low  Reason: Substantial concerns regarding adequacy and coherence of data. |
| *Action planning* | Description: Instructions for specific behaviours to improve clinical performance. They may be provided as part of the feedback intervention (e.g. 1271, S112) or generated by the recipients themselves (e.g. 7783, S1). Differs from *Problem solving* because suggested actions are not necessarily based on analysis of reasons for sub-optimal clinical performance; though may be correlated as they can co-exist. May also correlate with *Active delivery* (when delivered face-to-face)*, Delivery to a group* (when delivered face-to-face) and *Peer discussion* as they are often delivered concurrently. | Supporting paper IDs (n=24): 1271, 187, 1948, 5033, 5235, 5857, 7025, 7194, 7783, 8167, S1, S112, S117, S118, S120, S127, S15, S158, S16, S175, S30, S62, S71, S81 |
|  | Effect hypothesis and mechanism (1): Facilitates *Intention*, *Behaviour* and *Clinical performance improvement* by increasing *Actionability* (providing practical support on how to respond effectively to the feedback message) and *Resource match* (by addressing health professionals’ general lack of knowledge and skills to perform these behaviours). | Supporting paper IDs (n= 21): 1271, 187, 1948, 5033, 5235, 5857, 7025, 7783, 8167, S1, S112, S117, S120, S127, S15, S158, S175, S6, S62, S71, S81  Theories: Feedback Intervention Theory (Correct solution)  GRADE-CERQual rating: High |
|  | Effect hypothesis and mechanism (2): Facilitates *Organisation-level* behaviour (ripple effect) by increasing *Actionability* (by providing practical support on how to act effectively on the feedback message) and *Resource match* (by addressing health professionals’ general lack of knowledge and skills to perform these behaviours). | Supporting paper IDs (n=3): 1948, 7783, S175  GRADE-CERQual rating: Moderate  Reasons: Concerns regarding adequacy of data. |
| *Reminders* | Description: Point-of-care alerts (usually) integrated into a patient’s electronic health record (EHR) to prompt clinicians to recognise which clinical performance measures the patient has not achieved. These are generally only encountered when the EHR is opened by a clinician during a consultation with the patient. | Supporting paper IDs (n=4): S112, S117, S4, S62 |
|  | Effect hypothesis and mechanism: No clear effect (not enough data to draw a conclusion). | N/A |
| *External change agent* | Description: The presence of individuals not affiliated with the recipients’ own organisation whose intention is to facilitate feedback intervention processes. This can could be direct, for example by performing improvement behaviours themselves (e.g. 1591); or indirect by helping with action planning or problem solving (e.g. S62). | Supporting paper IDs (n=17): 1591, 2794, 2841, 4222, 5857, 627, 7025, 7049, 7050, 7783, S1, S120, S15, S158, S25, S62, S81 |
|  | Effect hypothesis and mechanism (1): Dependent on their specific role, External change agents can positively influence all feedback intervention processes by providing an additional resource to increase *Resource match*. | Supporting paper IDs (n=17): 1591, 2794, 2841, 4222, 5857, 627, 7025, 7049, 7050, 7783, S1, S120, S15, S158, S25, S62, S81  Theories: Diffusion of innovations (Role of change agency); Consolidated Framework for Implementation Research (External Change Agents)  GRADE-CERQual rating: High |
|  | Effect hypothesis and mechanism (2): Increases *Knowledge and skills – Quality improvement* (ripple effect) via *Social influence* (imparting knowledge to recipients) and *Credibility*. | Supporting paper IDs (n=3): S81, S62, S120  Theories: Social Learning Theory (Copying)  GRADE-CERQual rating: Moderate  Reason: Moderate concerns regarding the adequacy of findings. |
| *Financial reward* | Description: Payment received for either participating in, or improving clinical performance during, a feedback intervention. | Supporting paper IDs (n=17): 187, 2857, 5033, 6087, 627, S104, S109, S112, S117, S127, S136, S15, S30, S38, S4, S6, S7 |
|  | Effect hypothesis and mechanism (1a – effect tension): Inhibits *Acceptance* by reducing *Compatibility* with recipients’ professional role. | Supporting paper IDs (n=11): 187, 2857, 5033, S104, S109, S112, S117, S136, S15, S30, S7  Theories: Self-determination Theory (Intrinsic motivation); COM-B System (Motivation); Motivation-Opportunities-Abilities Model (Beliefs) [Disagrees with theories in effect hypothesis and mechanism 1b]  GRADE-CERQual rating: Moderate  Reason: Minor concerns regarding adequacy, and moderate concerns regarding coherence of findings. |
|  | Effect hypothesis and mechanism (1b – effect tension): Facilitates Interaction, Intention and Behaviour, possibly by increasing *Resource match* and *Relative advantag*e. | Supporting paper IDs (n=11): 187, 2857, 5033, S104, S109, S112, S117, S136, S15, S30, S7  Theories: Individual Feedback Theory (Rewards), Consolidated Framework for Implementation Research (Organizational Incentives & Rewards) [Disagrees with theories in effect hypothesis and mechanism 1a]  GRADE-CERQual rating: Moderate  Reason: Minor concerns regarding adequacy, and moderate concerns regarding coherence of findings. |
|  | Effect hypothesis and mechanism (2): Leads to *Gaming* (unintended consequence) by increasing *Resource match* (in an attempt to maintain resource, attempts to preserve financial income are preserved at any cost). | Supporting paper IDs (n=2): S109, S112  GRADE-CERQual rating: Low  Reason: Serious concerns regarding adequacy of findings. |
|  | Effect hypothesis and mechanism (4): Increases *Resource* (ripple effect). | Supporting paper IDs (n=2): 627, S109  GRADE-CERQual rating: Low  Reason: Serious concerns regarding adequacy of findings. |
| *Non-financial reward* | Description: A reward other than payment for either participating in, or improving clinical performance during an feedback intervention. For example, receiving a certificate or praise from a superior. | Supporting paper IDs (n=5): 1271, S104, S15, S30, S4 |
|  | Effect hypothesis and mechanism: No clear effect (not enough data to draw a conclusion). | N/A |
| *Peer discussion* | Description: feedback intervention recipients receive help from peers in discussing and reflecting on their clinical performance. This may be informal (e.g. 4222) or formal discussions (e.g. 7025), between colleagues in the same (e.g. 4222) or different (e.g. 7025) organisations. May be correlated with *Action planning*, *Problem solving*, *Active delivery* (when delivered face-to-face) and *Delivery to a group* (when delivered face-to-face) as they are often delivered concurrently. | Supporting paper IDs (n=29): 1271, 1948, 2023, 4222, 5357, 5857, 627, 7025, 7049, 7194, 7301, 7694, 7783, S1, S104, S105, S120, S127, S132, S15, S154, S158, S175, S19, S30, S33, S52, S62, S71 |
|  | Effect hypothesis and mechanism (1a): Facilitates *Perception, Intention*, *Behaviour*, and *Clinical performance improvement* by increasing *Actionability* (by providing practical support on how to act effectively to the feedback message), *Resource match* (by addressing health professionals’ general lack of knowledge and skills to perform these behaviours), and *Social influence* (by demonstrating how peers would address the feedback and increasing engagement). | Supporting paper IDs (n=26): 1948, 2023, 4222, 5357, 5857, 7025, 7049, 7194, 7301, 7694, 7783, S104, S105, S120, S127, S132, S15, S154, S158, S175, S19, S30, S33, S52, S62, S71  Theories: Self-Determination Theory (Relatedness); Social Learning Theory (Copying / Matched-dependent behaviour); Diffusion of Innovations (Intentional spread strategies)  GRADE-CERQual rating: High |
|  | Effect hypothesis and mechanism (1b): Facilitates *Organisation-level* behaviour (ripple effect) by increasing *Actionability* (by providing practical support on how to act effectively to the feedback message) and *Resource match* (by addressing health professionals’ general lack of knowledge and skills to perform these behaviours). | Supporting paper IDs (n=4): 1948, 2023, 7783, S175  GRADE-CERQual rating: Moderate  Reasons: Moderate concerns regarding adequacy of data |
|  | Effect hypothesis and mechanism (2): Increases *Teamwork* (ripple effect) by increasing *Social influence* (engaging the wider team towards the feedback intervention’s goal [Social proof, Liking, Reference group]). | Supporting paper IDs (n=7): 5357, 5857, 7025, 7783, S105, S15, S71  Theories: Self-Determination Theory (Relatedness), Persuasion theory (Social proof, Liking), Reference group, Normative Social Influence  GRADE-CERQual rating: High |
|  | Effect hypothesis and mechanism (3): Facilitates Acceptance and Perception by reducing Complexity (making the feedback easier to understand). | Supporting paper IDs (n=12): 1271, 2023, 4222, 5857, 7694, 7783, S127, S15, S158, S19, S33, S71  GRADE-CERQual rating: Moderate  Reasons: Minor concerns regarding methodological limitations and adequacy of findings. |
|  | Effect hypothesis and mechanism (4): Increases *Delivery – Active* (ripple effect) by ensuring that recipients receive the feedback message face-to-face. | Supporting paper IDs (n=9): 1948, 2023, 7025, 7049, 7783, S120, S19, S30, S71  GRADE-CERQual rating: Moderate  Reasons: Minor concerns regarding methodological limitations and adequacy of findings. |
|  | Effect hypothesis and mechanism (5): Improves the *feedback intervention attitude* (ripple effect) by increasing *Social influence* (engaging the wider team in the feedback intervention process [Social proof, Liking]). | Supporting paper IDs (n=4): 4222, 7025, S15, S33  GRADE-CERQual rating: Moderate  Reasons: Minor concerns regarding methodological limitations and adequacy of findings. |
|  | Effect hypothesis and mechanism (6): Improves the *Extra-organisational networks* (ripple effect) if Peer discussion provided by recipients from different organisations. | Paper (n=5): 5857, 7025, 7783, 7816, S105  GRADE-CERQual rating: Moderate  Reason: Moderate concerns regarding adequacy of findings. |
|  | Effect hypothesis and mechanism (7): Improves the *Intra-organisational networks* (ripple effect) by engaging staff members within an organisation. | Paper (n=5): 5357, 5857, 7025, 7783, S105  GRADE-CERQual rating: Moderate  Reason: Moderate concerns regarding adequacy of findings. |
|  | Effect hypothesis and mechanism (8): Increases *Knowledge and skills in clinical topic* (ripple effect). | Supporting paper IDs (n=3): 4222, S105, S158.  GRADE-CERQual rating: Low  Reason: Serious concerns regarding adequacy of findings. |
| *Clinical education* | Description: feedback intervention recipients receive advice or instruction on how to perform clinical behaviours, or regarding the clinical outcomes, relevant to the clinical performance under measurement. | Supporting paper IDs (n=8): 2023, 2794, 2857, 4222, 5357, 5857, S30, S62 |
|  | Effect hypothesis and mechanism (1): Increases *Knowledge and skills in clinical topic* (ripple effect). | Supporting paper IDs (n=4): 2023, 2794, 2857, S62  GRADE-CERQual rating: Low  Reasons: Serious concerns regarding adequacy and methodological limitations of findings. |
|  | Effect hypothesis and mechanism (2): Increases Teamwork, especially if educational sessions delivered to a multidisciplinary group. | Supporting paper IDs (n=2): 5357, 5857  GRADE-CERQual rating: Low  Reasons: Serious concerns regarding methodological limitations, coherence and adequacy of findings. |
| ***Implementation process*** | | |
| *Adaptability* | Description: Whether or not the feedback intervention can be tailored to the context into which it is implemented to meet their perceived needs. This may relate to any aspect of the feedback intervention process e.g. design of the feedback message or how the data is collected during Data collection and analysis. | Supporting paper IDs (n=16): 1271, 2841, 3351, 4222, 5357, 7049, 7050, 7194, S117, S118, S127, S132, S15, S62, S67, S81 |
|  | Effect hypothesis and mechanism (1): Facilitates *Goal setting, Data collection and analysis, Interaction, Acceptance, Intention, Behaviour,* and *Performance improvement* by increasing *Compatibility* (with their preferences) and decreased *Complexity* (to implement)*.* | Supporting paper IDs (n=14): 1271, 2841, 3351, 4222, 5357, 7049, 7050, 7194, S117, S118, S127, S132, S62, S67, S81  GRADE-CERQual rating: High  Theories: Diffusion of innovations (Re-invention), Consolidated Framework for Implementation Research (Adaptability) |
|  | Effect hypothesis and mechanism (1): Increases *Workflow fit* by increasing *Compatibility* with existing ways of working (ripple effect)*.* | Supporting paper IDs (n=14): 1271, 2841, 3351, 4222, 5357, 7049, 7050, 7194, S117, S118, S127, S132, S62, S67, S81  GRADE-CERQual rating: High |
| *Cost* | Description: The perceived cost of the intervention. This may relate to costs of time, human or financial resources. May correlate with *Conducted by recipients* and *Active delivery* (face-to-face), as this often equates to high costs, and also with *Resource* because organisations with lower resources will be less likely to ‘afford’ higher costs. | Supporting paper IDs (n=22): 1271, 1591, 187, 1948, 5857, 6087, 627, 7816, S1, S105, S109, S112, S118, S120, S127, S132, S136, S14, S15, S154, S16, S25. |
|  | Effect hypothesis and mechanism (1): High costs can inhibit *Data collection and analysis, Interaction, Acceptance, Intention,* and *Behaviour* by decreasing *Resource match.* | Supporting paper IDs (n=19): 1271, 1591, 187, 1948, 5857, 6087, 7816, S1, S105, S109, S120, S127, S132, S136, S14, S15, S154, S16, S25  Theory: Consolidated Framework for Implementation Research (Cost); Fit between Individuals, Task and Technology framework (Cost)  GRADE-CERQual rating: High |
| *Homophily* | Description: The degree to which feedback intervention recipients view the intervention is used or endorsed by individuals who share their educational, professional, and cultural backgrounds. | Supporting paper IDs (n=3): 5357, 7783, S154 |
|  | Effect hypothesis and mechanism (1): Facilitates *Data collection and analysis* and *Feedback* (when *Conducted by recipients*), *Interaction, Acceptance, Intention,* and *Behaviour* by increasing *Credibility* and *Social influence* (Liking). | GRADE-CERQual rating: Moderate  Reason: Moderate concerns regarding the adequacy and relevance of findings.  Supporting paper IDs (n=3): 5357, S132, S154  Theories: Persuasion theory (Liking), Diffusion of innovations (Homophily) |
| *Linkage at the development stage* | Description: The feedback recipients have input into the design and implementation of the feedback intervention. | Supporting paper IDs (n=11): 2794, 7025, 7694, 8249, S109, S112, S117, S30, S38, S62, S81 |
|  | Effect hypothesis and mechanism (1): Facilitates *Goal setting*, *Data collection and analysis, Interaction, Acceptance, Intention, Behaviour,* and *Performance* by increasing *Compatibility* (with recipients’ *motivation* and organisational goals and systems), *Credibility*, and *Relative advantage.* | Supporting paper IDs (n=9): 7025, 7694, 8249, S109, S112, S117, S30, S62, S81  Theories: Diffusion of innovations (Linkage at the development stage)  GRADE-CERQual rating: Moderate  Reasons: Moderate concerns regarding the coherence of findings. |
| *Observability* | Description: The benefits of the feedback intervention are visible to feedback recipients. Correlates with *Trend* (via a ripple effect), as this is one way of demonstrating the benefits of involvement with an feedback intervention. | Supporting paper IDs (n=12): 3351, 5033, 5357, 5532, 5857, 7025, 7049, 7050, 7694, S132, S4, S7. |
|  | Effect hypothesis and mechanism (1): Facilitates *Goal setting*, *Data collection and analysis* (when *Conducted collected by recipients*)*, Interaction, Acceptance, Intention, Behaviour,* and *Performance* by increasing *Relative advantage* (making its advantages more visible), *Compatibility,* and *Credibility*. | Supporting paper IDs (n=12): 3351, 5033, 5357, 5532, 5857, 7025, 7049, 7050, 7694, S132, S4, S7  Theories: Diffusion of innovations (Observability); Self-efficacy theory (‘performance accomplishments’ – if based on the recipients’ prior improvements e.g. via *Trend*, or ‘vicarious experience’ based on others)  GRADE-CERQual rating: High |
|  | Effect hypothesis and mechanism (2): Increases *feedback intervention attitude* (ripple effect) by increasing *Relative advantage.* | Supporting paper IDs (n=4): 7025, 7049, 7050, S132  GRADE-CERQual rating: Moderate  Reason: Serious concerns regarding the adequacy of findings. |
|  | Effect hypothesis and mechanism (3): Increases positive *Emotion* (ripple effect) by increasing *Relative advantage.* | Supporting paper IDs (n=3): 5532, S132, S4  GRADE-CERQual rating: Low  Reason: Serious concerns regarding the adequacy and coherence of findings. |
| *Ownership* | Description: The degree of perceived ownership of the feedback intervention the recipients have, versus how much they feel it has been imposed on them. Correlates with *Function* and *Reporting* as they often co-exist. | Supporting paper IDs (n=20): 1948, 2023, 2249, 5033, 5357, 6087, 7025, 7194, 7816, 8249, S1, S112, S118, S127, S136, S15, S19, S30, S38, S4, S67, S81 |
|  | Effect hypothesis and mechanism (1a – effect tension): Greater ownership facilitates *Goal setting*, *Data collection and analysis* (when *Conducted collected by recipients*)*, Interaction, Acceptance, Intention, Behaviour,* and *Performance* by increasing *Compatibility* (with recipients’ motivation to provide high quality care, and their sense of autonomy), and therefore also its *Relative advantage.* | Supporting paper IDs (n=11): 2023, 2249, 5033, 5357, 6087, 7025, 7816, 8249, S118, S19, S81  Theories: Self-determination Theory (Autonomy); COM-B System (Motivation); Motivation-Opportunities-Abilities Model (Beliefs) [Disagrees with the theories in effect hypothesis and mechanism 1b]  GRADE-CERQual rating: High |
|  | Effect hypothesis and mechanism (1b – effect tension): Less ownership and forced implementation facilitates *Goal setting*, *Data collection and analysis* (when *Conducted collected by recipients*)*, Interaction, Acceptance, Intention, Behaviour,* and *Performance* via *Social influence* (with recipients responding to authority figures). | Supporting paper IDs (n=5): 1948, 5033, S81, S136, S15, S4.  Theories: Diffusion of innovations (Political directives), Consolidated Framework for Implementation Research (External Policy & Incentives); Persuasion theory (Authority) [Disagrees with the theories in effect hypothesis and mechanism 1a]  GRADE-CERQual rating: Moderate  Reasons: Concerns regarding the adequacy and methodological limitations of findings |
|  | Effect hypothesis and mechanism (2): Increases positive *Emotions* (ripple effect) by increasing *Compatibility* (with recipients’ autonomy and motivation to provide high quality care)*.* | Supporting paper IDs (n=4): 1948, S38, S67, S81  GRADE-CERQual rating: Moderate  Reasons: Minor concerns regarding the adequacy of findings |
| *Training and support* | Description: Training and support is provided to feedback intervention recipients regarding the intervention (not the clinical topic as in *Clinical education*). | Supporting paper IDs (n=24): 2794, 3351, 4222, 5033, 5235, 5857, 627, 7025, 7049, 7050, 7816, S118, S120, S127, S132, S15, S154, S175, S176, S30, S33, S38, S6, S62. |
|  | Effect hypothesis and mechanism (1): Increases *Knowledge and skills in quality improvement* (ripple effect) by increasing *Resource match* and *Actionability.* | Supporting paper IDs (n=21): 3351, 4222, 5033, 5235, 5857, 627, 7025, 7049, 7050, 7816, S118, S120, S127, S132, S15, S154, S175, S176, S33, S38, S62  Theories: Diffusion of innovations (Assessment of implications); Social Learning Theory (Matched-dependent behaviour); Fit between Individuals, Task and Technology framework; Task-Technology-Fit Model  GRADE-CERQual rating: High |
|  | Effect hypothesis and mechanism (2): Increases *Teamwork* (ripple effect), especially if educational sessions delivered to a multidisciplinary group. | Supporting paper IDs (n=2): 5357, S176  GRADE-CERQual rating: Low  Reasons: Serious concerns regarding methodological limitations, and coherence and adequacy of findings |

**Mechanisms**

| **Name** | **Description** | **Evidence** |
| --- | --- | --- |
| *Complexity* | Description: The difficulty of performing the feedback intervention processes.  Effect: Greater Complexity inhibits feedback intervention processes.  Supports proposition 1 – Capacity limitations: the simpler a feedback intervention is to engage with, the less resource it requires. | Supporting paper IDs (n=32): 187, 2794, 2841, 2857, 5235, 5532, 5857, 7194, 7783, 8167, 8249, S1, S104, S105, S112, S118, S120, S127, S132, S136, S15, S154, S16, S175, S19, S25, S30, S38, S6, S62, S71, S81  Theories: Diffusion of innovations (Complexity); Consolidated Framework for Implementation Research (Complexity; Task-Technology-Fit Model (Task complexity); Fit between Individuals, Task and Technology framework (Task complexity)  GRADE-CERQual rating: High |
| *Relative advantage* | Description: Recipients’ perceived benefits of the feedback intervention, often when compared to alternative existing or proposed ways of working. Aspects of an feedback intervention considered to have a relative advantage are (understandably) situation-specific, so its appearance as a mediating variable is inconsistent.  Effect: Greater Relative advantage facilitates feedback intervention processes.  Supports proposition 1 – Capacity limitations: the more a feedback intervention has a perceived advantage over current ways of working, the more likely it is to be adopted within the available resources. | Supporting paper IDs (n=51): 1271, 1591, 187, 2023, 2249, 2794, 2841, 2857, 3351, 5033, 512, 5357, 5532, 5857, 6087, 7025, 7049, 7050, 7301, 7694, 7816, 8249, S1, S105, S109, S112, S117, S118, S120, S127, S132, S136, S14, S15, S154, S158, S159, S16, S17, S175, S176, S19, S25, S28, S30, S32, S38, S52, S6, S67, S7.  Theories: Diffusion of innovations (Relative advantage); Consolidated Framework for Implementation Research (Relative advantage); Model of Information Systems Success (Net benefits); Technology Acceptance Model (Usefulness).  GRADE-CERQual rating: High |
| *Resource match* | Description: Whether the associated costs of the feedback intervention are matched by the available resource.  Effect: Greater facilitates feedback intervention processes.  Supports propositions 1 – Capacity limitations: if a health care organisation’s resources match the costs of implementing an feedback intervention they are more likely to engage and respond to it. | Supporting paper IDs (n=31): 2841, 4222, 7049, 7194, 7816, S1, S104, S105, S109, S112, S118, S120, S127, S132, S136, S14, S15, S154, S158, S159, S16, S17, S175, S176, S19, S25, S30, S38, S62, S67, S81.  Theories: Consolidated Framework for Implementation Research (Available resources); Diffusion of innovations in health service delivery and organisation (Dedicated time and resources)  GRADE-CERQual rating: High |
| *Compatibility* | Description: The degree of ‘fit’ between the feedback intervention and characteristics of the recipient and their organisation e.g. beliefs, norms, values, culture, structures, processes, technical systems.  Effect: Greater Compatibility facilitates feedback intervention processes.  Supports proposition 2 – Identity and culture: the more a feedback intervention can align with the beliefs, systems, and processes of an organisation and its staff, the greater its relevance, and the less disruption required for its implementation. | Supporting paper IDs (n=55): 1271, 1591, 187, 1948, 2023, 2249, 2794, 2841, 2857, 3351, 4222, 5033, 512, 5357, 5532, 5857, 6087, 627, 7049, 7050, 7194, 7301, 7694, 7816, 8167, 8249, S1, S105, S112, S117, S118, S120, S127, S132, S136, S14, S15, S154, S16, S17, S175, S176, S19, S25, S28, S30, S32, S38, S52, S6, S62, S67, S7, S71, S81.  Theories: Diffusion of innovations in health service delivery and organisation (Compatibility); Consolidated Framework for Implementation Research (Compatibility); Fit between Individuals, Task and Technology framework (Fit); Task-Technology-Fit Model (Fit).  GRADE-CERQual rating: High |
| *Credibility* | Description: The perceived trustworthiness and reliability of the feedback intervention.  Effect: Greater Credibility facilitates feedback intervention processes.  Supports proposition 2 – Identity and culture: the more trustworthy and reliable a feedback intervention, the more likely they are to believe it will help them improve patient care. | Supporting paper IDs (n=42): 1271, 1591, 187, 1948, 2023, 2249, 2794, 2857, 4222, 5033, 5357, 5857, 6087, 7049, 7050, 7194, 7301, 7694, 7783, 7816, 8167, 8249, S1, S104, S109, S112, S117, S127, S132, S136, S14, S154, S16, S17, S28, S30, S38, S6, S67, S7, S71, S81  Theories: Individual Feedback Theory (Credibility)  GRADE-CERQual rating: High |
| *Social influence* | Description: Interpersonal processes that cause feedback intervention recipients to change their thoughts, feelings, or behaviours. Key aspects include: 1) Competition (between health professionals), 2) Social proof (their desire to behave in the same way as other health professionals), 3) Authority (to obey credible authority figures), 4) Liking (persuaded by people they like), and 6) Reference group (where health professionals feel part of a group and will change their behaviour if they believe their membership of that group is threatened).  Effect: Greater Social influence facilitates feedback intervention processes.  Supports propositions 3 – Identity and culture: the more a feedback intervention can harness the social dynamics between health professionals, the more likely it is to be implemented. | Supporting paper IDs (n=15): 2857, 5033, 7783, S104, S117, S127, S150, S176, S19, S25, S32, S4, S6, S62, S7  Theories: Social Comparison Theory (i.e. competition), Persuasion Theory (i.e. Social Proof, Authority, and Liking), Reference group theory, Normative Social Influence, Social Learning Theory, Social Norms Theory.  GRADE-CERQual rating: High |
| *Actionability* | Description: The feedback message’s ability to directly facilitate behaviours in feedback intervention recipients.  Effect: Greater Actionability tends to facilitate feedback intervention processes.  Supports proposition 3 – Behavioural induction: the more a feedback intervention can successfully and directly support clinical behaviours for individual patients are most effective. | Supporting paper IDs (n=37): 1271, 1591, 187, 1948, 2249, 3351, 4222, 5033, 5532, 5857, 7025, 7049, 7301, 7783, S1, S104, S105, S109, S112, S117, S118, S127, S132, S15, S150, S154, S158, S159, S16, S175, S25, S30, S38, S6, S62, S71, S81  GRADE-CERQual rating: High |
