# Supplementary material for: Clinical Performance Feedback Intervention Theory (CP-FIT): a new theory for designing, implementing, and evaluating feedback in health care based on a systematic review and meta-synthesis of qualitative research
Source: Implement Sci. 2019 Apr 26;14:40. doi: 10.1186/s13012-019-0883-5 (PMC6486695; doi:10.1186/s13012-019-0883-5)
Supplement: Supplementary file 6 — CP-FIT case studies. (DOCX 933 kb) [file 13012_2019_883_MOESM6_ESM.docx]

**Additional file 6: Three case studies of different types of feedback intervention explained by Clinical Performance Feedback Intervention Theory (CP-FIT)**

**Case study 1. “Audit and Feedback”: Pharmacist-led information technology-enabled (PINCER) intervention** [1]

**Setting and topic**

Primary care practices (England); Medication safety.

**Effectiveness**

Effective at reducing proportions of patients at risk of medication safety errors in a randomised controlled trial [2].

**Description of feedback intervention**

Pharmacists were allocated to GP practices for three days per week for up to 12 weeks. They conducted an educational session at the beginning of the intervention period on the importance of medication safety. They subsequently provided population-level feedback and lists of patients at risk of medication safety errors to the GP practices (e.g. patients with asthma also prescribed beta-blockers) both verbally in face-to-face meetings and in written document form. Pharmacists used root cause analysis techniques to identify potential causes of clinically important errors in medicines management, and to assist practices in making changes to patients’ medication.

**Key findings from qualitative study [1] explained by CP-FIT (Figure 1)**

GP practice staff believed the medication safety topic had both *Importance* and *Relevance* (hypotheses 1 and 3 respectively, Table 5 in main manuscript), facilitating *Intention* and *Acceptance*:

*“All GPs and their teams recognized that prescribing errors were an important and potentially preventable problem.”*

The use of *Patient lists* (hypothesis 9) was key to identifying patients in whom action was required, facilitating *Intention* and *Verification*:

*“GPs felt it was difficult to comment [on potential medication safety errors] without knowing the identity of the patients and their history.”*

The pharmacists were perceived as having sufficient *Source knowledge and skill* (hypothesis 17) to deliver feedback, also facilitating *Intention* and *Acceptance*:

*“There was a widely held belief that a pharmacist-centered intervention was a credible solution. ‘I think pharmacists are obviously much, much better informed than we are…Pharmacists will tend to often be a lot more evidence based in what they’re telling us and will have looked into it in a bit more detail rather than their own personal preferences. So it’s almost a more, I think it’s almost a more reliable source of information about drugs when we’re trying to make decisions about whether we’re needing to alter our practice.’ (GP 2, PINCER intervention practice, Focus Group 1, Location 1).”*

Practice staff often struggled to find the *Resource* (hypothesis 24) in terms of time or staff to take action based on the feedback (*Behaviour*) due to *Competing priorities* (hypothesis 25):

*“The busy practice environment meant that there were often conflicting priorities within practices. ‘I think they’ve got so many priorities and in that particular case they were a single-handed practice, erm, so many priorities that erm, some of the issues that, you know, with PINCER such as monitoring erm, are possibly not top of the list.’ (Trial Pharmacist, Brief Interview 9, Location 2).”*

However, the pharmacists counteracted this by acting as *External change agents* (hypothesis 37) to provide additional capacity through which most improvement action took place.

*“Pharmacists in all PINCER intervention practices were therefore highly valued and given the authority to address many of the issues identified themselves… PINCER pharmacists may be viewed as ‘change agents’. In the diffusion of innovations literature, these are individuals who influence clients’ innovation decisions in a direction deemed desirable by a ‘change agency’. It is the change agency’s aim to implement the innovation with a focus on the collective goals of the social system (here to improve prescribing safety).”*

Overall, despite the inhibiting effects of reduced *Resource* and increased *Competing priorities* on *Behaviour*, the facilitating effects of PINCER’s *Importance, Relevance, Patient lists, Source knowledge and skill,* and *External change agents* was enough to ensure that all essential feedback cycle processes were successful (Figure 1). This explains why PINCER was effective at reducing proportions of patients at risk of medication safety errors.

**Figure 1: How CP-FIT explains PINCER’s effectiveness**

Green and red arrows = facilitating and inhibiting effects on feedback processes respectively; +/- = the presence of absence of a variable; black solid arrows = strong feedback cycle pathways. All essential feedback cycle processes are successful. Thus PINCER was effective.


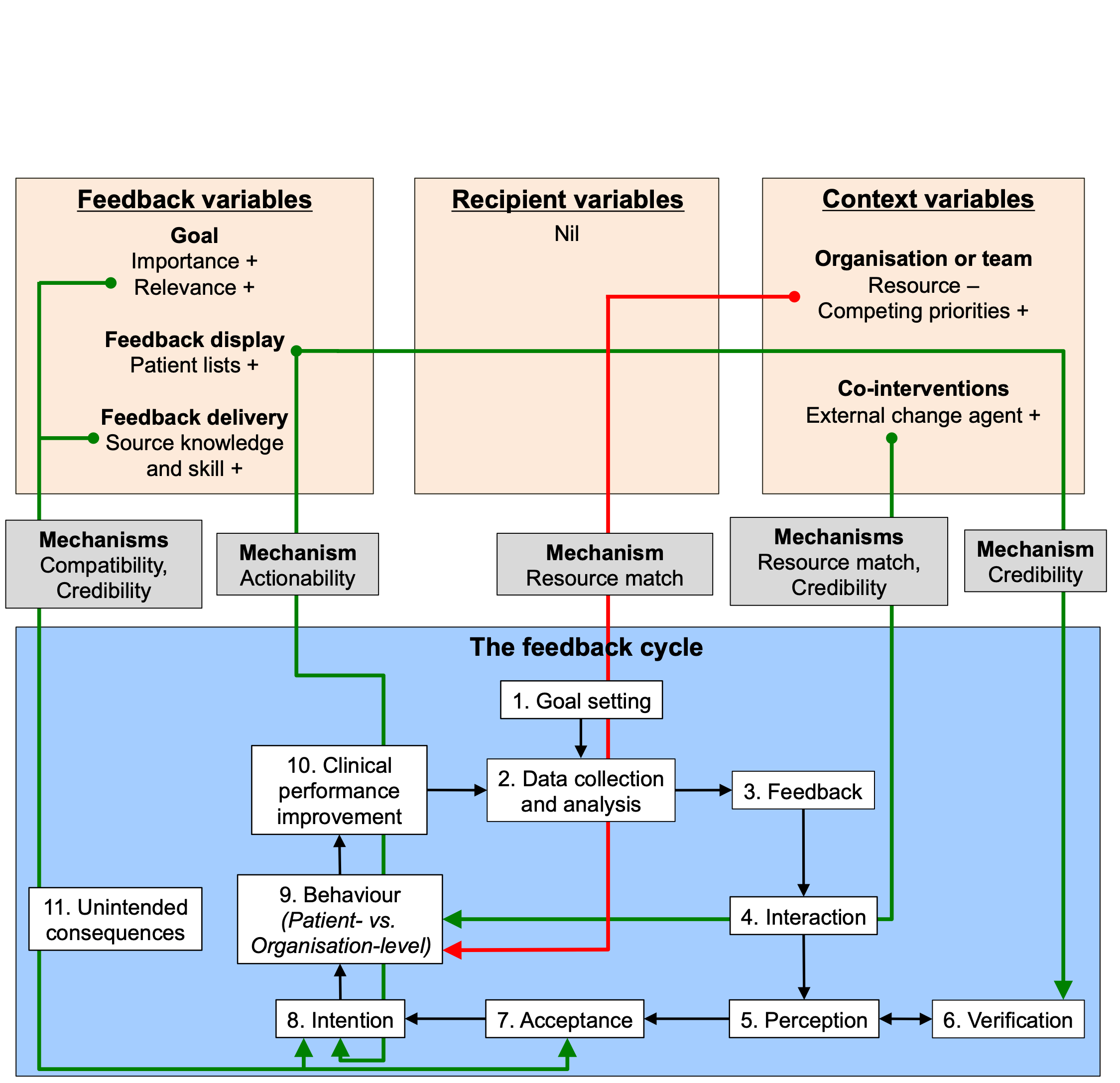


**Case Study 2. “Performance Measurement”: Clinical Resource and Audit Group (CRAG) indicators** [3]

**Setting and topic**

Secondary care trusts (Scotland); Patient outcomes.

**Effectiveness**

“No obvious impact” in improving patient outcomes as inferred by study authors [3]**:**

*“The CRAG indicators had a “low profile” in all trusts and were rarely cited by staff as the primary drivers of quality improvement or sharing best practice between organisations. In six trusts the CRAG indicators were reported to have stimulated some action in relation to breast cancer or stroke services, but such action was restricted to checking and auditing the quality of the data rather than direct action to improve delivery of service”*

**Description of feedback intervention**

CRAG indicators were compiled and disseminated by Scottish National Health Service managers. They included 38 clinical indicators detailing patient outcomes for each hospital based on a variety of data sources. To minimise random variation each indicator spanned a period of at least three years, and were case-mix adjusted. Typical indicators included: five-year survival in women with breast cancer, and 30-day survival after emergency admission for stroke. Feedback was provided in the form of a published document.

**Key findings from qualitative study [3] explained by CP-FIT (Figure 2)**

There was no *Active delivery* of feedback documents to front-line staff (hypothesis 18, Table 5 in main manuscript) reducing *Interaction*:

*“We found that although consultants and chief executives were aware of the data, most nurse managers and junior doctors reported that they had little or no knowledge of the indicators. Only one trust disseminated these data to nurse managers and junior doctors… “There should be more widespread dissemination of this information [the CRAG reports]. It would certainly be useful to push it down to my level of service manager… Clinical outcomes don’t just apply to doctors” (nurse manager)”*

Significant lags in *Timeliness* between data collection and feedback (hypothesis 11), and lack of feedback *Specificity* regarding individual clinician performance (hypothesis 10), inhibited recipients’ *Intention* to act on it:

*“The elapsed time between collection and publication of data was a major drawback to the indicators being used in a meaningful way for continuous quality improvement. In many cases the CRAG indicators are at least a year out of date and considerably more for some indicators such as breast cancer. “It’s pretty basic information and it comes out several years after it is taken. Things have changed over that period of time. So, in relation to say treatment of cervical cancer, the whole way of cancer management has changed. The change had already occurred by the time the data were issued” (medical director)”*

*““I don’t think at the current level of accuracy you can pull out that sort of information [individual clinical performance] from these figures. Poor performance with doctors tends to be [transmitted] from word of mouth and other soft information” (stroke consultant)”*

Concerns amongst recipients that feedback data had low *Accuracy* (hypothesis 6) reduced feedback *Acceptance*:

*“Many staff, in particular the consultants, had serious concerns over the quality of the data used to compile the CRAG indicators, and because of this the data lacked credibility. Problems centred on issues of data quality, including incomplete and inconsistent coding and inadequate adjustment for variation in case mix. “One concern is how valid the data are. It is important if you are going to use data that you have clinical people on board and that they are happy the data collection is correct. We have a degree of suspicion over some of the CRAG data that are coming out” (stroke consultant)”*

Low levels of *Knowledge and skills in quality improvement* amongst recipients (hypothesis 21), and no provision of *Training and support* (hypothesis 39), reduced health professionals’ *Perception* (understanding) and *Intention* to act on the feedback:

*None of the trusts ran specific training or education programmes on the appropriate use and interpretation of clinical indicators, and no single person within each trust was identified as being responsible for supporting their use throughout the organisation. “I don’t think there is sufficient knowledge about CRAG data. It is not taught in medical schools” (breast surgeon)”*

Overall, the CRAG indicators’ reduced *Active delivery*, *Timeliness, Specificity,* and *Accuracy,* coupled with health professionals’ low levels of *Knowledge and skills in quality improvement* and no provision of *Training and support* meant that *Interaction, Perception, Acceptance,* and *Intention* feedback processes were all weakened, with the *Intention* process completely failing. This halted progress around the feedback cycle at that point, explaining why the CRAG indicators were ineffective at improving patient outcomes (Figure 2).

**Figure 2: How CP-FIT explains the CRAG indicator’s lack of effectiveness**

Red arrows = inhibiting effects on feedback processes; +/- = presence of absence of a variable; black solid arrows = strong feedback cycle pathways; dotted arrows = weakened feedback cycle pathways. *Interaction, Perception, Acceptance,* and *Intention* feedback processes are all reduced, completely halting progress around the cycle at *Intention.* Thus the CRAG indicators were ineffective.


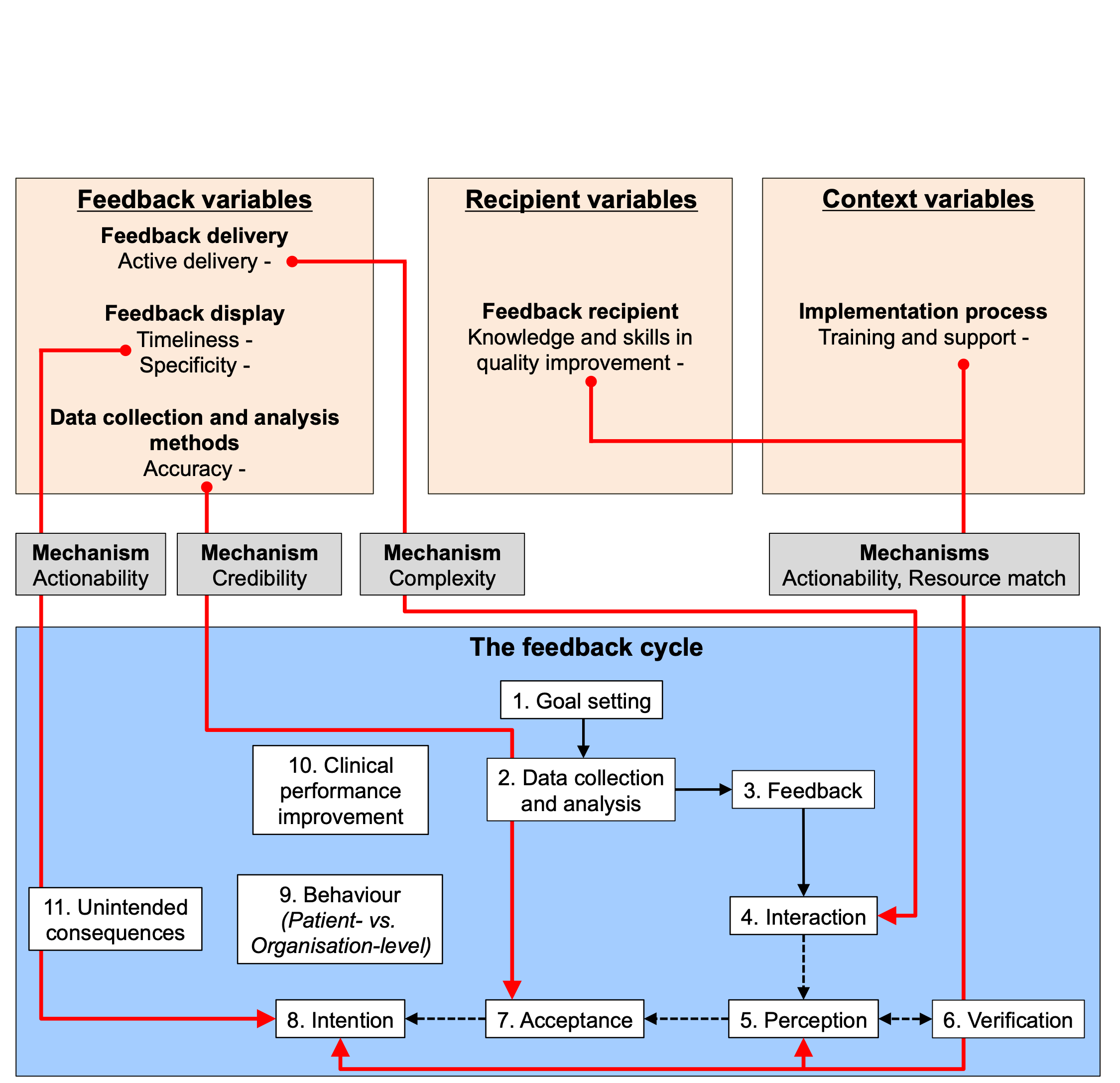


**Case study 3. “Quality Dashboard”: The Prescribing, Information and Communication System (PICS)** [4]

**Setting and topic**

Secondary care hospital (England); Prescribing.

**Effectiveness**

Ineffective at reducing the number of prescription and laboratory alerts ignored by junior doctors in a randomised controlled trial [4].

**Description of feedback intervention**

Clinical performance data were collected from a Clinical Decision Support System (CDSS) embedded in an electronic health record. The CDSS alerted users when medication was prescribed that contravened local guidelines, or when a patient’s laboratory test results required attention. Alerts could be actioned or ignored. The proportion of ignored alerts per month was fed back to junior doctors in a web-based dashboard using tables and graphical charts. Email reminders with a link to the dashboard were sent every week.

**Key findings from qualitative study [4] explained by CP-FIT (Figure 3)**

On the positive side, the dashboard *Improved knowledge and awareness* (Additional file 5) of recipients about prescribing issues in general (Unintended consequence, Figure 3):

*“I think that’s the only thing that to be honest I kind of took away which was that I was more conscientious about my prescribing. When I see the levels go sort of high that’s when I sort of started making sure my dosing was correct and that the drug history was correct and that I was looking at interactions and was cautious about allergy status so I was just saying as a whole it flags up to me that you know to be actually a bit more cautious.” (Individual interview 4)*

However, senior doctors instructing the junior doctors often initiated prescriptions that generated alerts. Consequently, the junior doctors felt the feedback had poor *Accuracy* (hypothesis 6, Table 5 in main manuscript), and little *Controllability* (hypothesis 2), which reduced their *Acceptance* of, and *Intention* to act on, the feedback, respectively:

*“Junior doctors are learners in the clinical environment and the data indicate that the constraints placed on their clinical independence means that they often carry out actions that have been ordered by senior doctors. Thus junior doctors perceived the feedback provided via the dashboard to be an inaccurate account of their own clinical practice given that actions that generate alarms, alerts and warnings in PICS against their log-on IDs had not necessarily been initiated by them, but ordered by senior doctors. “Decisions to put patients on drugs isn’t really down to us anyway. I wouldn’t say ‘start a patient on laxatives or painkillers’, but then other than emergency treatment I never really start a patient on drugs by my own means. I will always go through a senior doctor… So are you looking at the right cohort as to who makes the decisions?” (Individual Interview 3)”*

The junior doctors often received alerts about patients who were not under their care, meaning they carried little *Relevance* (hypothesis 3), thus reducing their *Intention* to act on the feedback:

*“’Sometimes I think it pressured me into ticking off things that maybe I shouldn’t have been ticking off, particularly when you’re doing say night cover so (…) every ward you get on to you get flashed up a selection of lab alarms about patients you’ve never met so it’s not really appropriate to be accepting those because you don’t know anything about any of them (…) you don’t really feel like that’s my responsibility and yet at the same time, I’m ignoring lab alerts.” (Individual Interview 2) “Overnight when I do nights and things flash up and it’s in the relevant directorate (…) then clearly I can’t click ‘ignore’ because that is my responsibility so I go and deal with it, whatever that alert might be. But during the day, you know if things start flashing up and it’s not my patient…you know there’s a lot of patients in this hospital. I’m not going to respond to everything…”(Individual Interview 5)”*

Clinical emergencies were *Competing priorities* (hypothesis 25) and junior doctors would ignore the alerts during them to save time, reducing their response (*Behaviour*) to the feedback:

*“Doctors’ priorities change in emergencies, and they are less likely to sign off alerts”*

In clinical emergencies alerts consequently had low *Clinical appropriateness* (hypothesis 33). *Clinical appropriateness* of the feedback was also often reduced because local clinical guidelines did not apply to specific patient groups, thus reducing the junior doctors’ *Intention* to act on the feedback:

*“In other instances there are limitations due to systemic constraints which do not allow for flexibility in prescribing practices in specific clinical contexts where deviation from recommended doses may be clinically indicated. “Military patients have a set pain protocol which involves (…) prescribing a number of opioids. So every time that I put somebody on this pain protocol, I get a red alert saying ‘multiple opioid drugs prescribed, are sure you want to proceed?’, so I tick yes but obviously then on the dashboard I will get a negative mark if you like.” (Individual Interview 6)”*

Despite PICS resulting in *Improved knowledge and awareness* about prescribing issues, its feedback’s low *Accuracy, Controllability, Relevance,* and *Clinical appropriateness* to some patients*,* coupled with *Competing priorities* in the hospital meant that *Acceptance* and *Intention* feedback processes were all weakened, with the *Behaviour* process completely failing. This halted progress around the feedback cycle at that point, explaining why PICS was ineffective at reducing the number of prescription and laboratory alerts ignored by junior doctors (Figure 2).

**Figure 3: How CP-FIT explains PICS’ lack of effectiveness**

Red arrows = inhibiting effects on feedback processes; +/- = presence of absence of a variable; black solid arrows = strong feedback cycle pathways; dotted arrows = weakened feedback cycle pathways. *Acceptance* and *Intention* feedback processes are all reduced, completely halting progress around the cycle at *Behaviour*. Thus PICS was ineffective.


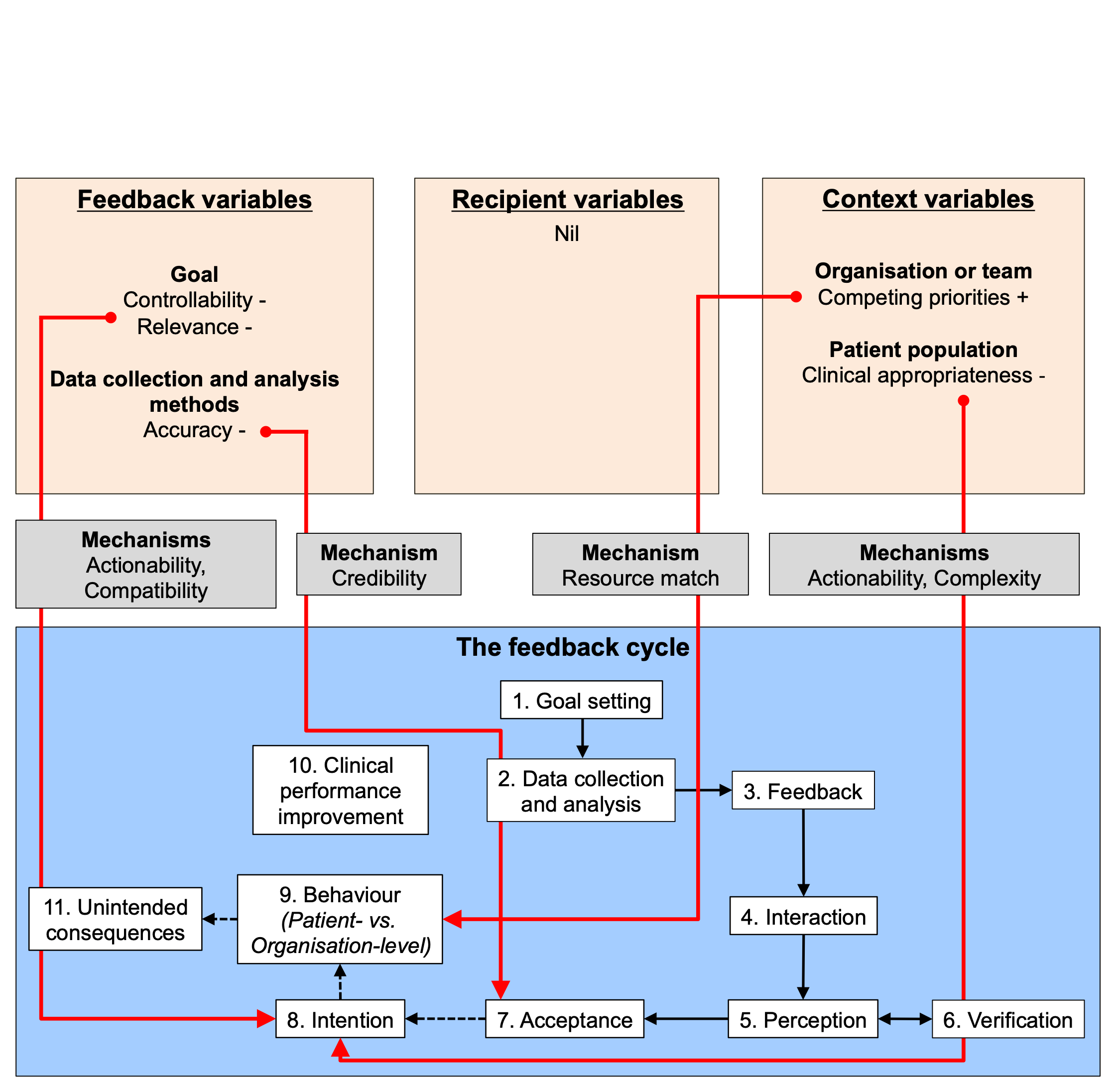


**References**

[1] K.M. Cresswell, S. Sadler, S. Rodgers, A. Avery, J. Cantrill, S. a Murray, et al., An embedded longitudinal multi-faceted qualitative evaluation of a complex cluster randomized controlled trial aiming to reduce clinically important errors in medicines management in general practice., Trials. 13 (2012) 78. doi:10.1186/1745-6215-13-78.

[2] A.J. Avery, S. Rodgers, J. a Cantrill, S. Armstrong, K. Cresswell, M. Eden, et al., A pharmacist-led information technology intervention for medication errors (PINCER): a multicentre, cluster randomised, controlled trial and cost-effectiveness analysis., Lancet. 379 (2012) 1310–9. doi:10.1016/S0140-6736(11)61817-5.

[3] R. Mannion, M. Goddard, Impact of published clinical outcomes data: case study in NHS hospital trusts., BMJ. 323 (2001) 260–3.

[4] S. Redwood, N.B. Ngwenya, J. Hodson, R.E. Ferner, J.J. Coleman, Effects of a computerized feedback intervention on safety performance by junior doctors: results from a randomized mixed method study, BMC Med Inf. Decis Mak. 13 (2013) 63. doi:10.1186/1472-6947-13-63.
